# Supplementary material for: Goal-oriented care for patients with chronic conditions or multimorbidity in primary care: A scoping review and concept analysis
Source: PLoS One. 2022 Feb 4;17(2):e0262843. doi: 10.1371/journal.pone.0262843 (PMC8815876; doi:10.1371/journal.pone.0262843)
Supplement: S1 Table — (PDF) [file pone.0262843.s001.pdf]

SUPPLEMENTARY Table 1

Table 1 Overview preliminary version attributes

Goal-elicitation
- Three main components to make goal-setting effective: were patient and GP engagement, preparation, supportive goal-elicitation and collaborative action planning [23].<br>- Effective goals elicitation is geared to each patient's state of readiness [1].<br>- Allowing clients to tell their stories and reflect on their achievements may facilitate elicitation of client-centered goals [24].<br>- Goal-elicitation was a shared process that involved relationship building between patient and GP [23]<br>- Elicitating goal-directed conversations may lead to more tailored and effective care and has been shown that enabling patients in such a way increase adherences to evidence-based guidelines and patient satisfaction [47]

Goal-setting
- Goal-setting may be useful to facilitate patient-centered communication for patients with multimorbidity [23]<br>- Explicit focus on goal-setting or priority setting and goals are specifically determined [54]<br>- Goal-setting is promoted as a mechanism for health professionals to engage with patient's psychosocial needs and social context to support individuals in what matters most to them live well with their conditions [32]<br>- Goal-setting is the action of a person who has the confidence, commitment, motivation and knowledge necessary to attain a goal that is specific, challenging, measurable and relevant within specified amount of time [38]

- Goal-setting is a key feature of coordinated care plans intended to support coordination and continuity of care for older adults, and others with complex needs [35]	
Interaction	*Negotiating*<br>- goals of care are negotiated between patient and healthcare provider [6, 23]<br>- translation of the overarching goal into relevant and realistic goals of care is a complex negotiating and balancing act [6]<br>- elaborating clear and concise priorities that inform decision-making is a challenge, requiring a reliable and efficient process for ascertain patient's goals and preferences [4]<br>- the clinician needs to explain what is possible and negotiate potentially achievable goals with the patient [49]<br>- elaborate more until he feels he understand what he is looking for [24]<br>- One of the most important aspects of goal-setting was the ability of the patient to discuss, formulate and agree the goals specific [23]<br>- Goal negotiation involves discussion of any problems, exploration of patient's personal values, needs and capabilities, patient education and deliberation optional goals [37].<br><br>*Collaborative goal-setting*<br>- Collaborative goal-setting is a structured form of patient engagement in which providers discuss and agree on a health goal [4, 39]<br>- collaborative goal-setting is an evidence based process [4]<br>- collaborative action plan [23]<br>- doctors and patients with multimorbidity working together on what really matters to patients [23]

	- patients as active partners [33]<br><br>- finding common grounds [30]<br><br>- collaborative process taking place within goal-setting consultation [23]<br><br>- collaborative goal setting are necessary elements [54]<br><br>- collaborative goal-setting defines as a process by which health care professionals and patients agree on a health-related goal [54]<br><br>- the GP's described their role as collaborative and as a facilitator of the goal-setting process [23]<br><br>- Greater consideration to patient's narratives reflecting their lived experiences [40]<br><br>- Process by which caregiver and patient agree on a health related goal [50]<br><br>- Personal target of the patients are discussed, while taking into account the individual patient's needs, preferences and abilities [2]<br><br>- A unique interplay between the patient, the clinician, and the goals developed collaboratively both the patient and the clinician [49]<br><br>*Balance between patient and provider*<br><br>- involvement of patients, both in relation to the choice of intervention [6, 24]<br><br>- providers help patients to set measurable goal for their chosen condition [39]<br><br>- Shift in power from the therapist to the client and facilitated goal identification [24]<br><br>- it is allowed to drop by the therapist and the decision was accepted by the client [24]<br><br>- Together with the person, professionals make the overarching goal as drive for decision making [6]

	- patients are encouraged to share their priorities with their clinicians and prompt their clinicians to consider how currently recommended care aligns with the patient's health priorities [4]<br>- Shift in power due the fact that provider accomplish the achieved goals of the patients [24]<br>- GP's role had been a form of moral support and seemed to be to listen, validate and support patient to articulate their goals rather than take actions [23]<br>- Patient and providers put themselves in the shoes of the other to understand the other constraints [44]<br>- Final decisions should be made by the individual [13]<br>- A goal-directed approach would create more equality in relationships between clinicians and patients [39]
Goals	*Person-centered goals*<br>- Person legitimate what the overarching goal should be [6]<br>- person identifies his or her core values [1, 4]<br>- prompts to articulate which health states are important to them and their relative priority [49]<br>- prioritizing one actionable thing that is most important to the patient [33]<br>active listening, clarify meaning attached to goals and respect clients choice [24]<br>- goals were person-centered and focused on the things that could change [23]<br>- focusing on desired activities rather than eliminating symptoms [33]<br>- Underlying foundation of goals should be the patient's values [41]<br>- Achieving personal goals [55]<br>- Care is personalized to accommodate patient's goals, preferences and resources [12]

	- Interpersonal medicine care is based on the patient's circumstances, capabilities and preferences [47]<br>- Health must be defined by each individual and will therefore be different [13]<br>*Health outcome goals*<br>- identifying priorities provides a means of reducing the tradeoffs between outcome goals, between healthcare preference [4]<br>- what individuals are able and willing to do to achieve their health outcomes and includes activities [1]<br>- health outcome goals are the individual health outcomes that persons hope to achieve through their health care [1]<br>- health outcome goals are distinct from behavioral goals [1]<br>- health outcome goals are patients personalized health outcome priorities [1]<br>- health outcome goals: what they want from their --healthcare and their healthcare preferences, what healthcare activities they are willing and able to perform, and the care they are willing or not willing to receive [4]<br><br>*Overarching goals*<br>- Overarching goal must be translated in an open and non-judgmental process [6]<br>- Overarching goals can be broken down into supporting sub-goals [6]<br>- goals can be disease specific or overarching, across multiple diseases [12]<br>- Setting overarching goals can take longer than setting biomedical goals [41]<br><br>- tentative goals: clients seemed to be considering goals that they were not sure met the therapist's criteria or whether their attainment was a possibility [24]<br>- goals can be objective or subjective [12]

	- goals can be focused on maintaining the status quo or improving the current situation [12]<br><br>*SMART*<br>- Make overarching goals explicit [6]<br>- Participants share clear goals [6]<br>- Person needs, values and preferences [6]<br>- realistic [6, 50]<br>- relevant [6]<br>- requirement that the goal should be interesting and meaningful [24]<br>- underlying foundation of goals should be the patient's values [12]<br>- negotiating goals that are relevant, realistic and observable [6, 23, 30]<br>- not all patients goals may be realistic or attainable [49]<br>- SMART goals [1, 4, 12, 24]<br>- SMART framework is helpful for operationalizing personal goals [46]<br>- To create SMART goal, the patient and provider collaboratively specify the goal itself, the importance of that goal to the patient, perceived achievability of the goal, the timing of the goal and any supports and resourced available [35]
Care plan	*Goal-based care plan*<br>- start with assessing and negotiating the overarching individual goals [6]<br>- patient-priority directed care begins with patients and caregivers identifying and communicating their health outcome goals and treatment and care preferences with the help of a trained and skilled member of the healthcare team [1]<br>- overarching goals defines the scope of the care plan [6]<br>- aligning treatment towards common goals [49]

	- Plan consists sub-goals and sub-tasks [6]<br><br>- Explore what life goals may be and translate into goals relevant for care [6]<br><br>- Goals, wishes and expectations of the patient are the starting point for the care plan [56]<br><br>- Important to align care with patient's personal histories, values and priorities [12]<br><br>*Goal-oriented care plan*<br><br>- aligning healthcare recommendations to achieve specific health outcome goals [4]<br><br>- when your clinicians understand what you want of your health, they can offer you healthcare options that give you the best chance of reaching the goals [1]<br><br>- sets targets for patients and clinicians to plan a course of action and measure success [12]<br><br>- Creating action plans for achieving the goals [50]<br><br>- Planning on how to work on the established goals [76]<br><br>*Personalized care plan ('what matters to you')*<br><br>- prioritizing interventions most important to the patient [30]<br><br>- starting with one actionable thing that matters most and conducting serial trials [33]<br><br>- should meet the overarching personalized goals which reflect 'what matters to the person' [1, 3, 4, 6, 12, 30, 33]<br><br>- patients creates action plans for achieving this goal [39]<br><br>- Plan should be evidence-based, should support health literacy, patient involvement and self-management [6]

	- Patient preferences, needs and values and ensuring that patient values guide all clinical decisions [33]<br><br>*Multidisciplinary approach*<br>- clinician should provide a treatment plan, encouragement and advocacy to help the patient meet agreed on-goals and readdress them if the situation change [49]<br>- Plan should identify roles, tasks, responsibilities [6]<br>- The care plan is based on a multidisciplinary review of the goals [6]<br>- involvement of care givers [54]<br>- the decision process should involve all relevant providers and the patient/ caregivers [6]<br>- it is important to include caregivers, family members and other social support [3]<br>- priorities guide interactions between patients, caregivers and clinicians [4]<br>- The personalized goals are used to identify the multidisciplinary team needed to assess patient's health issues [6]<br>- priorities and preferences are visible in integrating care between multiple treatment providers. Care is consistent with patients outcome goals and preferences [1, 4, 30]<br>- deciding which care within their area of expertise is most likely to help patients achieve their goals [1]<br>- all members of the team, including patients and caregivers, must be willing and able to carry out their agreed- on roles and responsibilities [1]
Care delivery	*Care plan based delivery*<br>- delivered according to the plan [6]

	- providing better care would be to focus on a patient's individual health goals within or across a variety of dimensions [10]<br>- the team and person is responsible for care delivery according to the plan [6]<br>- if possible, begin with simple and quick interventions that address one or more patient goals [1]<br>- bearing witness to patients goals [23]

Goal-evaluation	
Measurable goals	- making goals measurable [23]<br>- priorities and preferences are visible so that measures of care and health policy and service delivery models van value person-focused care [30]<br>- Making goals measurable could overcomplicate and distance the patient from their own goal [23]<br>- Measurement of both the quality and quantity of work necessary to attain the goal and the development of strategies to reach the goals [38]<br>- Goals should be quantified whenever possible and measurable so that progress toward achievement can be monitored [13]
Evaluation of GOC process	- GP valued the process [23]<br>- determine how well these goals are being met [49]<br>- goals may have been met or unmet perhaps because they were too difficult or are no longer relevant or desired [12]<br>- quality of care delivery depends on how well it provides the expected and planned care [6]

Feedback	- Goal evaluation as feedback to all contributors [6]<br>- success or failure of trials should be defined by whether patients achieved their health outcome goals or healthcare preferences [33]<br>- if providers do not access success or failure, then no learning or adjustment will occur [6]<br>- Setting goals include feedback on the performance toward the goal [38]
Continue process	*Ongoing process*<br>- Person needs, values and preferences as they develop over time [6]<br>- goal-oriented process [6]<br>- cyclic process framework [6]<br>- preference elicitation is a constant, iterative process [1]<br>- patient priorities care is a continuous process that begins when patients identify SMART goals<br>- Talking about goals in the context of a continuous relationship between the provider and the patient [41]<br><br>*Reevaluation of the process*<br>- Regular review of goals, plan and goal attainment whenever needed [6]<br>- Goal adjustment and learning for the next cycle [6]<br>- continue to align with their desires and abilities, even if their disease progresses [1, 3]<br>- reevaluation during which goals can be discarded, modified or recalibrated or new goals might be set [12]<br>[33]

	- Goals can be met or unmet perhaps because there were too difficult or are no longer relevant or desired. This leads to reevaluation [41]<br><br>*Duration of the process*<br><br>- care over time [30]<br><br>- patient priority-directed care focuses on current care rather than future care [1]<br><br>- Goals can be focused on maintaining the status quo or improving the current situation

Person context	- Attaining their goals and preferences in the context of their current functioning or life circumstances [3, 12]<br>- understanding the whole person [30]<br>- The context in which interactions occur is important [40]<br>- Goal-oriented care could be tailored to the context of the individual patient [55]<br>- The social and cultural context are taken into account when setting the patient's personal goals and developing the personalized care plan [2]<br>- it is important to set goals from a holistic perspective, tailored to each patient [37]

Person-centeredness	- What matters to you? [1, 6, 23, 33]<br>- being treated as a person and not a diagnose [6]<br>- Developing goals that align with client's values, interests and hopes is therefore a key first step in client-centered practice [24]<br>- taking account of individual preferences as well as their priorities in decisions about care [30]<br>- allowing clients to tell their stories and reflect on their achievement [24]<br>- Health from the viewpoint of the individual [47]
