# Supplementary material for: Goal-oriented care for patients with chronic conditions or multimorbidity in primary care: A scoping review and concept analysis
Source: PLoS One. 2022 Feb 4;17(2):e0262843. doi: 10.1371/journal.pone.0262843 (PMC8815876; doi:10.1371/journal.pone.0262843)
Supplement: S2 Table — (PDF) [file pone.0262843.s002.pdf]

S2 Table 2. Overview training.

Article	Trained population	Training
Blom (2016)	General practicioners and practice nurses	Session 1: theory on care plans using a functional integrative approach. Practicing care plans for own patient and discussing care plans in the group. Planning for the 10 care plans in the study.<br>Session 2: Discuss care plans for own patients. Plan organising the intervention in own practice, allocate responsibilities, care plan making and registering, organising multidisciplinary meetings, evaluating care plans, organising list of community resources for older people.<br>Session 3: Develop an overview of local resources for own region together with practice nurse. Discussion on fall interventions with occupational therapists.
Kangovi (2017)	Primary care providers	60 minute training session on collaborative goal-setting, which reviewed principles of goal-setting theory, including importance of setting realistic goals
Naik (2018)	Advanced practice registred nurse and a member of the healthcare team with case management experience.	Facilitators prepared for training with a face-to-face session in which facilitators practiced the process with a member of the development team and then with a standarized patient.<br><br>Facilitators tested the process with 10 patients, during which time they observed each other and gave feedback.

Salter (2019)	General practicioners from three intervention practices.	3-hour experiental workshop, including a discussion of key principles, skill spotting, using video examples of goal-setting in action, and role-play.
Tinetti (2019)	Ten primary care clincians, cardiologists	Introductory webinar of patient priorities care, participated in two case-based face-to-face training sessions. During these training sessions they role played patient-clinician and clinician-clinician scenarios involving commonly encountered decisional issues for older adults with multiple chronic conditions.
Tinetti (2016)	Member of the clinical team, such as an advanced practice registered nurse or physician assistant	Goals and preferences elicitation requires training and sufficient time to ensure that the goals elicited are SMART.
Vermunt (2017)		Training the professional providing the intervention was explicity mentioned.
