# Supplementary material for: Goal-oriented care for patients with chronic conditions or multimorbidity in primary care: A scoping review and concept analysis
Source: PLoS One. 2022 Feb 4;17(2):e0262843. doi: 10.1371/journal.pone.0262843 (PMC8815876; doi:10.1371/journal.pone.0262843)
Supplement: Attachment — Submitted filename: PONE-D-20-30664_reviewer.pdf [file pone.0262843.s004.pdf]

PLOS ONE

Goal-oriented care in primary care: a concept analysis

--Manuscript Draft--

**Manuscript Number:**	PONE-D-20-30664
**Article Type:**	Research Article
**Full Title:**	Goal-oriented care in primary care: a concept analysis
**Short Title:**	Goal-oriented care in primary care
**Corresponding Author:**	Dagje Boeykens<br>Universiteit Gent Faculteit Geneeskunde en Gezondheidswetenschappen<br>Ghent, BELGIUM
**Keywords:**	goal-oriented care, goal-setting, patient-centeredness, chronic conditions, multimorbidity, concept analysis
**Abstract:**	Background:<br>The healthcare system is faced by an ageing population, increase in chronic conditions and multimorbidity. Multimorbid patients are faced with multiple parallel care processes leading to a risk for fragmented care. These problems relate to the disease-oriented paradigm. In this paradigm the treatment goals can be in contrast with what patients value.<br>The concept of goal-oriented care is proposed as an alternative way of providing care. There is a need to translate this concept into tangible knowledge so providers can better understand and use the concept in clinical practice. The aim of this study is to address this need by means of a concept analysis.<br>Method:<br><br>This concept analysis using the method of Walker and Avant is based on a literature search in PubMed, Embase, Cochrane Library, PsychInfo, CINAHL, OTSeeker and Web of Science. The method provides eight iterative steps: select a concept, determine purpose, determine defining attributes, identify model case, identify additional case, identify antecedents and consequences and define empirical referents.<br><br>Results:<br><br>The analysis of 37 articles revealed that goal-oriented care is a dynamic and iterative process of three stages: goal-elicitation, goal-setting and goal-evaluation. The process is underpinned by the patient's context and values. Provider and patient preparedness are required to provide goal-oriented care. Goal-oriented care has the potential to improve patients' experiences and providers' well-being, to reduce costs and improve the overall population health. The challenge is to identify empirical referents to evaluate the process of goal-oriented care.<br>Conclusion:<br><br>A common understanding of goal-oriented care is presented. Further research should focus on how and what goals are set by the patient, how this knowledge could be translated into a tangible workflow and should support the development of a strategy to evaluate the goal-oriented process of care.
**Order of Authors:**	Dagje Boeykens
	Pauline Boeckxstaens
	An De Sutter
	Lies Lahousse
	Peter Pype
	Patricia De Vriendt
	Dominique Van de Velde
**Additional Information:**	

Powered by Editorial Manager® and ProduXion Manager® from Aries Systems Corporation

Question	Response
**Financial Disclosure**<br><br>Enter a financial disclosure statement that describes the sources of funding for the work included in this submission. Review the submission guidelines for detailed requirements. View published research articles from *PLOS ONE* for specific examples.<br><br>This statement is required for submission and **will appear in the published article** if the submission is accepted. Please make sure it is accurate.<br><br>**Unfunded studies**<br>Enter: *The author(s) received no specific funding for this work.*<br><br>**Funded studies**<br>Enter a statement with the following details:<br>• Initials of the authors who received each award<br>• Grant numbers awarded to each author<br>• The full name of each funder<br>• URL of each funder website<br>• Did the sponsors or funders play any role in the study design, data collection and analysis, decision to publish, or preparation of the manuscript?<br>• **NO** - Include this sentence at the end of your statement: *The funders had no role in study design, data collection and analysis, decision to publish, or preparation of the manuscript.*<br>• **YES** - Specify the role(s) played.<br><br>* typeset	D.B. is a PhD student payed by the King Baudouin Foundation. Grant number: 2019-J5170820-211588 - King Baudouin Foundation - https://www.kbs-frb.be/nl/ - The funders had no role in study design, data collection and analysis, decision to publish, or preparation of the manuscript
**Competing Interests**<br><br>Use the instructions below to enter a competing interest statement for this submission. On behalf of all authors, disclose any competing interests that could be perceived to bias this work—acknowledging all financial support and any other relevant financial or non-financial competing interests.<br><br>This statement **will appear in the published article** if the submission is	The authors have declared that no competing interests exist.

accepted. Please make sure it is accurate. View published research articles from *PLOS ONE* for specific examples.<br><br>**NO authors have competing interests**<br><br>Enter: *The authors have declared that no competing interests exist.*<br><br>**Authors with competing interests**<br><br>Enter competing interest details beginning with this statement:<br><br>*I have read the journal's policy and the authors of this manuscript have the following competing interests: [insert competing interests here]*<br><br>* typeset	
**Ethics Statement**<br><br>Enter an ethics statement for this submission. This statement is required if the study involved:<br><br>• Human participants<br>• Human specimens or tissue<br>• Vertebrate animals or cephalopods<br>• Vertebrate embryos or tissues<br>• Field research<br><br>Write "N/A" if the submission does not require an ethics statement.<br><br>General guidance is provided below. Consult the submission guidelines for detailed instructions. **Make sure that all information entered here is included in the Methods section of the manuscript.**	N/A

**Format for specific study types**<br><br>**Human Subject Research (involving human participants and/or tissue)**<br>• Give the name of the institutional review board or ethics committee that approved the study<br>• Include the approval number and/or a statement indicating approval of this research<br>• Indicate the form of consent obtained (written/oral) or the reason that consent was not obtained (e.g. the data were analyzed anonymously)<br><br>**Animal Research (involving vertebrate animals, embryos or tissues)**<br>• Provide the name of the Institutional Animal Care and Use Committee (IACUC) or other relevant ethics board that reviewed the study protocol, and indicate whether they approved this research or granted a formal waiver of ethical approval<br>• Include an approval number if one was obtained<br>• If the study involved *non-human primates*, add *additional details* about animal welfare and steps taken to ameliorate suffering<br>• If anesthesia, euthanasia, or any kind of animal sacrifice is part of the study, include briefly which substances and/or methods were applied<br><br>**Field Research**<br><br>Include the following details if this study involves the collection of plant, animal, or other materials from a natural setting:<br>• Field permit number<br>• Name of the institution or relevant body that granted permission	
**Data Availability**<br><br>Authors are required to make all data underlying the findings described fully available, without restriction, and from the time of publication. PLOS allows rare exceptions to address legal and ethical concerns. See the PLOS Data Policy and FAQ for detailed information.	Yes - all data are fully available without restriction

A Data Availability Statement describing where the data can be found is required at submission. Your answers to this question constitute the Data Availability Statement and **will be published in the article**, if accepted.<br><br>**Important:** Stating 'data available on request from the author' is not sufficient. If your data are only available upon request, select 'No' for the first question and explain your exceptional situation in the text box.<br><br>Do the authors confirm that all data underlying the findings described in their manuscript are fully available without restriction?	
**Describe where the data may be found in full sentences. If you are copying our sample text, replace any instances of XXX with the appropriate details.**<br><br>• If the data are **held or will be held in a public repository**, include URLs, accession numbers or DOIs. If this information will only be available after acceptance, indicate this by ticking the box below. For example: *All XXX files are available from the XXX database (accession number(s) XXX, XXX.).*<br>• If the data are all contained **within the manuscript and/or Supporting Information files**, enter the following: *All relevant data are within the manuscript and its Supporting Information files.*<br>• If neither of these applies but you are able to provide **details of access elsewhere**, with or without limitations, please do so. For example:<br><br>*Data cannot be shared publicly because of [XXX]. Data are available from the XXX Institutional Data Access / Ethics Committee (contact via XXX) for researchers who meet the criteria for access to confidential data.*<br><br>*The data underlying the results presented in the study are available from (include the name of the third party*	All relevant data are within the manuscript and its Supporting Information Files

and contact information or URL).<br>• This text is appropriate if the data are owned by a third party and authors do not have permission to share the data.<br><br>* typeset	
Additional data availability information:	

Powered by Editorial Manager® and ProduXion Manager® from Aries Systems Corporation

Cover Letter

Click here to access/download;Manuscript;Manuscript.docx

Goal-oriented care in primary care: a concept analysis.

Dagje Boeykens [ab*], Pauline Boeckxstaens [b*], An De Sutter [b], Lies Lahousse [c], Peter Pype [bd], Patricia De Vriendt [aef&], Dominique Van de Velde[ae&], on behalf of the Primary Care Academy[^].

a. Department of Rehabilitation Sciences, Faculty of Medicine and Health Sciences. Ghent University. Ghent. Belgium.
b. Department of Public Health and Primary Care, Faculty of Medicine and Health Sciences. Ghent University. Ghent. Belgium.
c. Department of Bioanalysis, Faculty of Pharmaceutical Sciences, Ghent University. Ghent. Belgium.
d. End-of-Life Care Research Group, Faculty of Medicine and Health Sciences. VUB and Ghent University. Ghent. Belgium.
e. Department of Occupational Therapy. Artevelde University College. Ghent, Belgium.
f. Department of Gerontology and Frailty in Ageing Research Group, Faculty of Medicine and Pharmacy. Vrije Universiteit Brussel. Brussels. Belgium.

* Corresponding author:

E-mail: Dagje.boeykens@ugent.be

*: These authors contributed equally to this work.

&: These authors also contributed equally to this work.

^Membership of the Primary Care Academy is provided in the Acknowledgments.

Abstract

Background

The healthcare system is faced by an ageing population, increase in chronic conditions and multimorbidity. Multimorbid patients are faced with multiple parallel care processes leading to a risk of fragmented care. These problems relate to the disease-oriented paradigm. In this paradigm the treatment goals can be in contrast with what patients value.

The concept of goal-oriented care is proposed as an alternative way of providing care. There is a need to translate this concept into tangible knowledge so providers can better understand and use the concept in clinical practice. The aim of this study is to address this need by means of a concept analysis.

Method

This concept analysis using the method of Walker and Avant is based on a literature search in PubMed, Embase, Cochrane Library, PsychInfo, CINAHL, OTSeeker and Web of Science. The method provides eight iterative steps: select a concept, determine purpose, determine defining attributes, identify model case, identify additional case, identify antecedents and consequences and define empirical referents.

Results

The analysis of 37 articles revealed that goal-oriented care is a dynamic and iterative process of three stages: goal-elicitation, goal-setting and goal-evaluation. The process is underpinned by the patient's context and values. Provider and patient preparedness are required to provide goal-oriented care. Goal-oriented care has the potential to improve patients' experiences and providers' well-being, to reduce costs and improve the overall population health. The challenge is to identify empirical referents to evaluate the process of goal-oriented care.

Conclusion

A common understanding of goal-oriented care is presented. Further research should focus on how and what goals are set by the patient, how this knowledge could be translated into a tangible workflow and should support the development of a strategy to evaluate the goal-oriented process of care.

Keywords

goal-oriented care, goal-setting, patient-centeredness, chronic conditions, multimorbidity, concept analysis

Introduction

The healthcare system is faced by an ageing population and an increase in chronic conditions and multimorbidity (1). More and more people are forced to live with the consequences of these demographic changes and require ongoing (chronic) care on top of acute care (2). At the same time, patient autonomy is gaining importance and patients are considered as an active and important partner in their care (3, 4). Patients with chronic conditions are often consulting multiple health care providers (3) leading to a higher rate of encounters. They also receive a larger amount of prescriptions (5) and they are asked to complete a diverse set of self-monitoring tasks such as managing, exacerbations or monitoring biomedical targets (3). Since patients with (multiple) chronic conditions are faced with multiple parallel care process for their different conditions, there is a considerable risk for fragmented care. Especially when health care providers focus on disease control, patients can experience lack of care continuity and issues with communication as patients themselves focus on the meaning of care and more on personal wellbeing (6, 7). As a result, treatment goals can be in contrast with what patients value in their personal lives (3).

The health care system is oriented towards a disease-oriented paradigm to which many of these problems relate (8-10). In this paradigm, care is mainly organized according to disease-oriented guidelines (10). This may work well for patients with a single disease, but becomes inappropriate for patients with multiple problems. A possible way to overcome many of the challenges is to shift care back from 'what's the matter with the patient' to 'what matters to the patient'. It creates health care processes in which patients' needs are actively sought and met (9). One of the possible strategies is to actively engage patients in identifying their personal goals and aligning care to those goals (11). In 1991, Mold proposed the concept of goal-oriented care as an alternative way for providing care (12). It has been suggested to contribute to patients' wellbeing and quality of life (13). Goal-oriented care as a new paradigm of care has the potential to overcome some of the new challenges for chronical patients (9).

Primary care is often the linchpin of care for these patients (14). It is easy accessible care in which providers address a large majority of health and social needs and develop sustained partnerships with patients in their community (15). Primary care offers a first contact point for new health needs, provides care continuity and care coordination in ongoing and complex cases (16).

Although many primary care providers assume they practice goal-oriented care spontaneously, there is a lack of underpinning knowledge and guidance on how to provide goal-oriented care to patients (13, 17). There is an urgent need to translate the paradigm of goal-oriented care into tangible knowledge so providers can better understand and use this concept in clinical practice. The knowledge gap on goal-oriented care is not only characterized by a lack of in-depth knowledge of the concept. There are also related concepts (such as shared-decision making (18) and patient-centered care (19)) that challenge the common understanding of goal-oriented care.

The aim of this study is to address these knowledge gaps by means of a concept analysis to clarify the existing ambiguity and make an overview of the already existing knowledge. Clarity on the concept of goal-oriented care will enhance the understanding and will (potentially) facilitate the implementation of goal-oriented care interventions.

Method

This concept analysis aims to present an overview and synthetization of the existing literature regarding goal-oriented care for chronical ill patients in primary care. This will be performed by analyzing the concept into antecedents, attributes and consequences following the method of Walker and Avant (20). This method provides a framework of eight iterative steps: 1. select a concept, 2. determine the aims or purposes of analysis, 3. identify all concept definitions and select the literature, 4. determine different attributes, 5. identify a model case, 6. identify an additional case, 7. identify antecedents and consequences, and 8. define empirical referents (20). In this concept analysis the

attributes are the heart and will present the characteristics of goal-oriented care and allow the broadest insight into the concept (20).

Step 1: select a concept

Goal-oriented care has been defined as an underpinning strategy for primary care reform in Flanders, Belgium. The concept is presented as one of the main topics of 'The Primary Care Academy' (PCA). The PCA is a consortium consisting of four universities (Ghent University, University of Antwerp, Catholic university of Leuven, Vrije Universiteit of Brussels), six universities of applied sciences (UAC VIVES, UAC Artevelde, UAC Ghent, UAC Leuven-Limburg, UAC Karel de Grote, UAC Thomas More) and important stakeholders (Flemish Patient Platform and White-Yellow Cross; a home care organization) in Belgium with the aim to strengthen the primary care organization and delivery. The PCA includes experts in primary care from a variety of healthcare and welfare disciplines. Discussions in the research group working on goal-oriented care created a necessity to clarify the concept.

Step 2: determine the aims and purposes of the analysis

The aim of this concept analysis is to build a common understanding to eliminate ambiguity between the concepts related to goal-oriented care. Specifically, the scope of the concept analysis is to define goal-oriented care for people with chronic conditions at the level of primary care.

Step 3: select the literature

A preliminary combination of search terms was identified: 'goal-oriented care', 'chronic care' and 'primary care'. Based on these keywords a first search was performed to identify adjacent terms in the literature. The search strategy was revised in consultation with the librarian of the university and the senior researchers. The definitive keywords were: 'goal-oriented care', 'goal-oriented medical care', 'person-centered goal-setting', 'patient-centered goal-setting', 'goal-oriented patient care' and 'patient priorities', emphasized goal-oriented care and it synonyms. In a first phase, the keywords were

entered in PubMed, Embase and Cochrane Library (table 1). In a second phase, CINAHL, OTSeeker, PsycINFO and Web of Science were consulted and confirmed the first results.

**PubMed**<br>(goal-directed care[MeSH Terms]) OR goal-oriented care [Title/abstract]) OR goal-oriented medical care [Title/abstract]) OR person-centered goal-setting [Title/abstract]) OR patient centered goal-setting [Title/abstract]) OR goal-oriented patient care[Title/abstract]) OR patient priorities [Title/abstract])<br>**Embase**<br>**'goal-oriented care'**:ab,ti OR '**goal-oriented medical care'**:ab,ti OR '**person-centered goal-setting'**:ab,ti OR '**patient centered goal-setting'**:ab,ti OR **'goal-oriented patient care'**: ab,ti OR '**patient priorities'**:ab,ti

Table 1 Overview of the search strings

Articles resulting from this search were put in Rayyan (21) to administer the data. A first selection based on title and abstract was performed with regard to the predefined in- and exclusion criteria. Inclusion criteria: (a) goal-oriented care as a health-related concept, (b) mentioning goal-setting, goal-oriented care or related concept (e.g. person-centered integrated care) and (c) focusing on patients with a chronic condition or multimorbidity. Exclusion criteria: (a) focusing on single-disease management (b) goals regarding disease-specific outcomes (e.g. cancer or diabetes) and (c) focusing on goal-oriented care in a specific context (e.g. rehabilitation center). Articles resulting from this first search were subjected to a full text screening based on the initial criteria and: (a) full text available, (b) written in English, (c) referring to goal-oriented care or related concepts as a concept and (d) containing information of a theoretical building of a definition.

Step 4: defining the attributes

The determination of the attributes started with a discussion of four key articles (1, 6, 22, 23) selected by the first author based on the divers approaches of goal-oriented care. These key articles were analyzed, deconstructed into codes and discussed with the entire research group resulting in a first overview of attributes of goal-oriented care. In a second phase, new articles were added and analyzed until all relevant literature was included. The different codes were put into NVIVO12 to synthesize the data and to initiate further discussion with the research group. This resulted in the final attributes (table 3). The method starting from reading the first article to defining the attributes is characterized by an iterative process in which the attributes were reformulated until consensus was reached.

STEP 5: IDENTIFY A MODEL CASES, A CONTRARY CASE AND A BORDERLINE CASE

A model case is presented as a narrative of how goal-oriented care could be conceptualized and illustrates all defined attributes of goal-oriented care (20). A contrary and borderline case differ from this model case and do not include all of the attributes and/or differ in one of them.

Step 6: identify antecedents and consequences

Antecedents are events or incidents that precede the process of applying goal-oriented care. Consequences are those events or incidents as a result of applying goal-oriented care (20).
The antecedents and consequences were searched simultaneously with the attributes (step 4). Results have been discussed by the entire research group until consensus was reached.

Step 7: define empirical referents

Empirical referents provide an overview of the identified assessment tools related to the attributes aiming to make the concept, goal-oriented care, measurable. These assessment tools may be seen as

the underpinning needs and characteristics when developing an evaluation method of goal-oriented care.

Results

Step 1-3

A first search based on the predefined terms (Table 1) resulted in 590 articles; 82 from Cochrane Library, 188 from Embase and 313 from PubMed. After removing the duplicates, 366 articles were screened by title and abstract yielding 68 articles. A full text screening of these 68 articles lead to 15 articles that fitted the predefined in- and exclusion criteria (step 3). Based on the snowballing method of adding new articles based on references, citations and similar articles 22 additional articles were added. This resulted in a total of 37 articles (Figure 1) (Table 2) that were selected for the full text analysis.

Figure 1: Flow chart demonstrating the search string

Table 2 Overview of the selected articles

Papers identified based on full text screening					
No.	Year	Authors	Title	Study design	Journal
1	1991	Mold, Blake, Lorne, Becker (12)	Goal-oriented medical care.		Family Medicine
2	2011	De Maeseneer, Boeckxstaens (24)	Care for non-communicable diseases (NCD's): time for a paradigm-shift.		World Hospital and health services.
3	2012	Reuben, Tinetti (10)	Goal-oriented patient care- an alternative health outcomes paradigm.	Perspective	The New England journal of Medicine
4	2014	Bayliss, Bonds, Boyd, Davis, Finke, Fox, Stange (25)	Understanding the context of health for persons with multiple chronic conditions: moving from what is the matter to what matters.		Annals of Familiy Medicine
5	2014	Kramer, Bauer, Dicker, Durusu-Tanriover, Ferreira, Rigby, van Hulsteijn (8)	The changing face of internal medicine: patient- centered care.	Position paper	European Journal of Internal Medicine
6	2015	Bernsten, Gammon, Steinsbekk, Salamonsen, Foss, Ruland, Fonnebo (26)	How do we deal with multiple goals for care within an individual patient trajectory? A document content analysis of health service research papers on goals for care.	Document content analysis	BMJ Open
7	2016	Blom, Elzen, Houwelingen, Heijmans, Stijnen, Van Den Hout, Gussekloo (27)	Effectiveness and cost-effectiveness of a proactive, goal-oriented, integrated care model in general practice for older people. A cluster randomised controlled trial: integrated systematic care for older people-the ISCOPE study.	Cluster randomised controlled trial	Age and ageing

8	2016	Boeckxstaens, Willems, Lanssens, Decuypere, Brusselle, Kühlein, Sutter (28)	A qualitative interpretation of challenges associated with helping patients with multiple chronic diseases identify their goals.	Qualitative research	Journal of comorbidity
9	2016	Mangin, Stephen, Bismah, Risdon (29)	Making patient values visible in healthcare: a systematic review of tools to assess patient treatment priorities and preferences in the context of multimorbidity.	Systematic review	BMJ Open
10	2016	Schimdt, Babac, Pauer, Damm, von der Schulenberg (30)	Measuring patients priorities using the Analytic hierarchy process in comparison with best-worst scaling and rating cards: methodological aspects and ranking tasks.		Health economics review
11	2016	Tinetti, Esterson, Ferris, Posner, Blaum (1)	Patient priority decision making and care for older adults with multiple chronic conditions.		Clinical geriatric medicine
12	2018	Bernsten, Hoyem, Lettrem, Rul, Rumpsfeld, Gammon (6)	A person-centered integrated care quality framework, based on qualitative study of patient's evaluation of care in light of chronic care ideals.	Qualitative evaluative review	BMC Health Services Research
13	2019	Feder, Kiwak, Costello, Dindo, Hern, Bigos, Naik (3)	Perspective of patients in identifying their values-based health priorities.	Qualitative study	Journal of the American Geriatrics Society
14	2019	Franklinn, Lewis, Willis, Roger, Venville, Smith (31)	Controlled, constrained or flexible? How self-management goals are shaped by patient-provider interactions.	Conversation analysis	Qualitative health research
15	2019	Tinetti, Dindo, Smith, Blaum, Costello, Ouellet, Naik (32)	Challenges and strategies in patient's health priorities-aligned decision-making for older adults with multiple chronic conditions.	Participant observation qualitative study	PLOS One

Papers identified through snowballing					
No.	Year	Authors	Title	Study design	Journal
16	2006	Hurn, Kneebone, Cropley (33)	Goal setting as an outcome measure: a systematic review	Systematic review	Clinical Rehabilitation
17	2009	Bodenheimer, Handley (34)	Goal-setting for behavior change in primary care: an exploration and status report.	Status report	Patient education and counseling
18	2011	Junius-Walker, Stolberg, Steinke, Theile, Hummers-Pradier, Dierks (35)	Health and treatment priorities of older patients and their general practitioners: a cross-sectional study.	Qualitative study	BMC Geriatrics
19	2012	Rijken, Bekkema, Boeckxstaens, Schellevis, De Maeseneer, Groenewegen (2)	Chronic disease management programs: an adequate response to patients' needs?		Health Expectations
20	2014	Lenzen, Daniëls, van Bokhoven, der Weijden, Beurskens (36)	Setting goals in chronic care: shared decision making as self-management support by the family physician.	Background paper	European Journal of General Practice
21	2017	Kangovi, Mitra, Smith, Kulkarni, Turr, Huo, Glanz, Grande, Long (37)	Decision-making and goal-setting in chronic disease management: baseline findings of a randomized controlled trial.	Randomized controlled trial	Patient education and counseling
22	2017	Mold (38)	Goal-directed health care: redefining health and health care in the era of value-based care.		Cureus
23	2017	Steel Gray, Wodchis, Upshur, Cott, McKinstry, Mercer, Palen, Ramsay, Thavorn (39)	Supporting goal-oriented primary health care for seniors with complex care needs using mobile technology: evaluation and implementation of the health system performance research network, Bridgepoint electronic patient reported outcome tool.	Pragmatic cluster randomized controlled trial	JMIR Research Protocols

24	2017	Schellinger, Anderson, Frazer, Cain (40)	Patient self-defined goals: essentials of person-centered care for serious illness.	Descriptive qualitative analysis	American Journal of Hospice
25	2017	Vermunt, Harmsen, Elwyn, Westert, Burgers, Rikkert, Faber (41)	A three-goal model for patients with multimorbidity: a qualitative approach.	Qualitative study	Health Expectations
26	2017	Vermunt, Harmsen, Westert, Rikkert, Faber (13)	Collaborative goal setting with elderly patients with chronic disease or multimorbidity: a systematic review.	Systematic review	BMC Geriatrics
27	2018	Kessler, Walker, Sauvé-Schenk, Egan (23)	Goal setting dynamics that facilitate or impede a client-centered approach.	Conversational analysis	Scandinavian Journal of Occupational Therapy
28	2018	Naik, Dindo, Van Liew, Hundt, Vo, Hernandez-Bigos, Esterson, Geda, Rosen, Blaum, Tinetti (4)	Development of a clinically feasible process for identifying individual health priorities.	Prospective development and feasibility study	Journal of the American Geriatrics Society
29	2019	De Groot, Schönrock-Adema, Zwart, Damoiseaux, Jaarsma, Mol, Bombeke (42)	Learning from patients about patient-centredness: a realist review: BEME guide No.60	Realist review	Medical Teacher
30	2019	Kuluski, Guilcher (43)	Towards a person-centred learning health system: understanding value from the perspectives of patients and caregivers.		Healthcare papers
31	2019	Kuluski, Peckham, Gill, Gagnon, Wong-Cornall, McKillop, Parsons, Sheridan (9)	What is important to older people with multimorbidity and their caregivers? Identifying attributes of person centered care from the user perspective.	Qualitative descriptive study	International Journal of Integrated Care

32	2019	Reuben, Jennings (11)	Putting goal-oriented patient care into practice.		Journal of the American Geriatrics Society
33	2019	Salter, Shiner, Lenaghan, Murdoch, Ford, Winterburn, Steel (22)	Setting goals with patients living with multimorbidity: qualitative analysis of general practice consultations.	Qualitative analysis	British Journal of General Practice
34	2019	Tinetti, Naik, Dindo, Costello, Esterson, Geda, Rosen, Hernandez-Bigos, Smith, Ouellet, Kang, Lee, Blaum (44)	Association of patient priorities-aligned decision-making with patient outcomes and ambulatory health care burden among older adults with multiple chronic conditions.	Nonrandomized clinical trial	JAMA internal medicine
35	2020	Eckhoff, Weiss (45)	Goal-setting: a concept analysis	Concept analysis	Nursing Forum
36	2020	Purkaple, Nagyaldi, Todd, Mold (46)	Physician’s respone to patient’s quality-of-life goals.		Journal of the American Board of family Medicine
37	2020	Sathanpally, Sidhu, Fahami, Gillies, Kadam, Davies, Khunti, Seidu (47)	Priorities of patients with multimorbidity and of clinicians regarding treatment and health outcomes: a systematic mixed studies review.	Systematic review	BMJ Open

Step 4: attributes

The systematic analysis of the 37 selected papers could identify many different attributes of goal-oriented care. Synthesizing these attributes, goal-oriented care could be described as a multifaceted dynamic and iterative process of care (first main attribute) underpinned by patients' values (second main attribute). For the process of goal-oriented care 5 sub attributes and 7 descriptive items could be identified (table 3). These attributes interact and cannot be interpreted separately.

Table 3 Overview of attributes

1. Goal-oriented care is a multifaceted, dynamic and iterative process.	1.1 Goal-elicitation builds a patient-provider relationship.	
	1.2 Goal-oriented care entails goal-setting.	1.2.1Patient-provider interaction guides goal-setting.
		1.2.2 Patients' needs and preferences are the foundation of SMART formulated goals.
		1.2.3 Care plan is based on patients' needs and preferences.
		1.2.4 Care is delivered according to the care plan.
	1.3 Goal-evaluation is a reflexive process.	1.3.1 Feedback should be given to the goals.
		1.3.2 Evaluation entails questioning how goals are being met.
		1.3.3 Goals must be measurable.
2. Goal-oriented care embraces patients' values.	2.1 Goal-oriented care must be placed in patients' context.	
	2.2 Goal-oriented care must be tailored to patients' needs and preferences.	

Goal-oriented care is a multifaceted, dynamic and iterative process

The majority of the authors presented goal-oriented care as a stepwise approach (1, 3, 4, 6, 11-13, 22, 23, 34, 36, 39, 40, 48, 49). Even though every paper defined their own approach, overall three stages could be identified: (a) goal-elicitation (b) the actual stage of goal-setting and (c) a reflexive goal-evaluation stage. These three stages will be further discussed.

Bernsten et al. (6) emphasized the dynamic and iterative characteristics of the goal-oriented process of care. They described that goal-oriented care entails going back and forth between the three stages

(6). From this perspective, goals are not described as an endpoint, but they can be adjusted, discarded, modified or new goals might be set (11, 32). This will be further discussed in the stage of goal-evaluation.

Overall, in the goal-oriented process of care, the patient is described as an active partner (1). Therefore, a good communication in a continuous patient-provider relationship is described to be of utmost importance (40). In addition, goal-oriented care should be considered as care over time rather than a one-time intervention (50). In terms of outcomes, it is not entirely clear whether goal-oriented care should focus on (a) maintaining the status quo or (b) improving the patients' situation (11). Although there is consensus that the care process is oriented to the current needed care rather than care needed in the future (1).

Goal-elicitation builds a patient-provider relationship

As described earlier, the overall analysis could identify goal-elicitation as the first stage in the process of goal-oriented care. In this first stage, providers are presumed to offer time and space to patients to tell their stories in order to work towards the patients' agenda (23). Therefore, patients have to be ready and should be actively encouraged to tell their story. Tinetti and colleagues described this as 'the patient's state of readiness' (1). This first stage is considered to be essential to work towards a balanced patient-provider conversation and relation (46). Salter et al. described this stage as a shared process between patients and providers that reinforces and further builds their relationship (22). This specific part of the process of goal-oriented care is also described as a mean to achieve a greater level of shared understanding and mutual commitment between the patient and the provider (38). Specific attention to the stage of goal-elicitation is described to create a supportive context for effective goal-setting in the next stage (22).

Goal-oriented care entails goal-setting

Next to the goal-elicitation stage, the literature identifies a goal-setting stage. Franklin and colleagues analyzed patient-provider conversations during goal-setting and concluded that the goal-setting stage

serves as a mechanism to embrace patients' needs within the social context he lives in (31). When this process is done properly, goal-setting should support the patients to continue doing what matters most to them. This would help them to cope with their conditions (31). Within this process of goal-setting different sub attributes could be identified that are considered necessary for proper goal-setting.

Patient-provider interaction guides goal-setting

The patient-provider interaction is characterized by a patient-centered approach (22) in which goals are set in collaboration (41). Hereby, patients and providers agree on health-related goals (2, 11, 12, 34, 39, 41, 49, 51) and find common ground (50). Tinetti et al. described the importance of considering patients as active partners in the goal-setting process (32). Rijken et al. mentioned that patients' goals have to be discussed in a dynamic conversation continuously taking the patients' needs, preferences and abilities into account (2).

To facilitate a collaborative approach it is suggested that providers emphasize the patients' narratives reflecting their lived experience (38). Next to a collaborative approach, negotiation is important and considered inevitable (4, 6, 22, 36, 48). Lenzen et al. defined this as goal-negotiation, which involves discussion of any kind of problems, exploration of the patients' values, needs and capabilities and deliberation on patients' goals (36). In goal-negotiation, formulating and agreeing on a specific goal are important components (22).

Since the goal-setting process needs to be driven by patients' needs and preferences, there seems to be a general understanding to shift the focus from the provider to the patient (23). Different authors reported various strategies to facilitate this shift. Mold stated that the shift implies that prioritization of the individual health-related goals and the amount of effort in achieving them should be made by the individual (12). Naik et al. stated that patients are indeed encouraged to share their priorities, but adds that providers are encouraged to align their care with the patients' health priorities (4). More recent publications talking about goal-setting describe a circular and shared process aimed at

improving the balance and power differentials in the patient-provider relationship (4, 37). This balance can be improved by putting themselves in someone's shoes to understand the other's constraints (43).

Patients' needs and preferences are the foundation to set goals

One of the important challenges in our understanding of the concept of goal-oriented care is the lack of clear understanding on patient goals. Nearly all authors described that goals should be grounded on the patients' needs and preferences (1-4, 6, 22, 23, 31, 32, 37, 40, 46, 48, 50, 52, 53). It is described that goals should be based on the context, resources and capabilities of patients (46), that they should be approved by patients (6) and that they should foremost represent what the patients want and not necessarily what the providers want (11, 40). Other authors recommended the combination of patients' and providers' goals which could be related to the aspect of goal-negotiation (23, 43).

There also seems to be some confusion in the categorization of goals. Some authors emphasized that goals should contain core values of patients (e.g. the broader aspects that matter most to the patient) (1, 4) . These goals are named as 'overarching goals' (6, 11, 23, 40) leading to a broad description of the goal (e.g. I want to live in my own home as long as possible (1)) (6). Others argued that these overarching goals might not be easy to work with and describe that these goals should be broken down into sub goals (e.g. I want to walk 2 blocks without shortness of breath (1)) (6). Goals differ for each individual and will change over time (12). Aside from overarching goals and sub goals many of the authors mention the importance of setting SMART goals (1, 6, 22, 23, 34, 45, 48-50). A SMART goal is created when patients and providers collaborate to untangle the goal itself, the importance of that goal is emphasized to the patient, the perceived achievability of the goal is evaluated, as well as the timing of the goal and any supports and resources available (34). On the meta-perspective, overarching goals are too broad to make SMART (think about the grandmother aiming to get her grandchildren from school as long as possible). Therefore they should be divided in the sub-goals (in sub goals such as I need to be able to walk without being tired after 10 yards) that are specific enough to be measured.

In one of his first publications Mold brings in a specific discourse around the type of goals namely that goal-oriented care should assist patients in achieving their maximum individual health potential (12), hereby making the link with health. One should however notice that health should be described from the patients' perspective; as the ability to live his life, and not as the absence of disease (1, 12). Patients' goals are oriented towards health outcome goals. Patients hope to achieve these individual health outcomes through their health care (e.g. function, social activities and symptom relief)(1). Health outcome goals describe activities that promote change in physical and cognitive well-being or health (35). Naik et al. specifically relate patient goals to the care they are willing to receive and able to perform (4).

Care plan is based on patients' needs and preferences

Many authors relate goal-oriented care to the construction of a care plan based on the patients' needs and preferences and specifically mention that these care plans should reflect the patients' personal goals that have been identified in the previous stage (1-3, 6, 11, 25, 27). There is a consensus that the care plan should reflect the question: 'What matters to you?' (11, 32, 43, 48, 52). Strategies to achieve the patients' needs and preferences should be implemented in the care plan (12). Furthermore, Bernsten and colleagues stated that the care plan might also include an interprofessional review of the goals (6). Therefore, it is necessary to involve all providers and preferably patients' informal care giver and family in the whole process (3, 6, 13). An interprofessional review of the goals might benefit the coordination of the care plans between the different providers and facilitate integrated care delivery (1, 4, 29).

Care delivery according to the care plan

Patients and providers should implement the care plan and translate it into care delivery. Although, little is known about how care should be delivered, it is evident that it must be in accordance with the care plan that is set up in the previous stage (6). For this stage Tinetti et al. specifically mentioned to start the stage of care delivery by prioritizing on simple interventions in order to achieve one or more small goals to keep patients motivated (1). This simple interventions could focus on the sub-goals described in previous paragraphs to eventually work towards the overarching goals.

Goal-evaluation is a reflective process

The overall synthesis/analysis of the literature could identify goal-evaluation as the third and final stage in the process of goal-oriented care. For this stage authors described a dynamic and iterative process that allows reflection and feedback next to assessing whether and how goals have been met (32, 48). In this process goals can be redefined and adjusted. Possible reasons to adjust goals might be that goals have been too difficult to achieve or were no longer desired or relevant to the patients' situation (11). Although many authors acknowledge the possibility and importance of goal adjustment, there is also discussion that goal-oriented processes of care requires that goals can be measured (12). Steele Gray and colleagues described the importance of qualifying and quantifying the process proceeded to achieve the goals (39). In contrast, Salter and colleagues described that making the goals measurable could overcomplicate and distance the patient from their own goal and might therefore not be beneficial to the process of goal-oriented care (22).

Goal-oriented care embraces patients' values

In the previous attributes, goal-oriented care is described as a dynamic and iterative process in which two underpinning values are identified (4). Firstly, goal-oriented care must be placed in the patient's context and secondly, goal-oriented care must be tailored to the patient's needs and preferences.

Goal-oriented care must be placed in patients' context

The whole goal-oriented process of care starting from goal-elicitation to goal-evaluation needs to be placed in the patient's context. According to different authors this means that the process must be tailored to the patient's situation (3, 11, 36, 52). This does not only refer to the personal context, but also to the social and the cultural context. Therefore, this process is influenced by different contextual factors that should must be taken into account when developing the care plan (29, 36).

Goal-oriented care must be tailored to patients' needs and preferences

When reviewing the attributes, it is clear that patients' needs and preferences form the common thread. The question 'What is the matter with the patient' must be retranslated to 'What matters to the patient?' (1, 6, 22, 32). This question enables patients to tell their story and open up in which they are considered to reflect on their achievements and personal agenda (23). As a result, patients will have the feeling to be approached as a person instead of through their condition (6).

CASES

The method of Walker and Avant prescribes that several cases should be described to illustrate the attributes defined in step 4 (20). The first case of Joseph encompasses all the attributes identified in the literature and is therefore identified as a model case. It is a fictive example of delivering care according to the goal-oriented process of care with focus on the underpinning attributes. The second case of Ben is identified as an additional case since it lacks one or more of the attributes. E.g. in the case of Ben the stage of goal-evaluation is missing. This stage is needed to make adjustment and reflections according to the process of achieving the personal goals. Finally, the third case of Mary is an example of the opposite of goal-oriented care. This is described as a contrary case. In this case, the health care provider does not take the needs and preferences of Mary into account. The provider only thinks about convincing Mary of a healthy lifestyle which for her is not the main reason to visit her health care provider. Her main focus is on being able to go on a city trip to Madrid.

Joseph, 68- year old suffers from diabetes, hypertension and cardiovascular disease. Throughout his entire working life, he was a secondary school teacher. He has been retired for three years now. Despite the fact that he is limited by his health condition, he loves spending time with gardening and playing with his grandchildren.

A few years ago he was a passionate cyclist, but his racing bike has been stored for a long time now. His friends encourage him to cycle with them on a weekly base. His wife supports this initiative and argues that this will be beneficial for his social contact.

Every month Joseph visits his family doctor for a check-up. For each consultation, he prepares a list of things he wants to discuss. He has the chance to share his story in an open communication in which trust and mutual respect are key components.

In his monthly check-up with his family doctor he suggests his wishes to cycle again with his friends. His doctor doubts whether this will be possible and after discussion and negotiation, they pl[illegible]hat

Box 1 Model case of Joseph

Ben, a 30-year old man, was renovating a house that he bought with his girlfriend when he was diagnosed with MS. They made plans to marry next year and to make a world trip as honeymoon. These plans have been put aside due to the recent diagnosis. Although he was feeling down and did not have the energy to do anything he ended up with an excellent physician. Initiated by the interaction and the conversation with his physician he was enabled to set goals again and to look

Box 2 Additional case of Ben

Mary is a 40-year old mother of two children and dealing with obesity since her childhood. Due to her weight, she has a lot of joints pain and is short of breath which limits her exercising capacity. In the upcoming summer, she wants to make a city trip with the entire family to Madrid. Therefore she is seeing her physician to discuss the options to travel as painless and comfortable as possible. Her physician does not allow the travel plans and instructs her to first strive for a healthy weight and then plan trip when she has lost weight. This is not aligned with the wishes of Mary who only want's a short-term solution to cope with her condition during the city trip. In the end, she leaves the consultation room with a referral to a dietitian and sport coach.

Box 3 Contrary case of Mary

Antecedents

Antecedents are events or incidents that occur prior to the investigated concept. In this concept analysis, provider preparedness and patient preparedness are required to provide goal-oriented care.

In terms of provider preparedness many authors discussed the importance of training (6, 7, 23, 27, 31, 41, 49). Notwithstanding that several authors (1, 4, 13, 22, 27, 32, 37) mentioned the importance of trained health care providers, there was a difference in the training they received (supplementary file 2). Differences can be found in the target population reached with the training, both in monodisciplinary and interprofessional training (e.g. general practitioners (22), practice nurses (27), duration of the training (e.g. three hour (22), number of sessions (27)) and training method (e.g. role-play (32)) . Thereby, the content of the training was tailored to the skills needed to carry out the intervention correctly and differ therefore in each training.

A second aspect that is discussed concerning provider preparedness focused on the personal skills of providers (1, 6, 13, 22). These include communication and balancing skills in which an open communication with the patient is necessary and in which an equal balance between the patient and provider is a premise (1, 6, 13, 22). Other defined skills were the provider's ability to listen, understand and bearing witness to the patient's story (22) and their willingness to change and learn new skills to provide care according to the goal-oriented process of care (1).

Next to provider preparedness some authors (1, 11, 41) specifically talk about the need of patient preparedness. Patients needed to be prepared to share their needs and preferences when entering a care relationship (1). Some authors translate the importance of patient preparedness into patient education (1), others talked about patient guidance (11) or supporting patients in developing the skills to set personal goals (36).

Consequences

Consequences are those events or incidents that occur as a result of a concept. For the concept of goal-oriented care, the consequences defined throughout the papers could be categorized in: (a) patient-related consequences (1, 3, 4, 23, 29, 48), (b) provider-related consequences (1, 22, 29, 48), (c) care-related consequences (1, 22, 29) and (d) general consequences (4, 6, 29).

Patient-related consequences are the results for patients themselves after they received care following a goal-oriented process. A goal-directed approach could be expected to increase patient satisfaction, since the values, preferences, knowledge and opinions that each patient brought to the provider-patient relationship was more valued (38). Also, emphasis was put on the changed way of communicating in which patients felt more freely and able to speak (3). This led to the overall feeling of being heard, understood, respected and engaged in their care (29). Furthermore, a goal-oriented process of care could lead to a better understanding and more in-depth knowledge of patients regarding their health, activation of patients to be more involved in their care and an increase in their overall commitment. This resulted in the increase of adherence (3). Also Mold argued that it could contribute to a better adherence (12). In general, the gained in-depth knowledge of patients concerning their health and a better understanding of their tasks could help to improve their quality of life (3). This was enhanced by the maximization of function and the independency patients gained (12).

For providers, goal-oriented care assisted healthcare them in their decision-making (29) and gave them the opportunity to get to know their patients better. It enhanced patient-provider collaboration (12) and contributed therefore to more job satisfaction (22).

Care-related consequences were mainly focused on reducing costs, overtreatment and fragmentation (1, 22, 29), since care oriented to patients' priorities would reduce tests and treatments (44). Bernsten

et al. stated also that goal-oriented care could lead to an improvement of quality of care and quality of life (6).

Although, many positive outcomes have been presented, Reuben et al. mentioned a possible downside of goal-oriented care (10). They described that some decisions to strive for personal goals may worsen the providers' performanc[illegible]n aggregated health measures. For example, when a diabetic patient chooses to not follow his diet and keep on smoking, because it would be a too big lifestyle change, his HbA1c-level would not be aligned with the guidelines. Although, it could be a positive outcome from the patient perspective, it would influence the quality of care provided and the population health in a negative way.

Empirical referents

Empirical referents provide an overview of the identified assessments tools related to the attributes aiming to make the concept measurable.

None of the papers mentioned an empirical referent to measure the entire concept of goal-oriented care. Therefore, tools have been searched for each individual sub-attribute. Examples are listed in table 4 which gives an overview of possible tools and presents an example item presented in that tool. Listing the existing individual empirical referents might initiate the development of an overall empirical referent.

Table 4 Empirical referents

**Attribute**	**Example of item in the assessment tool**
Goal-elicitation	
Davis Observation Code (DOC) (54)	Discussing family, medical, or social history and/ or current family functioning.
Goal-setting	
Patient goal priority questionnaire (55)	Which activities are most important for you to manage?
Self-identified goals assessment (56)	Think about all of the things you want to be able to do. It might help to think about the things you did at<br>home before you went to the hospital, and things that are hard to do now. What types of things would you like<br>to work on or improve on in therapy before you go back home?
Goal-setting questionnaire	What are some specific goals you have in life?
C[illegible]M (57)	Semi-structured interview – discussing daily functioning and personal life.
Health outcome prioritization tool (58)	I would like to know how important ‘keeping you alive’, ‘maintaining independence’, ‘reducing or eliminating pain’ and ‘reducing or eliminating symptoms of dizziness, fatigue, shortness of breath’ is to you.
EPRO-tool (59)	Goal-setting for five different areas identified as most important.
Goal-evaluation	
Goal-attainment scale (60)	Determining goal-attainment using 5-point scale.
PACIC (61)	Asked to talk about my goals in caring for my condition.
Goal-setting evaluation tool (62)	Does the plan identify specific actions or activities that could help to reach the goal?
Person’s context and patient’s needs and preferences	
Person-centered primary care measure (PCPCM) (63)	My doctor or practice knows me as a person/ Over time, the practice helps me to meet my goals.
Patient centered observation form (64)	Collaborative upfront agenda setting.

CONCLUSION OF THE CONCEPT ANALYSIS

Figure 2 represents the overall synthesis of this concept analysis of goal-oriented care. Goal-oriented care could be described as a health care approach encompassing a multifaceted, dynamic and iterative process underpinned by the patient's context and values. The process is characterized by three stages: goal-elicitation, goal-setting and goal-evaluation in which patients' needs and preferences form the common thread. In order to be able to deliver care according to the principles of the goal-oriented care process, both providers and patients need to be prepared. In terms of the consequences of goal-oriented care literature points to the potential of goal-oriented care to improve patients' experiences and provider well-being, the potential to reduce costs and improve the overall health of the population. Furthermore, a model, a contrary and an additional case illustrated an example of goal-oriented care in practice. The empirical referents showed that it is currently not possible to measure goal-oriented care in its entirety and presented an overview of possible referents for each sub attribute. Although the literature allowed us to gain more insight into the concept of goal-oriented care, different aspects need to be further discussed.

Figure 2: Schematic representation of the antecedents, attributes and consequences

Discussion and conclusion

This concept analysis aimed to tackle the lack of a common understanding of goal-oriented care by identifying the attributes, antecedents and consequences using the method of Walker and Avant (20). The overall analysis showed that a goal-oriented care generally entails three stages. Despite these three stages the process of goal-oriented care cannot be implemented as a linear protocol or checklist. Two underpinning attributes, the patient's context and the patient's needs and preferences form the common thread throughout this goal-oriented process of care. These underpinning attributes represent the philosophy of care. Goal-oriented care is a continuous interaction where you go back and forth to gain a person-centered approach (Figure 2).

In the stage of goal-elicitation, greater consideration should be given to the patients' peripheral narrative reflecting their lived experiences (31). Several authors have investigated components of goal-elicitation. Murdoch and colleagues performed a conversation analysis of patients-providers interaction during their encounters and found that eliciting the patients' understanding is an important component (65). Ospina et al. investigated the extent to which patients' concerns are elicited across different clinical settings (66). They concluded that providers seldom elicit the patients' agenda. This reduces the chance that providers will orient their consultation towards the specific aspects that matter to the patient (66). One of the prerequisites to succeed in goal-elicitation is the mutual understanding about the expectations of the consultations between patients and providers and a qualitative relationship between patients and providers (65). The literature also mentions that patients need to have a set of skills to make appropriate health decisions and reflect on their health care choices (67). They have to be capable to open up and tell their story (68). It is important that patients understand the meaning of information communicated by the provider, must appreciate the consequences of the treatment options and must reason about the information based on his or her own values and preferences (68).

Next to the stage of goal-elicitation, the stage of goal-setting is defined. One of the remaining knowledge gaps is on what kind of goals patients set. Various work in different settings identified that patients do not necessarily have clearly defined goals for themselves (65). Although, several authors performed research on the categorization of patients' goals. Vermunt et al. performed for example a qualitative study to develop conceptual descriptions of goal-oriented care (41). They presented a three-level goal hierarchy containing disease- or symptom specific goals, functional goals and fundamental goals which provides more insight in the type of goals. A second example is the distinction made by Schellinger et al. between medical, nonmedical, multiple and global goals (40). Not only there is ambiguity on what goals patients set, it is also not clear how goals are being set. The systematic review of tools to assess patient treatment priorities and preferences by Mangin et al. found few relevant tools to set patient's goals (29). They argue for the need to develop specific strategies to make patient priorities visible in the clinical record and medical-decision making (29).

Goal-evaluation is pointed out as the last stage. As presented in the results, several authors described that goals should be made measurable for evaluation (22, 59). There are some pitfalls related to goal-evaluation. Salter et al. described that not all goals lend themselves to being measured (22). It is for example challenging to evaluate the goal 'I want to take my grandchildren from school as long as possible'. Another pitfall is that patients' goals would be simplified to what can be measured. Working towards goal-evaluation might increase the pressure on patients and providers to work in the same way as disease-specific guidelines do (69). Especially from the perspective of patients with multimorbidity it can be questioned whether disease-specific guidelines that are good for the disease are also good for the patient (69). Furthermore, evidence shows that older multimorbid patients place quantitative health outcomes, such as longer survival, on a lower level of importance (69). The focus must be on the patients' values and make healthcare more humane (38).

As mentioned for the antecedents it is important that patients and providers are prepared to work towards a goal-oriented process of care. The collaboration and co-creation between the two partners

and in an interprofessional team is an important but insufficient prerequisite to succeed in providing goal-oriented care. Currently patients are not stimulated to think about their care. They have to be stimulated to actively engage their narrative and to share their priorities. Also providers have to develop complementaty skills in which they learn to let go their own assumptions and solutions. They have to learn to integrate patients' narrative in their care plan and improve their communication skills to strengthen the mutual understanding between them (70). Voigt et al. observed that GPs are unaware of patients' priorities in daily life, which were in contrast with their perceived importance of patient's medical goals (70). Training and tools could provide the guidance needed to improve the communication(1, 4, 13, 22, 27, 32, 37). It could support providers in structuring the conversation, to set goals in collaboration with patients and to align their care to those goals. Not only does goal-oriented care offers a specific approach for one-on-one interaction between patients and providers, it could also facilitate interprofessional collaboration. It gives providers from divers disciplines the opportunity to deliver care following the same principles and to focus on pursuing patients' goals (34). Therefore training should also include the interprofessional perspective to facilitate a uniform attitude towards the patients' goals and principles of goal-oriented care in the entire team.

In terms of the consequences of goal-oriented care, limited studies have been able to demonstrate outcomes of goal-oriented care. Mostly positive outcomes have been presented towards the patients, providers, health system and overall population well-being. In that respect, goal-oriented care shows the potential to meet the components of the quadruple aim. It can be questioned if all providers experience increased satisfaction and well-being in providing goal-oriented care. Providers have to learn to cope with another way of delivering care. For example, a changed medication scheme as described in Josephs' case in order to work towards patients' goals. This goes against their basic principles to strive for the best possible health status including a comprehensive medication scheme. Besides that the provider well-being can be questioned, Blom et al. also contradicted the positive results for the health care system. They did not found a beneficial effect in health care use and costs when using a proactive, goal-oriented, integrated care model (27).

One of the reasons of the limited number of effectiveness studies of goal-oriented care is the lack of empirical referents. The concept must still undergo the transition towards an evaluable concept. Goal-oriented care is however identified by Etz and colleagues as one of the main constructs when developing a new comprehensive measure of high-value aspects of primary care, however they did not mention how it has to be done (71). Also Young et al. described outcome goals as a main construct when differentiating processes and outcomes for primary care and divided it further in goal-clarity for multimorbidity, goal-clarity for unique patient priorities and goal timing (72). It is clear that in order to gain more insight in the consequences of goal-oriented care further research must primarily focus on how goal-oriented care is provided and can be supported. In order to investigate the potential benefits of goal-oriented care, research also needs to work on developing indicators of the goal-oriented process of care.

Strengths, limitations and recommendations

The method of Walker and Avant provides a rigorous and systematic approach to refine the concept of goal-oriented care through the existing literature. A concept analysis is an exploration of an evolving concept which will need to be enriched by new knowledge. Therefore, it is influenced by contextual factors and must undergo adjustments to new implications and new insights based on further research. The iterative process of adding new articles following the snowballing method is one of the strengths compared to other types of reviews. In this concept analysis, this led to a larger number of articles than the original search. A possible explanation for this might be that goal-oriented care was covered by synonyms or similar concepts that were not covered by the original search. Despite the systematic approach, a concept analysis does not comprise a quality assessment of the literature. However, it seemed to be an appropriate method to provide the knowledge needed to understand the different components of goal-oriented care in its entirety.

The literature search identified both original research papers and position papers. Some original research papers (3, 4, 22, 39, 40) evaluated goal-oriented care in clinical practice. These papers

identified and described goal-oriented care as a stepwise intervention. Position papers (1, 11, 12, 34, 36) mostly described components of goal-oriented care rather than such a stepwise approach. The combination of both types gave more insight in the broad components of goal-oriented care.

This concept analysis could also be considered as a preliminary step to facilitate further research. One of the knowledge gaps revealed in this concept analysis is the lack of knowledge on what patients' goals are set, how goal-oriented care is delivered and how it is best put into practice in both one-on-one interactions between patients and providers and in interprofessional collaboration. In addition, the list of empirical referents made clear that a golden standard to evaluate goal-oriented care is missing. Initiating the development of an evaluation method could enable future intervention studies to gain more insight in the consequences of goal-oriented care and to make results comparable. This might be required to convince providers and policy makers of the benefits of goal-oriented care.

Goal-oriented care shows the potential to be a way forward for patients with chronic conditions and multimorbidity. However, further research is needed to further translate the current knowledge on the concept of goal-oriented care into a tangible workflow process of care that entails the three stages. This workflow should include the skills and tools patients and providers need to implement goal-oriented care in practice.

Conclusion

This concept analysis aimed to translate the concept of goal-oriented care into a common understanding so providers can better understand and use this concept in clinical practice. The various literature on goal-oriented care, based on position and original research papers, showed a stepwise approach of three stages. Overall, the underpinning attributes of patients' context and patients' values form a philosophy of care to which the process must be reflected. Furthermore, both patients and the providers need to develop new skills in order to rethink the way care is provided. Patients must therefore be enabled to open up and reflect on their own agenda. Providers instead must learn to let

go their own assumptions and solutions and communicate with their patients in a more balanced context. Based on the literature goal-oriented care shows the potential to improve patients' experience by listening to their needs and preferences, improve providers' well-being by the feeling of more satisfaction and reduce health care costs. Goal-oriented care could answer the challenges patients face with multiple care processes by initiating interprofessional collaboration. However, further research must focus on what and how goals are set, the translation of these findings into a workflow and must initiate the development of an evaluation method in order to investigate the effects of goal-oriented care processes on patients, providers and the health care system.

Acknowledgments

We are grateful for the partnership with the Primary Care Academy (academie-eerstelijn.be) and want to thank the King Baudouin Foundation for the opportunity they offer us for conducting research and have impact on the primary care of Flanders, Belgium.

References

1. Tinetti ME, Esterson J, Ferris R, Posner P, Blaum CS. Patient Priority-Directed Decision Making and Care for Older Adults with Multiple Chronic Conditions. Clinics in geriatric medicine. 2016;32(2):261-75.
2. Rijken M, Bekkema N, Boeckxstaens P, Schellevis FG, De Maeseneer JM, Groenewegen PP. Chronic Disease Management Programmes: an adequate response to patients' needs? Health Expect. 2014;17(5):608-21.
3. Feder SL, Kiwak E, Costello D, Dindo L, Hern, ez-Bigos K, et al. Perspectives of Patients in Identifying Their Values-Based Health Priorities. Journal of the American Geriatrics Society. 2019 67(7):1379-85.
4. Naik AD, Dindo LN, Van Liew JR, Hundt NE, Vo L, Hern, et al. Development of a Clinically Feasible Process for Identifying Individual Health Priorities. Journal of the American Geriatrics Society. 2018;66(10):1872-9.
5. Cassell A, Edwards D, Harshfield A, Rhodes K, Brimicombe J, Payne R, et al. The epidemiology of multimorbidity in primary care: a retrospective cohort study. Br J Gen Pract. 2018;68(669):e245-e51.
6. Berntsen G, Hoyem A, Lettrem I, Ruland C, Rumpsfeld M, Gammon D. A person-centered integrated care quality framework, based on a qualitative study of patients' evaluation of care in light of chronic care ideals. BMC Health Serv Res. 2018;18(1):479.
7. Ploeg J, Matthew-Maich N, Fraser K, Dufour S, McAiney C, Kaasalainen S, et al. Managing multiple chronic conditions in the community: a Canadian qualitative study of the experiences of older adults, family caregivers and healthcare providers. BMC Geriatr. 2017;17(1):40.
8. Kramer MH, Bauer W, Dicker D, Durusu-Tanriover M, Ferreira F, Rigby SP, et al. The changing face of internal medicine: patient centred care. European journal of internal medicine.25(2):125-7.
9. Kuluski K, Peckham A, Gill A, Gagnon D, Wong-Cornall C, McKillop A, et al. What is Important to Older People with Multimorbidity and Their Caregivers? Identifying Attributes of Person Centered Care from the User Perspective. International journal of integrated care. 2019;19(3):4.
10. Reuben DB, Tinetti ME. Goal-oriented patient care--an alternative health outcomes paradigm. N Engl J Med. 2012;366(9):777-9.
11. Reuben DB, Jennings LA. Putting Goal-Oriented Patient Care Into Practice. Journal of the American Geriatrics Society. 2019;67(7):1342-4.
12. Mold JW, Blake GH, Becker LA. Goal-oriented medical care. Family medicine. 1991;23(1):46-51.
13. Vermunt N, Harmsen M, Westert GP, Olde Rikkert MGM, Faber MJ. Collaborative goal setting with elderly patients with chronic disease or multimorbidity: a systematic review. BMC Geriatr. 2017;17(1):167.
14. Hobbs FR, Bankhead C, Mukhtar T, Stevens S, Perera-Salazar R, Holt T, et al. Clinical workload in UK primary care: a retrospective analysis of 100 million consultations in England, 2007–14. The Lancet. 2016;387(10035):2323-30.
15. IoMDoHCSCotFoP C, Donaldson M, Yordy K, Vanselow N. Defining primary care: an interim report. National Academy Press; 1994.
16. Ellner AL, Phillips RS. The Coming Primary Care Revolution. J Gen Intern Med. 2017;32(4):380-6.
17. Poitras ME, Maltais ME, Bestard-Denomme L, Stewart M, Fortin M. What are the effective elements in patient-centered and multimorbidity care? A scoping review. BMC Health Serv Res. 2018;18(1):446.
18. Elwyn G, Durand MA, Song J, Aarts J, Barr PJ, Berger Z, et al. A three-talk model for shared decision making: multistage consultation process. Bmj. 2017;359:j4891.

19. Lusk JM, Fater K. A concept analysis of patient-centered care. Nurs Forum. 2013;48(2):89-98.
20. Walker LOA, K.C. Strategies for theory constructing in nursing. Texas2019.
21. Mourad Ouzzani HH, Zbys Fedorowicz, and Ahmed Elmagarmid. Rayyan - a web and mobile app for systematic reviews. . Systematic Reviews. 2016;5(210).
22. Salter C, Shiner A, Lenaghan E, Murdoch J, Ford JA, Winterburn S, et al. Setting goals with patients living with multimorbidity: qualitative analysis of general practice consultations. Br J Gen Pract. 2019;69(684):e479-e88.
23. Kessler D, Walker I, Sauve-Schenk K, Egan M. Goal setting dynamics that facilitate or impede a client-centered approach. Scandinavian journal of occupational therapy. 2018;26(5):315-24.
24. De Maeseneer J, Boeckxstaens P. Care for noncommunicable diseases (NCDs): time for a paradigm-shift. World hospitals and health services : the official journal of the International Hospital Federation. 2011;47(4):30-3.
25. Bayliss EA, Bonds DE, Boyd CM, Davis MM, Finke B, Fox MH, et al. Understanding the context of health for persons with multiple chronic conditions: moving from what is the matter to what matters. Ann Fam Med. 2014;12(3):260-9.
26. Berntsen GK, Gammon D, Steinsbekk A, Salamonsen A, Foss N, Ruland C, et al. How do we deal with multiple goals for care within an individual patient trajectory? A document content analysis of health service research papers on goals for care. BMJ open. 2015;5(12):e009403.
27. Blom J, Elzen WD, Houwelingen Anne HV, Heijmans M, Stijnen T, Van Den Hout W, et al. Effectiveness and cost-effectiveness of a proactive, goal-oriented, integrated care model in general practice for older people. A cluster randomised controlled trial: Integrated systematic care for older people-the ISCOPE study. Age and Ageing. 2016;45(1):30-41.
28. Boeckxstaens, Willems, Lanssens, Decuypere, Brusselle, Kühlein, et al. A qualitative interpretation of challenges associated with helping patients with multiple chronic diseases identify their goals. Journal of comorbidity. 2016;6(2):120-6.
29. Mangin D, Stephen G, Bismah V, Risdon C. Making patient values visible in healthcare: a systematic review of tools to assess patient treatment priorities and preferences in the context of multimorbidity. BMJ Open. 2016;6(6):e010903.
30. Schmidt K, Babac A, Pauer F, Damm K, von der Schulenburg JM. Measuring patients' priorities using the Analytic Hierarchy Process in comparison with Best-Worst-Scaling and rating cards: methodological aspects and ranking tasks. Health economics review. 2016;6(1):50.
31. Franklin M, Lewis S, Willis K, Rogers A, Venville A, Smith L. Controlled, Constrained, or Flexible? How Self-Management Goals Are Shaped By Patient-Provider Interactions. Qualitative health research. 2019:1049732318774324.
32. Tinetti M, Dindo L, Smith CD, Blaum C, Costello D, Ouellet G, et al. Challenges and strategies in patients' health priorities-aligned decision-making for older adults with multiple chronic conditions. PloS one. 2019;14(6):e0218249.
33. Hurn J, Kneebone I, Cropley M. Goal setting as an outcome measure: A systematic review. Clinical rehabilitation. 2006;20(9):756-72.
34. Bodenheimer T, Handley MA. Goal-setting for behavior change in primary care: an exploration and status report. Patient Educ Couns. 2009;76(2):174-80.
35. Junius-Walker U, Stolberg D, Steinke P, Theile G, Hummers-Pradier E, Dierks M-L. Health and treatment priorities of older patients and their general practitioners: a cross-sectional study. Quality in primary care. 2011;19(2).
36. Lenzen SA, Daniels R, van Bokhoven MA, van der Weijden T, Beurskens A. Setting goals in chronic care: Shared decision making as self-management support by the family physician. Eur J Gen Pract. 2015;21(2):138-44.
37. Kangovi S, Mitra N, Smith RA, Kulkarni R, Turr L, Huo H, et al. Decision-making and goal-setting in chronic disease management: Baseline findings of a randomized controlled trial. Patient Educ Couns. 2017;100(3):449-55.
38. Mold. Goal-directed health care: redefining health and health care in the era of value-based care. Cureus 2017;9(2).

39. Steele Gray C, Wodchis WP, Upshur R, Cott C, McKinstry B, Mercer S, et al. Supporting Goal-Oriented Primary Health Care for Seniors with Complex Care Needs Using Mobile Technology: Evaluation and Implementation of the Health System Performance Research Network, Bridgepoint Electronic Patient Reported Outcome Tool. JMIR research protocols. 2016;5(2):e126.
40. Schellinger SE, Anderson EW, Frazer MS, Cain CL. Patient Self-Defined Goals: Essentials of Person-Centered Care for Serious Illness. The American journal of hospice & palliative care. 2018;35(1):159-65.
41. Vermunt NP, Harmsen M, Elwyn G, Westert GP, Burgers JS, Olde Rikkert MG, et al. A three-goal model for patients with multimorbidity: A qualitative approach. Health expectations : an international journal of public participation in health care and health policy. 2018;21(2):528-38.
42. de Groot E, Schonrock-Adema J, Zwart D, Damoiseaux R, Van den Bogerd K, Diemers A, et al. Learning from patients about patient-centredness: A realist review: BEME Guide No. 60. Medical teacher. 2019:1-13.
43. Kuluski K, Guilcher SJT. Toward a Person-Centred Learning Health System: Understanding Value from the Perspectives of Patients and Caregivers. HealthcarePapers. 2019;18(4):36-46.
44. Tinetti ME, Naik AD, Dindo L, Costello DM, Esterson J, Geda M, et al. Association of Patient Priorities-Aligned Decision-Making with Patient Outcomes and Ambulatory Health Care Burden among Older Adults with Multiple Chronic Conditions: A Nonrandomized Clinical Trial. JAMA Intern Med. 2019.
45. Eckhoff DO, Weiss J. Goal setting: A concept analysis. Nursing forum. 2020.
46. Purkaple BA, Nagykaldi ZJ, Allahyar A, Todd R, Mold JW. Physicians' Response to Patients' Quality-of-Life Goals. The Journal of the American Board of Family Medicine. 2020;33(1):71.
47. Sathanapally H, Sidhu M, Fahami R, Gillies C, Kadam U, Davies MJ, et al. Priorities of patients with multimorbidity and of clinicians regarding treatment and health outcomes: a systematic mixed studies review. BMJ Open. 2020;10(2):e033445.
48. Reuben DB, Tinetti ME. Goal-oriented patient care - An alternative health outcomes paradigm. New England Journal of Medicine. 2012;366(9):777-9.
49. Heisler M, Bouknight RR, Hayward RA, Smith DM, Kerr EA. The relative importance of physician communication, participatory decision making, and patient understanding in diabetes self-management. Journal of general internal medicine. 2002;17(4):243-52.
50. Mangin D, Stephen G, Bismah V, Risdon C. Making patient values visible in healthcare: A systematic review of tools to assess patient treatment priorities and preferences in the context of multimorbidity. BMJ Open. 2016;6(6).
51. Vermunt NP, Harmsen M, Westert GP, Rikkert MGO, Faber MJ. Collaborative goal setting with elderly patients with chronic disease or multimorbidity: a systematic review. BMC geriatrics. 2017;17(1):167.
52. Boyd CM, Wolff JL, Giovannetti E, Reider L, Weiss C, Xue Q-l, et al. Health care task difficulty among older adults with multimorbidity. Medical care. 2014;52(0 3):S118.
53. Locke EA, Latham GP. A theory of goal setting & task performance: Prentice-Hall, Inc; 1990.
54. Callahan EJ, Bertakis KD. Development and validation of the Davis Observation Code. Fam Med. 1991;23(1):19-24.
55. Åsenlöf P, Siljebäck K. The patient goal priority questionnaire is moderately reproducible in people with persistent musculoskeletal pain. Physical therapy. 2009;89(11):1226-34.
56. Melville LL, Baltic TA, Bettcher TW, Nelson DL. Patients' perspectives on the self-identified goals assessment. Am J Occup Ther. 2002;56(6):650-9.
57. Dedding C, Cardol M, Eyssen IC, Dekker J, Beelen A. Validity of the Canadian Occupational Performance Measure: a client-centred outcome measurement. Clinical rehabilitation. 2004;18(6):660-7.
58. Fried TR, Tinetti M, Agostini J, Iannone L, Towle V. Health outcome prioritization to elicit preferences of older persons with multiple health conditions. Patient Educ Couns. 2011;83(2):278-82.

59. Steele Gray C, Gill A, Khan AI, Hans PK, Kuluski K, Cott C. The Electronic Patient Reported Outcome Tool: Testing Usability and Feasibility of a Mobile App and Portal to Support Care for Patients With Complex Chronic Disease and Disability in Primary Care Settings. JMIR Mhealth Uhealth. 2016;4(2):e58.
60. Toto PE, Skidmore ER, Terhorst L, Rosen J, Weiner DK. Goal Attainment Scaling (GAS) in geriatric primary care: a feasibility study. Arch Gerontol Geriatr. 2015;60(1):16-21.
61. Glasgow RE, Wagner EH, Schaefer J, Mahoney LD, Reid RJ, Greene SM. Development and validation of the Patient Assessment of Chronic Illness Care (PACIC). Med Care. 2005;43(5):436-44.
62. Teal CR, Haidet P, Balasubramanyam AS, Rodriguez E, Naik AD. Measuring the quality of patients' goals and action plans: development and validation of a novel tool. BMC medical informatics and decision making. 2012;12:152.
63. Etz RS, Zyzanski SJ, Gonzalez MM, Reves SR, O'Neal JP, Stange KC. A New Comprehensive Measure of High-Value Aspects of Primary Care. Annals of family medicine. 2019;17(3):221-30.
64. Adam P, Murphy CF, Dierich M, Hager KD. Seven Years of Teaching Communication With the Patient-Centered Observation Form. Fam Med. 2018;50(2):132-7.
65. Murdoch J, Salter C, Ford J, Lenaghan E, Shiner A, Steel N. The "unknown territory" of goal-setting: Negotiating a novel interactional activity within primary care doctor-patient consultations for patients with multiple chronic conditions. Soc Sci Med. 2020;256:113040.
66. Singh Ospina N, Phillips KA, Rodriguez-Gutierrez R, Castaneda-Guarderas A, Gionfriddo MR, Branda ME, et al. Eliciting the Patient's Agenda- Secondary Analysis of Recorded Clinical Encounters. J Gen Intern Med. 2019;34(1):36-40.
67. Hersh L, Salzman B, Snyderman D. Health Literacy in Primary Care Practice. Am Fam Physician. 2015;92(2):118-24.
68. Sine DM, Sharpe VA. Ethics, risk, and patient-centered care: how collaboration between clinical ethicists and risk management leads to respectful patient care. J Healthc Risk Manag. 2011;31(1):32-7.
69. Tinetti ME, Bogardus Jr ST, Agostini JV. Potential pitfalls of disease-specific guidelines for patients with multiple conditions. N Engl j Med. 2004;351(27):2870-4.
70. Voigt I, Wrede J, Diederichs-Egidi H, Dierks ML, Junius-Walker U. Priority setting in general practice: health priorities of older patients differ from treatment priorities of their physicians. Croatian medical journal. 2010;51(6):483-92.
71. Etz R, Stange KC. Measuring what matters in primary care. Global Advances in Health and Medicine. 2018;7:263.
72. Young RA, Roberts RG, Holden RJ. The Challenges of Measuring, Improving, and Reporting Quality in Primary Care. Ann Fam Med. 2017;15(2):175-82.
73. Lenzen SA, Daniels R, van Bokhoven MA, van der Weijden T, Beurskens A. Development of a conversation approach for practice nurses aimed at making shared decisions on goals and action plans with primary care patients. BMC Health Serv Res. 2018;18(1):891.

Supporting information

S1 Table 1. Overview preliminary version attributes.

S2. Table 2. Overview of training

Figure 1 Click here to access/download;Figure;Figure 1.tif

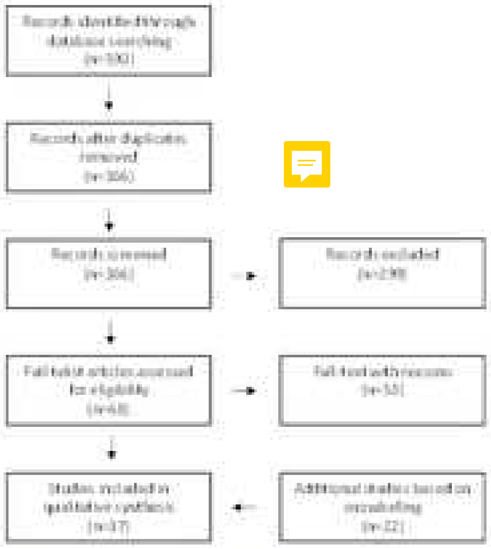

Figure 1 Flow chart demonstrating the search string

Figure 2

Click here to access/download;Figure;figure 2.tif

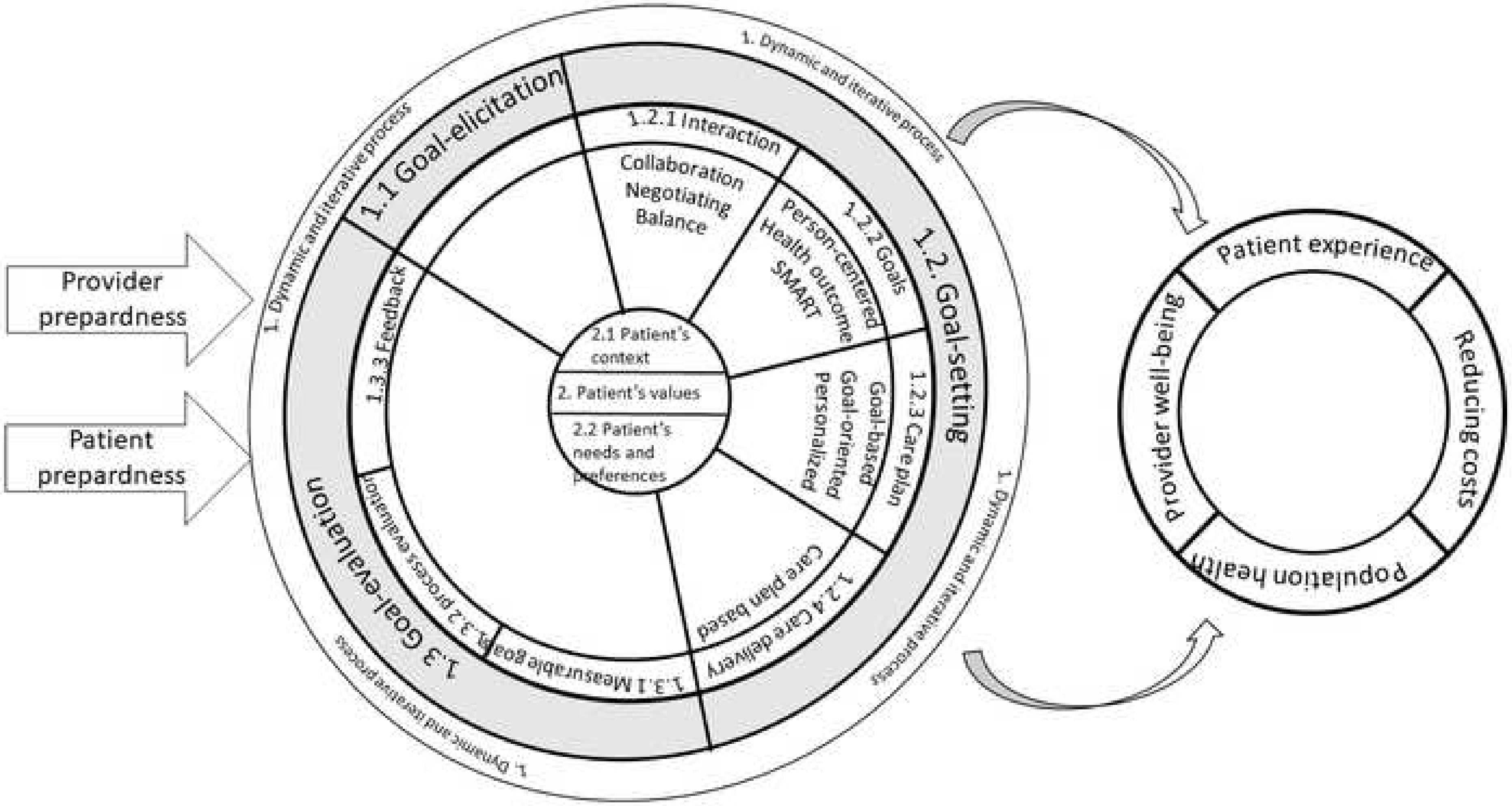

Supporting Information 1

Click here to access/download
Supporting Information
S1_File.pdf

Supporting Information 2

Click here to access/download
Supporting Information
S2_File.pdf
