# Supplementary material for: Goal-oriented care for patients with chronic conditions or multimorbidity in primary care: A scoping review and concept analysis
Source: PLoS One. 2022 Feb 4;17(2):e0262843. doi: 10.1371/journal.pone.0262843 (PMC8815876; doi:10.1371/journal.pone.0262843)
Supplement: Attachment — Submitted filename: PONE-D-20-30664_R1.pdf [file pone.0262843.s006.pdf]

PLOS ONE

Goal-oriented care for patients with chronic conditions or multimorbidity in primary care: a scoping review and concept analysis

--Manuscript Draft--

**Manuscript Number:**	PONE-D-20-30664R1
**Article Type:**	Research Article
**Full Title:**	Goal-oriented care for patients with chronic conditions or multimorbidity in primary care: a scoping review and concept analysis
**Short Title:**	Goal-oriented care in primary care
**Corresponding Author:**	Dagje Boeykens<br>Universiteit Gent Faculteit Geneeskunde en Gezondheidswetenschappen<br>Ghent, BELGIUM
**Keywords:**	goal-oriented care, goal-setting, patient-centeredness, chronic conditions, multimorbidity, review, concept analysis
**Abstract:**	Background<br>The healthcare system is faced by an ageing population, increase in chronic conditions and multimorbidity. Multimorbid patients are faced with multiple parallel care processes leading to a risk of fragmented care. These problems relate to the disease-oriented paradigm. In this paradigm the treatment goals can be in contrast with what patients value.<br>The concept of goal-oriented care is proposed as an alternative way of providing care as meeting patients' goals could have potential benefits. Though, there is a need to translate this concept into tangible knowledge so providers can better understand and use the concept in clinical practice. The aim of this study is to address this need by means of a concept analysis.<br>Method<br><br>This concept analysis using the method of Walker and Avant is based on a literature search in PubMed, Embase, Cochrane Library, PsychInfo, CINAHL, OTSeeker and Web of Science. The method provides eight iterative steps: select a concept, determine purpose, determine defining attributes, identify model case, identify additional case, identify antecedents and consequences and define empirical referents.<br><br>Results<br><br>The analysis of 37 articles revealed that goal-oriented care is a dynamic and iterative process of three stages: goal-elicitation, goal-setting, and goal-evaluation. The process is underpinned by the patient's context and values. Provider and patient preparedness are required to provide goal-oriented care. Goal-oriented care has the potential to improve patients' experiences and providers' well-being, to reduce costs, and improve the overall population health. The challenge is to identify empirical referents to evaluate the process of goal-oriented care.<br>Conclusion<br><br>A common understanding of goal-oriented care is presented. Further research should focus on how and what goals are set by the patient, how this knowledge could be translated into a tangible workflow and should support the development of a strategy to evaluate the goal-oriented process of care.
**Order of Authors:**	Dagje Boeykens
	Pauline Boeckxstaens
	An De Sutter
	Lies Lahousse
	Peter Pype
	Patricia De Vriendt

Powered by Editorial Manager® and ProduXion Manager® from Aries Systems Corporation

	Dominique Van de Velde
**Response to Reviewers:**	Dear editor<br>Dear reviewers<br><br>We appreciate your extensive and constructive feedback on our manuscript, it added a lot of value.<br>We aimed to respond to all of your remarks by point to point answers and changes in the manuscript. Please, find the changes in the 'response to the reviewers letter'.<br>Hopefully they will meet you expectations, otherwise we will make further adjustments.<br><br>Sincerely
**Additional Information:**	
**Question**	**Response**
**Financial Disclosure**<br><br>Enter a financial disclosure statement that describes the sources of funding for the work included in this submission. Review the submission guidelines for detailed requirements. View published research articles from *PLOS ONE* for specific examples.<br><br>This statement is required for submission and **will appear in the published article** if the submission is accepted. Please make sure it is accurate.<br><br>**Unfunded studies**<br>Enter: *The author(s) received no specific funding for this work.*<br><br>**Funded studies**<br>Enter a statement with the following details:<br>• Initials of the authors who received each award<br>• Grant numbers awarded to each author<br>• The full name of each funder<br>• URL of each funder website<br>• Did the sponsors or funders play any role in the study design, data collection and analysis, decision to publish, or preparation of the manuscript?<br>• **NO** - Include this sentence at the end of your statement: *The funders had no role in study design, data collection and analysis, decision to publish, or preparation of the manuscript.*<br>• **YES** - Specify the role(s) played.<br><br>* typeset	D.B. is a PhD student payed by the King Baudouin Foundation. Grant number: 2019-J5170820-211588 - King Baudouin Foundation - https://www.kbs-frb.be/nl/ - The funders had no role in study design, data collection and analysis, decision to publish, or preparation of the manuscript
**Competing Interests**	The authors have declared that no competing interests exist.

Powered by Editorial Manager® and ProduXion Manager® from Aries Systems Corporation

Use the instructions below to enter a competing interest statement for this submission. On behalf of all authors, disclose any competing interests that could be perceived to bias this work—acknowledging all financial support and any other relevant financial or non-financial competing interests.<br><br>This statement **will appear in the published article** if the submission is accepted. Please make sure it is accurate. View published research articles from *PLOS ONE* for specific examples.<br><br>**NO authors have competing interests**<br><br>Enter: *The authors have declared that no competing interests exist.*<br><br>**Authors with competing interests**<br><br>Enter competing interest details beginning with this statement:<br><br>*I have read the journal's policy and the authors of this manuscript have the following competing interests: [insert competing interests here]*<br><br>* typeset	
**Ethics Statement**<br><br>Enter an ethics statement for this submission. This statement is required if the study involved:<br><br>• Human participants<br>• Human specimens or tissue<br>• Vertebrate animals or cephalopods<br>• Vertebrate embryos or tissues<br>• Field research<br><br>Write "N/A" if the submission does not require an ethics statement.<br><br>General guidance is provided below. Consult the submission guidelines for	N/A

detailed instructions. **Make sure that all information entered here is included in the Methods section of the manuscript.**<br><br>**Format for specific study types**<br><br>**Human Subject Research (involving human participants and/or tissue)**<br>• Give the name of the institutional review board or ethics committee that approved the study<br>• Include the approval number and/or a statement indicating approval of this research<br>• Indicate the form of consent obtained (written/oral) or the reason that consent was not obtained (e.g. the data were analyzed anonymously)<br><br>**Animal Research (involving vertebrate animals, embryos or tissues)**<br>• Provide the name of the Institutional Animal Care and Use Committee (IACUC) or other relevant ethics board that reviewed the study protocol, and indicate whether they approved this research or granted a formal waiver of ethical approval<br>• Include an approval number if one was obtained<br>• If the study involved *non-human primates*, add *additional details* about animal welfare and steps taken to ameliorate suffering<br>• If anesthesia, euthanasia, or any kind of animal sacrifice is part of the study, include briefly which substances and/or methods were applied<br><br>**Field Research**<br><br>Include the following details if this study involves the collection of plant, animal, or other materials from a natural setting:<br>• Field permit number<br>• Name of the institution or relevant body that granted permission	
**Data Availability**<br><br>Authors are required to make all data underlying the findings described fully available, without restriction, and from the time of publication. PLOS allows rare	Yes - all data are fully available without restriction

exceptions to address legal and ethical concerns. See the PLOS Data Policy and FAQ for detailed information.<br><br>A Data Availability Statement describing where the data can be found is required at submission. Your answers to this question constitute the Data Availability Statement and **will be published in the article**, if accepted.<br><br>**Important:** Stating 'data available on request from the author' is not sufficient. If your data are only available upon request, select 'No' for the first question and explain your exceptional situation in the text box.<br><br>Do the authors confirm that all data underlying the findings described in their manuscript are fully available without restriction?	
**Describe where the data may be found in full sentences. If you are copying our sample text, replace any instances of XXX with the appropriate details.**<br><br>• If the data are **held or will be held in a public repository**, include URLs, accession numbers or DOIs. If this information will only be available after acceptance, indicate this by ticking the box below. For example: *All XXX files are available from the XXX database (accession number(s) XXX, XXX.*).<br>• If the data are all contained **within the manuscript and/or Supporting Information files**, enter the following: *All relevant data are within the manuscript and its Supporting Information files.*<br>• If neither of these applies but you are able to provide **details of access elsewhere**, with or without limitations, please do so. For example:<br><br>*Data cannot be shared publicly because of [XXX]. Data are available from the XXX Institutional Data Access / Ethics Committee (contact via XXX) for researchers who meet the criteria for*	All relevant data are within the manuscript and its Supporting Information Files

access to confidential data.<br><br>*The data underlying the results presented in the study are available from (include the name of the third party and contact information or URL).*<br>• This text is appropriate if the data are owned by a third party and authors do not have permission to share the data.<br><br>* typeset	
Additional data availability information:	

Powered by Editorial Manager® and ProduXion Manager® from Aries Systems Corporation

Manuscript Click here to access/download;Manuscript;Manuscript.docx

Goal-oriented care for patients with chronic conditions or multimorbidity in primary care: a scoping review and concept analysis.

Dagje Boeykens [12¶*], Pauline Boeckxstaens [2¶], An De Sutter [2], Lies Lahousse [3], Peter Pype [245], Patricia De Vriendt [156&], Dominique Van de Velde[15&], on behalf of the Primary Care Academy[^].

1. Department of Rehabilitation Sciences, Occupational Therapy. Faculty of Medicine and Health Sciences. Ghent University. Ghent. Belgium.
2. Department of Public Health and Primary Care, Faculty of Medicine and Health Sciences. Ghent University. Ghent. Belgium.
3. Department of Bioanalysis, Faculty of Pharmaceutical Sciences, Ghent University. Ghent. Belgium.
4. End-of-Life Care Research Group, Faculty of Medicine and Health Sciences. Vrije Universiteit Brussel and Ghent University. Ghent. Belgium.
5. Department of Occupational Therapy. Artevelde University College. Ghent, Belgium.
6. Frailty in Ageing (FRIA) Research Group, Department of Gerontology and Mental Health and Wellbeing (MENT) research group, Faculty of Medicine and Pharmacy. Vrije Universiteit Brussel. Brussel. Belgium.

* Corresponding author: Dagje.boeykens@ugent.be

¶: These authors contributed equally to this work.

&: These authors also contributed equally to this work.

^Membership of the Primary Care Academy is provided in the Acknowledgments.

Abstract

Background

The healthcare system is faced by an ageing population, increase in chronic conditions and multimorbidity. Multimorbid patients are faced with multiple parallel care processes leading to a risk of fragmented care. These problems relate to the disease-oriented paradigm. In this paradigm the treatment goals can be in contrast with what patients value.

The concept of goal-oriented care is proposed as an alternative way of providing care as meeting patients' goals could have potential benefits. Though, there is a need to translate this concept into tangible knowledge so providers can better understand and use the concept in clinical practice. The aim of this study is to address this need by means of a concept analysis.

Method

This concept analysis using the method of Walker and Avant is based on a literature search in PubMed, Embase, Cochrane Library, PsychInfo, CINAHL, OTSeeker and Web of Science. The method provides eight iterative steps: select a concept, determine purpose, determine defining attributes, identify model case, identify additional case, identify antecedents and consequences and define empirical referents.

Results

The analysis of 37 articles revealed that goal-oriented care is a dynamic and iterative process of three stages: goal-elicitation, goal-setting, and goal-evaluation. The process is underpinned by the patient's context and values. Provider and patient preparedness are required to provide goal-oriented care. Goal-oriented care has the potential to improve patients' experiences and providers' well-being, to reduce costs, and improve the overall population health. The challenge is to identify empirical referents to evaluate the process of goal-oriented care.

Conclusion

A common understanding of goal-oriented care is presented. Further research should focus on how and what goals are set by the patient, how this knowledge could be translated into a tangible workflow and should support the development of a strategy to evaluate the goal-oriented process of care.

Keywords

goal-oriented care, goal-setting, patient-centeredness, chronic conditions, multimorbidity, review, concept analysis

Introduction

The healthcare system is faced by an ageing population and an increase in chronic conditions and multimorbidity [1]. More and more people are forced to live with the consequences of these demographic changes and require ongoing (chronic) care on top of acute care [2]. At the same time, patient autonomy is gaining importance and patients are considered as an active and important partner in their care [3, 4]. Patients with chronic conditions are often consulting multiple health care providers [3] leading to a higher rate of encounters. They also receive a larger amount of prescriptions [5] and they are asked to complete a diverse set of self-monitoring tasks such as managing, exacerbations or monitoring biomedical targets [3]. Since patients with (multiple) chronic conditions are faced with multiple parallel care process for their different conditions, there is a considerable risk of fragmented care. Especially when healthcare providers focus on disease control, patients can experience lack of care continuity and issues with communication as patients themselves focus on the meaning of care and more on personal wellbeing [6, 7]. As a result, treatment goals can be in contrast with what patients value in their personal lives [3].

The healthcare system is oriented towards a disease-oriented paradigm to which many of these problems relate [8-10]. In this paradigm, care is mainly organized according to disease-oriented guidelines [10]. This may work well for patients with a single disease, but becomes inappropriate for patients with multiple problems. The focus on single disease guidelines might distract providers from what really matters to the patient [10]. A possible way to overcome many of the challenges is to shift care back from 'what's the matter with the patient' to 'what matters to the patient'. It creates healthcare processes in which patients' needs are actively sought and met [9]. Meeting those patients' needs and tailoring care more to what patients want in a co-creation process could result in better social well-being, physical well-being, and satisfaction for patients and healthcare providers [11].

One of the possible strategies is to actively engage patients in identifying their personal goals and aligning care to those goals, which could be achieved by goal-oriented care [12]. The concept of goal-

oriented care has been launched and mentioned for the first time in 1991 by Mold who proposed the concept as an alternative way of providing care [13]. Later on, in 2012, Reuben and Tinetti took the concept of goal-oriented care a step forward by stating that care "must above all consider patients' preferred outcomes" [10]. The focus on setting goals based on the patients' needs and preferences rather than on health-related outcomes became one of the main novelties in chronic disease management [4]. Not only could goal-oriented care be proposed as an important paradigm to overcome some of the new challenges for chronic patients [9], it might also corresponded to the original concept of evidence based medicine (EBM) [14]. EBM was first published by Sackett in 1996 who described three key components: 1. best external evidence, 2. individual clinical expertise, and 3. patients' values and expectations [14]. Since the first description of EBM, multiple approaches and paradigms has been developed to compromise between those three components [15]. For example, patient-centered care (PCC), which is already a well-known and widely used concept, is defined as "providing care that is respectful of, and responsive to individual patient preferences, needs, and values and ensuring that patients values guide all clinical decisions" [15]. Shared-decision making, on the other hand, also strives to share evidence and engage patients in care as it is "an approach where clinicians and patients share the best available evidence when faced with the task of making decisions, and where patients are supported to consider options, and to achieve informed preferences" [16]. Goal-oriented care is proposed as a promising healthcare paradigm and approach to operationalize EBM and return to where it all started [10]. However, in contrast to the other approaches and paradigms, goal-oriented care is ill defined. Developing a common understanding on the concept could potentially contribute to the clarification and in-depth comparison between the related concepts and eventually lead to better use in clinical practice. However, some healthcare providers might already assume that they practice goal-oriented care spontaneously, but there still is a lack of underpinning knowledge and guidance on how to provide goal-oriented care to patients. The main pitfall in most of these goal-setting activities is that the goals are not necessarily related to the patients' needs and preferences while in goal-oriented care these patients' needs and preferences are put on the forefront

and are not necessarily health-related. [17, 18]. From this perspective, goal-setting and goal-oriented care should be taken together and focus on the patients' needs and preferences.

As a first step in exploring the potential of goal-oriented care in chronic care, it is important to gain in-depth knowledge on what goal-oriented care is about and how it can be generally described.

As goal-oriented care could be well-suited in primary care, as this context is often the linchpin for patients with chronic conditions, this will be the focus of this study [19]. This study aimed to describe a structured approach to deepen the concept of goal-oriented care for patients with chronic conditions or multimorbidity in the primary care context.

Method

This concept analysis aims to present an overview and synthetization of the existing literature regarding goal-oriented care for chronically ill patients in primary care. This will be performed by analyzing the concept into antecedents, attributes, and consequences following the method of Walker and Avant [25]. This method provides a framework of eight iterative steps: 1. select a concept, 2. determine the aims or purposes of analysis, 3. identify all concept definitions and select the literature, 4. determine different attributes, 5. identify a model case, 6. identify an additional case, 7. identify antecedents and consequences, and 8. define empirical referents [25]. In this concept analysis the attributes are the heart and will present the characteristics of goal-oriented care and allow the broadest insight into the concept [25].

Step 1: select a concept

Goal-oriented care has been defined as an underpinning strategy for primary care reform in Flanders, Belgium. The concept is presented as one of the main topics of 'The Primary Care Academy' (PCA). The PCA is a consortium consisting of four universities (Ghent University, University of Antwerp, Catholic university of Leuven, Vrije Universiteit of Brussels), six universities of applied sciences (UAC VIVES, UAC

Artevelde, UAC Ghent, UAC Leuven-Limburg, UAC Karel de Grote, UAC Thomas More), and important stakeholders (Flemish Patient Platform and White-Yellow Cross; a home care organization) in Belgium with the aim to strengthen the primary care organization and delivery. The PCA includes experts in primary care from a variety of healthcare and welfare disciplines. Discussions in the research group working on goal-oriented care created a necessity to clarify the concept.

Step 2: determine the aims and purposes of the analysis

The aim of this concept analysis is to build a common understanding to eliminate ambiguity between the concepts related to goal-oriented care. Specifically, the scope of the concept analysis is to define goal-oriented care for people with chronic conditions at the level of primary care.

Step 3: select the literature

The literature search was conducted between January 2020 and April 2020. As the method of a concept analysis does not specify how the literature search has to be performed, this search was based on the method of a scoping review described by Levac (2010) [26]. A preliminary combination of search terms was identified: 'goal-oriented care', 'chronic care', and 'primary care'. Based on these keywords a first search was performed to identify adjacent terms in the literature. The search strategy was revised in consultation with the librarian of the university and the senior researchers. The definitive keywords were: 'goal-oriented care', 'goal-oriented medical care', 'person-centered goal-setting', 'patient-centered goal-setting', 'goal-oriented patient care', and 'patient priorities', emphasized goal-oriented care and it synonyms. Related concepts such as patient-centered care, value-based care, etc. were not included as the method of concept analysis prescribes to deepen all the attributes of one concept. In a first phase, the keywords were entered in PubMed, Embase, and Cochrane Library (Table 1). In a second phase, CINAHL, OTSeeker, PsycINFO, and Web of Science were consulted and confirmed the first results as no new studies were identified

Table 1. Overview of the search strings.

**PubMed**
(goal-directed care[MeSH Terms]) OR goal-oriented care [Title/abstract]) OR goal-oriented medical care [Title/abstract]) OR person-centered goal-setting [Title/abstract]) OR patient centered goal-setting [Title/abstract]) OR goal-oriented patient care[Title/abstract]) OR patient priorities [Title/abstract])
**Embase**
**'goal-oriented care'**:ab,ti OR '**goal-oriented medical care'**:ab,ti OR '**person-centered goal-setting'**:ab,ti OR '**patient centered goal-setting'**:ab,ti OR **'goal-oriented patient care'**: ab,ti OR '**patient priorities'**:ab,ti
**Cochrane**
goal-oriented care in Title Abstract Keyword OR goal-oriented medical care in Title Abstract Keyword OR person-centered goal-setting in Title Abstract Keyword OR patient-centered goal-setting in Title Abstract Keyword OR goal-oriented patient care OR patient priorities in Title Abstract Keyword - (Word variations have been searched)

Articles resulting from this search were put in Rayyan [27] to administer the data. A first selection based on title and abstract was performed with regard to the predefined in- and exclusion criteria. Inclusion criteria: (a) goal-oriented care as a health-related concept, (b) mentioning goal-setting, goal-oriented care or related concept (e.g. person-centered integrated care), and (c) focusing on patients with one or more chronic conditions . Exclusion criteria: (a) focusing on single-disease management, (b) goals regarding disease-specific outcomes (e.g. cancer or diabetes), (c) focusing on goal-oriented care in a specific context (e.g. rehabilitation center), and (d) specifically mentioning patient-centered care, shared-decision making, etc. as they will hamper the understanding of specifically goal-oriented care. Articles resulting from this first search were subjected to a full text screening based on the initial criteria and: (a) full text available, (b) written in English, (c) referring to goal-oriented care or related

concepts as a concept, and (d) containing information of a theoretical building of a definition. There was no restriction by study design to gain as much insight in goal-oriented care from different data sources.

Step 4: defining the attributes

The determination of the attributes started with a discussion of four key articles [1, 6, 28, 29] selected by the first author based on the divers approaches of goal-oriented care and presented to the research group. Similar to a qualitative, thematic analysis, the key articles were analyzed based on an open coding and then grouped into codes (Table 2 – example of data analysis). These codes were then presented to and discussed with the co-authors. In these discussion rounds, codes were translated into attributes. In a second phase, new articles were added and analyzed based on the same method as the key articles until all relevant literature (based on the inclusion criteria) was included. The different codes were put into NVIVO12 to synthesize the data and to initiate further discussion with the research group. This resulted in the final attributes (Table 4). The method starting from reading the first article to defining the attributes was characterized by an iterative process in which the attributes were reformulated until consensus with the research group was reached.

Table 2. Example of analysis process of the study of Bernsten et al. 2018.

Extract from article	Code	Attribute
…A professional and a personal goal clashes in a decision process regarding the discontinuation of a medication the informant had been using for years…	Negotiation goals between professionals and patients.	Goal-setting – patient-provider interaction
… However “What matters to you?” gave a richer and more immediate insight into areas threatened by health issues…	Patient centeredness	Tailoring to patients’ needs and preferences
…Goal evaluation serves as feedback to all contributors in the seamless care process… The result should be documented and linked back to goal adjustment and learning for the next cycle…	Feedback to the care process	Goal-evaluation

Step 5: identify a model case, a contrary case, and a borderline case

A model case is presented as a narrative of how goal-oriented care could be conceptualized and illustrates all defined attributes of goal-oriented care [25]. A contrary and borderline case differ from this model case and do not include all of the attributes and/or differ in one of them.

Step 6: identify antecedents and consequences

Antecedents are events or incidents that precede the process of applying goal-oriented care. Consequences are those events or incidents as a result of applying goal-oriented care [25].
The antecedents and consequences were searched simultaneously with the attributes (step 4). Results have been discussed by the entire research group until consensus was reached.

Step 7: define empirical referents

Empirical referents provide an overview of the identified assessment tools related to the attributes aiming to make the concept, goal-oriented care, measurable. These assessment tools may be seen as the underpinning needs and characteristics when developing an evaluation method of goal-oriented care.

Results

Step 1-3

A first search based on the predefined terms (Table 1) resulted in 590 articles; 82 from Cochrane Library, 188 from Embase, and 313 from PubMed. After removing the duplicates, 366 articles were screened by title and abstract yielding 68 articles. A full text screening of these 68 articles lead to 15 articles that fitted the predefined in- and exclusion criteria (step 3). Based on the snowballing method of adding new articles based on references, citations, and similar articles 22 additional articles were added. This resulted in a total of 37 articles (Fig. 1 and Table 3) that were selected for the full text analysis. These articles represented a broad range of study types: 4 systematic reviews, 4 experimental studies (e.g. randomized controlled trial), 13 qualitative studies, 3 survey studies, 1 concept analysis, 1 methodology paper, 4 reviews, 2 position papers, 1 background paper, 1 status report, 1 commentary, 1 opinion paper, and 1 perspective.

Fig. 1 Flow chart demonstrating the search string.

Table 3. Overview of the included articles.

**Papers identified based on full text screening**				
No.	Year	Authors	Title	Study design + method
1	1991	Mold, Blake, Lorne, Becker [13]	Goal-oriented medical care.	Position paper
2	2011	De Maeseneer, Boeckxstaens [30]	Care for non-communicable diseases (NCD's): time for a paradigm-shift.	Opinion paper
3	2012	Reuben, Tinetti [10]	Goal-oriented patient care- an alternative health outcomes paradigm.	Perspective
4	2014	Bayliss, Bonds, Boyd, Davis, Finke, Fox, Stange [31]	Understanding the context of health for persons with multiple chronic conditions: moving from what is the matter to what matters.	Forty-five experts met to critically consider four aspects of incorporating context into research on multiple chronic conditions.
5	2014	Kramer, Bauer, Dicker, Durusu-Tanriover, Ferreira, Rigby, van Hulsteijn [8]	The changing face of internal medicine: patient- centered care.	Position paper
6	2015	Bernsten, Gammon, Steinsbekk, Salamonsen, Foss, Ruland, Fonnebo [32]	How do we deal with multiple goals for care within an individual patient trajectory? A document content analysis of health service research papers on goals for care.	Document content analysis of seventy health service research papers on the topic of 'goals of care'.
7	2016	Blom, Elzen, Houwelingen, Heijmans, Stijnen, Van Den Hout, Gussekloo [33]	Effectiveness and cost-effectiveness of a proactive, goal-oriented, integrated care model in general practice for older people. A cluster randomized controlled trial: integrated systematic care for older people-the ISCOPE study.	Cluster randomized controlled trial –intervention group: general practitioners made an integrated care plan using functional geriatric approach; control group: care as usual; 59 general practices were included (30 intervention, 29 control); outcome measures on quality of life, activities of daily living, satisfaction with delivered healthcare, and cost-effectiveness of the intervention 1-year follow-up.

8	2016	Boeckxstaens, Willems, Lanssens, Decuypere, Brusselle, Kühlein, Sutter [34]	A qualitative interpretation of challenges associated with helping patients with multiple chronic diseases identify their goals.	Qualitative research – qualitative interviews with nineteen patients diagnosed with chronic, obstructive pulmonary disease and comorbidities to explore goal-setting in patients with multimorbidity.
9	2016	Mangin, Stephen, Bismah, Risdon [35]	Making patient values visible in healthcare: a systematic review of tools to assess patient treatment priorities and preferences in the context of multimorbidity.	Systematic review – data sources: Medline, Embase, Cochrane databases; citations were included if they reported a tool to use a record patient priorities or preferences for treatment, and quantitative or qualitative results following administration of the tool.
10	2016	Schimdt, Babac, Pauer, Damm, von der Schulenberg [36]	Measuring patients priorities using the Analytic hierarchy process in comparison with best-worst scaling and rating cards: methodological aspects and ranking tasks.	Analysis of the results of non-standardized Analytic Hierarchy Process (AHP)for different consistency ration threshold, aggregation methods, and sensitivity analysis; comparison of rakings criteria of AHP with best-worst-scaling and ranking cards results by Kendall’s tau b.
11	2016	Tinetti, Esterson, Ferris, Posner, Blaum [1]	Patient priority-directed decision making and care for older adults with multiple chronic conditions.	Review
12	2018	Bernsten, Hoyem, Lettrem, Rul, Rumpsfeld, Gammon [6]	A person-centered integrated care quality framework, based on qualitative study of patient's evaluation of care in light of chronic care ideals.	Qualitative evaluative review of the individual patient pathways experiences of nineteen strategically chosen persons with multimorbidity.
13	2019	Feder, Kiwak, Costello, Dindo, Hern, Bigos, Naik [3]	Perspective of patients in identifying their values-based health priorities.	Qualitative study using in-depth semi structured telephone and in-person interviews; open-ended questions about patient perceptions of the patient health priorities identification process, perceived benefits of the process, enables and barriers to PHPI, and recommendation for process enhancement.
14	2019	Franklin, Lewis, Willis, Roger,	Controlled, constrained or flexible? How self-management goals are shaped by patient-provider interactions.	Conversation analysis; observations of consultations for chronic care management between patients and their health professionals.

		Venville, Smith [37]		
15	2019	Tinetti, Dindo, Smith, Blaum, Costello, Ouellet, Naik [38]	Challenges and strategies in patient's health priorities-aligned decision-making for older adults with multiple chronic conditions.	Participant observation qualitative study – clinicians followed a training and had experiences in providing patient priorities care (PPC), clinicians and PPC implementation team participated in 21 case-based, group discussions. Using emergent learning, participants discussed challenges, posed solutions, and worked together to determine how to align care options with the health priorities of 35 patients participating in the patient priorities care pilot.
Papers identified through snowballing				
No.	Year	Authors	Title	Study design
16	2006	Hurn, Kneebone, Cropley [39]	Goal setting as an outcome measure: a systematic review	Systematic review – data sources included a computer-aid literature search of studies examining the reliability, validity, and sensitivity of goal-setting/ goal-attainment scaling, with snowballing.
17	2009	Bodenheimer, Handley [40]	Goal-setting for behavior change in primary care: an exploration and status report.	Exploration and Status report – literature search on goal-setting interventions for promoting behavior change; resulting in eight articles.
18	2011	Junius-Walker, Stolberg, Steinke, Theile, Hummers-Pradier, Dierks [41]	Health and treatment priorities of older patients and their general practitioners: a cross-sectional study.	Cross-sectional study – 123 older patients and 11 general practitioners evaluated the importance and severity of patients' individual health problems. Patients received a geriatric assessment, then GPS rated the importance and components of severity of each problem; assessing proportion of important problems and the chance corrected agreement; multilevel logistic regression models were used to relate the importance of a problem with its severity components.

19	2012	Rijken, Bekkema, Boeckxstaens, Schellevis, De Maeseneer, Groenewegen [2]	Chronic disease management programs: an adequate response to patients' needs?	Survey among country-experts resulting in information about existing disease management programs; in addition scientific literature.
20	2014	Lenzen, Daniëls, van Bokhoven, der Weijden, Beurskens [42]	Setting goals in chronic care: shared decision making as self-management support by the family physician.	Background paper to contribute to the understanding of goal-setting within self-management and to identify elements that need further development for practical use.
21	2016	Steel Gray, Wodchis, Upshur, Cott, McKinstry, Mercer, Palen, Ramsay, Thavorn [43]	Supporting goal-oriented primary health care for seniors with complex care needs using mobile technology: evaluation and implementation of the health system performance research network, Bridgepoint electronic patient reported outcome tool.	Pragmatic cluster randomized controlled trial – intervention groups using ePRO tool compared with control groups on measure of quality of life, patient experience, and cost-effectiveness; evaluating of tool.
22	2017	Kangovi, Mitra, Smith, Kulkarni, Turr, Huo, Glanz, Grande, Long [44]	Decision-making and goal-setting in chronic disease management: baseline findings of a randomized controlled trial.	Randomized controlled trial – patients used low-literacy aid to prioritize one of their chronic conditions and then set a goal for that condition with their primary care provider; patients created patient-driven action plans for reaching these goals.
23	2017	Mold [45]	Goal-directed health care: redefining health and health care in the era of value-based care.	Review
24	2017	Schellinger, Anderson, Frazer, Cain [46]	Patient self-defined goals: essentials of person-centered care for serious illness.	Descriptive qualitative analysis – initial inquiry to describe self-defined goals patients living with advanced heart failure, cancer, and dementia; goals were entered in electronic health record flow sheet using patients' quotes; analysis of 160 flow sheets with a deductive approach.
25	2017	Vermunt, Harmsen, Elwyn, Westert, Burgers, Rikkert, Faber [47]	A three-goal model for patients with multimorbidity: a qualitative approach.	Qualitative study – qualitative interviews with general practitioners and clinical geriatricians and analyzed following a thematic approach.

26	2017	Vermunt, Harmsen, Westert, Rikkert, Faber [17]	Collaborative goal setting with elderly patients with chronic disease or multimorbidity: a systematic review.	Systematic review based on EPOC, PRISMA and MOOSE guidelines; Pubmed, PsychInfo, CINAHL, Web of Science, Embase, Cochrane Central Register of Controlled Trials were searched systematically; eligibility criteria: 1) Randomized (cluster) controlled trials, non-randomized controlled trials, controlled before-after studies, interrupted time series or repeated measures study design; 2) Single intervention directed specifically at collaborative goal setting or health priority setting or a multifactorial intervention including these elements; 3) Study population of patients with multimorbidity or at least one chronic disease (mean age ± standard deviation (SD) incl. age 65). 4) Studies reporting on outcome measures reducible to outcomes for collaborative goal setting or health priority setting.
27	2018	Kessler, Walker, Sauvé-Schenk, Egan [29]	Goal setting dynamics that facilitate or impede a client-centered approach.	Conversation analysis on goal-setting conversations; purposively selected from a pilot randomized controlled trial of OPC-stroke
28	2018	Naik, Dindo, Van Liew, Hundt, Vo, Hernandez-Bigos, Esterson, Geda, Rosen, Blaum, Tinetti [4]	Development of a clinically feasible process for identifying individual health priorities.	Prospective development and feasibility study – development team of patients, caregivers, clinicians using a user-centered design to develop and refine value-based patient priorities care process and medical record template; descriptive statistics and qualitative analysis of barriers and enablers.
29	2019	De Groot, Schönrock-Adema, Zwart, Damoiseaux, Jaarsma, Mol, Bombeke [48]	Learning from patients about patient-centeredness: a realist review: BEME guide No.60	Realist review – realist review approach; literature search in scoping phase, deductive and inductive coding to extent rough program theory.

30	2019	Kuluski, Guilcher [49]	Towards a person-centred learning health system: understanding value from the perspectives of patients and caregivers.	Commentary; call to action to combine the tenets from person-centered care, value-based healthcare, and learning health systems.
31	2019	Kuluski, Peckham, Gill, Gagnon, Wong-Cornall, McKillop, Parsons, Sheridan [9]	What is important to older people with multimorbidity and their caregivers? Identifying attributes of person centered care from the user perspective.	Qualitative descriptive study; 1-1 interviews semi-structured interviews with 172 patients and caregivers from 9 community based primary healthcare.
32	2019	Reuben, Jennings [12]	Putting goal-oriented patient care into practice.	Review
33	2019	Salter, Shiner, Lenaghan, Murdoch, Ford, Winterburn, Steel [28]	Setting goals with patients living with multimorbidity: qualitative analysis of general practice consultations.	Qualitative analysis of general practice consultations – analysis of video recorded doctor-patient interactions; focus groups to identify core challenges of goal-setting.
34	2019	Tinetti, Naik, Dindo, Costello, Esterson, Geda, Rosen, Hernandez-Bigos, Smith, Ouellet, Kang, Lee, Blaum [50]	Association of patient priorities-aligned decision-making with patient outcomes and ambulatory health care burden among older adults with multiple chronic conditions.	Nonrandomized clinical trial with propensity adjustment conducted at one patient priorities care (PPC)and one usual care; participants included 163 adults aged 65 years or older who had three or more chronic conditions care for by ten primary care practitioners (PCP) trained in PPC and 203 similar patients who received usual care from 7 PCPs not trained in PPC.
35	2020	Eckhoff, Weiss [51]	Goal-setting: a concept analysis	Concept analysis – method of Walker and Avant, articles and book chapters were reviewed from Cumulative Index to Nursing and Allied Health Literature, Education Resources Information Center, Psych Index.
36	2020	Purkaple, Nagyaldi, Todd, Mold [52]	Physician's response to patient's quality-of-life goals.	Randomized controlled trial – patients were given a previsit questionnaire that included quality of life questions; physicians in the control were given no further prompting; intervention physicians were prompted to ask quality of life questions; a two-

				pronged design was used: prepost group where three physicians participated in 5 control and 5 intervention encounters (n = 30) and a randomized group in which 11 physicians and their patients were randomly assigned to control or intervention groups (n = 30). Video recordings of the encounters were reviewed to determine if QOL goals were mentioned and if they were utilized in decision making.
37	2020	Sathanpally, Sidhu, Fahami, Gillies, Kadam, Davies, Khunti, Seidu [53]	Priorities of patients with multimorbidity and of clinicians regarding treatment and health outcomes: a systematic mixed studies review.	Systematic review – MEDLINE, EMBASE, CINAHL, and Cochrane databases were searched; included studies reported health outcome and treatment priorities of adults with multimorbidity, defined as suffering from two or more chronic conditions, or of clinicians in the context of multimorbidity or both; no restriction by study design, and studies using quantitative and/ or qualitative methodologies were included.

Step 4: attributes

The systematic analysis of the 37 selected papers could identify many different attributes of goal-oriented care (S1 Table 1). Synthesizing these attributes, goal-oriented care could be described as a multifaceted dynamic and iterative process of care (first main attribute) underpinned by patients' values (second main attribute). For the process of goal-oriented care five sub attributes and seven descriptive items could be identified (Table 4). These attributes interact and cannot be interpreted separately.

Table 4. Overview of attributes.

<table>
<tr><td rowspan="8">Goal-oriented care is a multifaceted, dynamic and iterative process.
[1, 3, 4, 6, 12, 13, 17, 28, 29, 40, 42, 43, 46, 54, 55]</td><td colspan="2">1.1 Goal-elicitation builds a patient-provider relationship. [1, 28, 29, 45, 56]</td></tr>
<tr><td rowspan="4">1.2 Goal-oriented care entails goal-setting.</td><td>1.2.1Patient-provider interaction guides goal-setting.[2, 4, 12, 13, 17, 28, 29, 35, 40, 42-45, 47, 49]</td></tr>
<tr><td>1.2.2 Patients' needs and preferences are the foundation of SMART formulated goals. [1-4, 6, 10, 13, 28, 29, 31, 35, 37, 41, 44, 46, 49, 50, 56, 57]</td></tr>
<tr><td>1.2.3 Care plan is based on patients' needs and preferences. [1, 3, 4, 6, 10, 12, 13, 17, 31, 33, 35, 38]</td></tr>
<tr><td>1.2.4 Care is delivered according to the care plan.[1, 6]</td></tr>
<tr><td rowspan="3">1.3 Goal-evaluation is a reflexive process.</td><td>1.3.1 Feedback should be given to the goals. [38, 54]</td></tr>
<tr><td>1.3.2 Evaluation entails questioning how goals are being met. [12]</td></tr>
<tr><td>1.3.3 Goals must be measurable. [13], 33)</td></tr>
<tr><td rowspan="2">2. Goal-oriented care embraces patients' values.</td><td colspan="2">2.1 Goal-oriented care must be placed in patients' context. [3, 12, 31, 35, 42]</td></tr>
<tr><td colspan="2">2.2 Goal-oriented care must be tailored to patients' needs and preferences. [1, 6, 28, 29, 38]</td></tr>
</table>

Goal-oriented care is a multifaceted, dynamic and iterative process

The majority of the authors presented goal-oriented care as a stepwise approach [1, 3, 4, 6, 12, 13, 17, 28, 29, 40, 42, 43, 46, 54, 55]. Even though every paper defined their own approach, overall three stages could be identified: (a) goal-elicitation, (b) the actual stage of goal-setting, and (c) a reflexive goal-evaluation stage. These three stages will be further discussed.

Bernsten et al. emphasized the dynamic and iterative characteristics of the goal-oriented process of care [6]. They described that goal-oriented care entails going back and forth between the three stages [6]. From this perspective, goals are not described as an endpoint, but they can be adjusted, discarded, modified or new goals might be set [12, 38]. This will be further discussed in the stage of goal-evaluation.

Overall, in the goal-oriented process of care, the patient is described as an active partner [1]. Therefore, a good communication in a continuous patient-provider relationship is described to be of utmost importance [46]. In addition, goal-oriented care should be considered as care over time rather than a one-time intervention [58]. In terms of outcomes, it is not entirely clear whether goal-oriented care should focus on (a) maintaining the status quo or (b) improving the patients' situation [12]. Although there is consensus that the care process is oriented to the current needed care rather than care needed in the future [1].

Goal-elicitation builds a patient-provider relationship

As described earlier, the overall analysis could identify goal-elicitation as the first stage in the process of goal-oriented care. In this first stage, providers are presumed to offer time and space to patients to tell their stories in order to work towards the patients' agenda [29]. Therefore, patients have to be ready and should be actively encouraged to tell their story. Tinetti and colleagues described this as 'the patient's state of readiness' [1]. This first stage is considered to be essential to work towards a balanced patient-provider conversation and relation. Salter et al. described this stage as a shared process between patients and providers that reinforces and further builds their relationship [28]. This

specific part of the process of goal-oriented care is also described as a mean to achieve a greater level of shared understanding and mutual commitment between the patient and the provider [45]. Specific attention to the stage of goal-elicitation is described to create a supportive context for effective goal-setting in the next stage [28].

Goal-oriented care entails goal-setting

Besides the goal-elicitation stage, the literature identifies a goal-setting stage. Franklin and colleagues analyzed patient-provider conversations during goal-setting and concluded that the goal-setting stage serves as a mechanism to embrace patients' needs within the social context he lives in [37]. When this process is done properly, goal-setting should support the patients to continue doing what matters most to them which would help them to cope with their conditions [37]. Within this process of goal-setting different sub attributes, that are considered necessary for proper goal-setting, could be identified.

Patient-provider interaction guides goal-setting

The patient-provider interaction is characterized by a patient-centered approach [28] in which goals are set in collaboration [47]. Hereby, patients and providers agree on health-related goals [2, 12, 13, 40, 43, 47, 55, 59] and find common ground [58]. Tinetti et al. described the importance of considering patients as active partners in the goal-setting process [38]. Rijken et al. mentioned that patients' goals have to be discussed in a dynamic conversation continuously taking the patients' needs, preferences, and abilities into account [2].

To facilitate a collaborative approach it is suggested that providers emphasize the patients' narratives reflecting their lived experience [45]. Besides a collaborative approach, negotiation is important and considered inevitable [4, 6, 28, 42, 54]. Lenzen et al. defined this as goal-negotiation, which involves discussion of any kind of problems, exploration of the patients' values, needs and capabilities, and deliberation on patients' goals [42]. In goal-negotiation, formulating and agreeing on a specific goal are important components [28].

Because the goal-setting process needs to be driven by patients' needs and preferences, there seems to be a general understanding to shift the focus from the provider to the patient [29]. Different authors reported various strategies to facilitate this shift. Mold stated that the shift implies that prioritization of the individual health-related goals and the amount of effort in achieving them should be made by the individual [13]. Naik et al. stated that patients are indeed encouraged to share their priorities, but adds that providers are encouraged to align their care with the patients' health priorities [4]. More recent publications talking about goal-setting describe a circular and shared process aimed at improving the balance and power differentials in the patient-provider relationship [4, 44]. This balance can be improved by putting themselves in someone's shoes to understand the other's constraints [49].

Patients' needs and preferences are the foundation to set goals

One of the important challenges in our understanding of the concept of goal-oriented care is the lack of clear understanding on patient goals. Nearly all authors described that goals should be grounded on the patients' needs and preferences [1-4, 6, 28, 29, 37, 38, 44, 46, 52, 54, 58, 60, 61]. It is described that goals should be based on the context, resources and capabilities of patients [52], that they should be approved by patients [6], and that they should foremost represent what the patients want and not necessarily what the providers want [12, 46]. Other authors recommended that goals should be a combination of both the patients' goals and the providers' goals which in turn is related to goal-negotiation [29, 49]. In conclusion, no overall understanding on the goals could be formulated.

Besides this lack in understanding, there also seems to be ambiguity about the categorization of goals. Some authors emphasized that goals should contain core values of patients (e.g. the broader aspects that matter most to the patient) [1, 4] . These goals are named as 'overarching goals' [6, 12, 29, 46] leading to a broad description of the goal (e.g. I want to live in my own home as long as possible [1]) [6]. Others argued that these overarching goals might not be easy to work with and describe that these goals should be broken down into sub goals (e.g. I want to walk 2 blocks without shortness of breath [1]) [6]. Goals differ for each individual and will change over time [13]. Aside from overarching goals

and sub goals many of the authors mention the importance of setting SMART goals [1, 6, 28, 29, 40, 51, 54, 55, 58]. A SMART goal is created when patients and providers collaborate to untangle the goal itself, the importance of that goal is emphasized to the patient, the perceived achievability of the goal is evaluated, as well as the timing of the goal, and any supports and resources available [40]. On the meta-perspective, overarching goals are too broad to make SMART (think about the grandmother aiming to get her grandchildren from school as long as possible). Therefore they should be divided in the sub-goals (such as I need to be able to walk without being tired after 10 yards) that are specific enough to be measured.

In one of his first publications Mold brings in a specific discourse around the categorization of goals, namely that goal-oriented care should assist patients in achieving their maximum individual health potential [13], hereby making the link with health. One should however notice that health should be described from the patients' perspective; as the ability to live his life, and not as the absence of disease [1, 13]. Patients' goals are oriented towards health outcome goals. Patients hope to achieve these individual health outcomes through their health care (e.g. function, social activities, and symptom relief)[1]. Health outcome goals describe activities that promote change in physical and cognitive well-being or health [41]. Naik et al. specifically relate patient goals to the care they are willing to receive and able to perform [4].

Care plan is based on patients' needs and preferences

Many authors relate goal-oriented care to the construction of a care plan based on the patients' needs and preferences and specifically mention that these care plans should reflect the patients' personal goals that have been identified in the previous stage [1-3, 6, 12, 31, 33]. There is a consensus that the care plan should reflect the question: 'What matters to you?' [12, 38, 49, 54, 60]. Strategies to achieve the patients' needs and preferences should be implemented in the care plan [13]. Furthermore, Bernsten and colleagues stated that the care plan might also include an interprofessional review of the goals [6]. Therefore, it is necessary to involve all providers and preferably patients' informal caregivers

and family in the whole process [3, 6, 17]. In case that providers are confronted with patients' goals that are out of their own scope, they could benefit from an interprofessional review as they are enabled to discuss with and hand over to other providers with the required expertise. This could improve the coordination of the care plans between the different providers and facilitate integrated care delivery [1, 4, 35]. To guide this interprofessional review, no specification was given about which profile would be the best fit for having the lead. Vermunt et al. (2017) illustrated this as they found variation in who (e.g. GP, nurse, practice nurse, psychological wellbeing practitioner) should contribute to goal-setting [17].

Care delivery according to the care plan

Patients and providers should implement the care plan and translate it into care delivery. Although, little is known about how care should be delivered, it is evident that it must be in accordance with the care plan that is set up in the previous stage [6]. For this stage Tinetti et al. specifically mentioned to start the stage of care delivery by prioritizing on simple interventions in order to achieve one or more small goals to keep patients motivated [1]. This simple interventions could focus on the sub-goals described in previous paragraphs to eventually work towards the overarching goals.

Goal-evaluation is a reflective process

The overall synthesis/analysis of the literature could identify goal-evaluation as the third and final stage in the process of goal-oriented care. For this stage authors described a dynamic and iterative process that allows reflection and feedback next to assessing whether and how goals have been met [38, 54]. In this process goals can be redefined and adjusted. Possible reasons to adjust goals might be that goals have been too difficult to achieve or were no longer desired or relevant to the patients' situation [12]. Although many authors acknowledge the possibility and importance of goal adjustment, there is also discussion that goal-oriented processes of care requires that goals can be measured [13]. Steele Gray and colleagues described the importance of qualifying and quantifying the process proceeded to achieve the goals [43]. In contrast, Salter and colleagues described that making the goals

measurable could overcomplicate and distance the patient from their own goal and might therefore not be beneficial to the process of goal-oriented care [28].

Goal-oriented care embraces patients' values

In the previous attributes, goal-oriented care is described as a dynamic and iterative process in which two underpinning values are identified [4]. First, goal-oriented care must be placed in the patient's context and second, goal-oriented care must be tailored to the patient's needs and preferences.

Goal-oriented care must be placed in patients' context

The whole goal-oriented process of care starting from goal-elicitation to goal-evaluation needs to be placed in the patient's context. According to different authors this means that the process must be tailored to the patient's situation [3, 12, 42, 60]. This does not only refer to the personal context, but also to the social and the cultural context. Therefore, this process is influenced by different contextual factors that should must be taken into account when developing the care plan [35, 42].

Goal-oriented care must be tailored to patients' needs and preferences

When reviewing the attributes, it is clear that patients' needs and preferences form the common thread. The question 'What is the matter with the patient' must be retranslated to 'What matters to the patient?' [1, 6, 28, 38]. This question enables patients to tell their story and open up in which they are considered to reflect on their achievements and personal agenda [29]. As a result, patients will have the feeling to be approached as a person instead of through their condition [6].

CASES

The method of Walker and Avant prescribes that several cases should be described to illustrate the attributes defined in step 4 [25]. The first case of Joseph encompasses all the attributes identified in the literature and is therefore identified as a model case. It is a fictive example of delivering care

according to the goal-oriented process of care with focus on the underpinning attributes. The second case of Ben is identified as an additional case as it lacks one or more of the attributes. E.g. in the case of Ben the stage of goal-evaluation is missing. This stage is needed to make adjustment and reflections according to the process of achieving the personal goals. Finally, the third case of Mary is an example of the opposite of goal-oriented care. This is described as a contrary case. In this case, the health care provider does not take the needs and preferences of Mary into account. The provider only thinks about convincing Mary of a healthy lifestyle which for her is not the main reason to visit her health care provider. Her main focus is on being able to go on a city trip to Madrid.

Joseph, 68- year old suffers from diabetes, hypertension and chronic obstructive pulmonary disease. Throughout his entire working life, he was a secondary school teacher. He has been retired for three years now. Despite the fact that he is limited by his health condition, he loves spending time with gardening and playing with his grandchildren.

A few years ago he was a passionate cyclist, but his racing bike has been stored for a long time now. His friends encourage him to cycle with them on a weekly base. His wife supports this initiative and argues that this will be beneficial for his social contact.

Every month Joseph visits his family doctor for a check-up. For each consultation, he prepares a list of things he wants to discuss. He has the chance to share his story in an open communication in which trust and mutual respect are key components.

In his monthly check-up with his family doctor he suggests his wishes to cycle again with his friends. His doctor doubts whether this will be possible and after discussion and negotiation, they plan that he would join his friends in their weekly cycling trip but only for the first two hours. The group will be asked to adapt their pace and Joseph will make sure that he does not need to return back home on his own. The doctor makes adjustments to the medication scheme according to the increased efforts Joseph will make. He will also contact the cardiologist to inform him about the changes to the medication schema. The family doctor and the cardiologist will collaborate in order to succeed in Joseph’s goal.

The family doctor and Joseph agree to discuss and evaluate the course after three months. It is possible to increase or decrease the intensity depending on Joseph's health state and his own preferences.

Box 1 Model case of Joseph

Ben, a 30-year old man, was renovating a house that he bought with his girlfriend when he was diagnosed with MS. They made plans to marry next year and to make a world trip as honeymoon. These plans have been put aside due to the recent diagnosis. Although he was feeling down and did not have the energy to do anything he ended up with an excellent physician. Initiated by the interaction and the conversation with his physician he was enabled to set goals again and to look

Box 2 Additional case of Ben

Mary is a 40-year old mother of two young children and dealing with obesity since her childhood. Due to her weight, she has a lot of joints pain and is short of breath which limits her exercising capacity. Her children are already looking forward to playing outside with their mother during the summer holidays. Unfortunately, she is not able to play soccer or jump on the trampoline because of the pain. The pain becomes too much for her and after long hesitation she discusses this with her physician. The only thing she wants is to play and interact with her children as painless as possible and therefore asks her physician to prescribe some medication. Her physician does not support medication, but instructs her to first strive for a healthy weight as a solution to relieve the pain. This is not aligned with the wishes of Mary who only wanted a short-term solution to be able to play with her children. In the end, she leaves the consultation room with a referral to a dietitian and sport coach.

Box 3 Contrary case of Mary

Antecedents

Antecedents are events or incidents that occur prior to the investigated concept. In this concept analysis, provider preparedness and patient preparedness are required to provide goal-oriented care.

In terms of provider preparedness many authors discussed the importance of training [6, 7, 29, 33, 37, 47, 55]. Notwithstanding that several authors [1, 4, 17, 28, 33, 38, 44] mentioned the importance of trained health care providers, there was a difference in the training they received (supplementary file 3). Differences can be found in the target population reached with the training, both in monodisciplinary and interprofessional training (e.g. general practitioners [28], practice nurses [33], duration of the training (e.g. three hour [28], number of sessions [33]) and training method (e.g. role-play [38]). Thereby, the content of the training was tailored to the skills needed to carry out the intervention correctly and differ therefore in each training (S3 Table 3).

A second aspect that is discussed concerning provider preparedness focused on the personal skills of providers [1, 6, 17, 28]. These include communication and balancing skills in which an open communication with the patient is necessary and in which an equal balance between the patient and provider is a premise [1, 6, 17, 28]. Other defined skills were the provider's ability to listen, understand and bearing witness to the patient's story [28] and their willingness to change and learn new skills to provide care according to the goal-oriented process of care [1].

Besides provider preparedness some authors [1, 12, 47] specifically talked about the need of patient preparedness. Patients needed to be prepared to share their needs and preferences when entering a care relationship [1]. Some authors translate the importance of patient preparedness into patient education [1], others talked about patient guidance (11) or supporting patients in developing the skills to set personal goals [42].

Consequences

Consequences are those events or incidents that occur as a result of a concept. For the concept of goal-oriented care, the consequences defined throughout the papers could be categorized in: (a) patient-related consequences [1, 3, 4, 29, 35, 54], (b) provider-related consequences [1, 28, 35, 54], (c) care-related consequences [1, 28, 35] and (d) general consequences [4, 6, 35].

Patient-related consequences are the results for patients themselves after they received care following a goal-oriented process. A goal-directed approach could be expected to increase patient satisfaction, since the values, preferences, knowledge and opinions that each patient brought to the provider-patient relationship was more valued [45]. Also, emphasis was put on the changed way of communicating in which patients felt more freely and able to speak [3]. This led to the overall feeling of being heard, understood, respected and engaged in their care [35]. Furthermore, a goal-oriented process of care could lead to a better understanding and more in-depth knowledge of patients regarding their health, activation of patients to be more involved in their care and an increase in their overall commitment. This resulted in the increase of adherence [3]. Also Mold argued that it could contribute to a better adherence [13]. In general, the gained in-depth knowledge of patients concerning their health and a better understanding of their tasks could help to improve their quality of life [3]. This was enhanced by the maximization of function and the independence patients gained [13].

For providers, goal-oriented care assisted healthcare them in their decision-making [35] and gave them the opportunity to get to know their patients better. It enhanced patient-provider collaboration [13] and contributed therefore to more job satisfaction [28].

Care-related consequences were mainly focused on reducing costs, overtreatment and fragmentation [1, 28, 35], since care oriented to patients' priorities would reduce tests and treatments [50]. Bernsten

et al. stated also that goal-oriented care could lead to an improvement of quality of care and quality of life [6].

Although, many positive outcomes have been presented, Reuben et al. mentioned a possible downside of goal-oriented care [10]. They described that some decisions to strive for personal goals may worsen the providers' performance on aggregated health measures. For example, when a diabetic patient chooses to not follow his diet and keep on smoking, because it would be a too big lifestyle change, his HbA1c-level would not be aligned with the guidelines. Although, it could be a positive outcome from the patient perspective, it would influence the quality of care provided and the population health in a negative way.

Empirical referents

Empirical referents provide an overview of the identified assessments tools related to the attributes aiming to make the concept measurable.

None of the papers mentioned an empirical referent to measure the entire concept of goal-oriented care. Therefore, tools have been searched for each individual sub-attribute. Examples are listed in Table 5 which gives an overview of possible tools and presents an example item presented in that tool. Listing the existing individual empirical referents might initiate the development of an overall empirical referent.

Table 5. Overview empirical referents.

Attribute	Purpose of the tool	Example of item in the assessment tool
Goal-elicitation		
Davis Observation Code (DOC) [62]	20-item direct observation scale for physician-patient interactions	Discussing family, medical, or social history and/ or current family functioning.
Goal-setting		
Patient goal priority questionnaire [63]	Patient-specific measure for identification of behavioral goals and evaluation of clinically significant changes	Which activities are most important for you to manage?
Self-identified goals assessment [64]	1) Helps patients to identify personally meaningful occupational goals to be addressed in therapy<br>2) evaluate changes levels of patient-defined success in desired occupations	Think about all of the things you want to be able to do. It might help to think about the things you did at<br>home before you went to the hospital, and things that are hard to do now. What types of things would you like<br>to work on or improve on in therapy before you go back home?
Canadian Occupational Performance Measure (COPM) [65]	Measure of a client's self-perception of occupational performance in the areas of self-care, productivity, and leisure	Semi-structured interview – discussing daily functioning and personal life.
Health outcome prioritization tool [66]	Tool for decision-making among older persons with multiple chronic conditions	I would like to know how important 'keeping you alive', 'maintaining independence', 'reducing or eliminating pain' and 'reducing or eliminating symptoms of dizziness, fatigue, shortness of breath' is to you.
Electronic Patient Reported Outcome Tool (ePRO-tool) [67]	Tool can help patients and providers to collaboratively develop healthcare goals	Goal-setting for five different areas identified as most important.
Goal-evaluation		
Goal-attainment scale [68]	Tool to measure in which extent patients' goals have been met	Determining goal-attainment using 5-point scale.
Patient Assessment of Care for Chronic Conditions (PACIC) [69]	Tool to measure quality of chronic disease care	Asked to talk about my goals in caring for my condition.

Goal-setting evaluation tool [70]	Tool to rate the quality of goals and action plans	Does the plan identify specific actions or activities that could help to reach the goal?
Person’s context and patient’s needs and preferences		
Person-centered primary care measure (PCPCM) [71]	11-item patient-reported measure to assess primary care aspects	My doctor or practice knows me as a person/ Over time, the practice helps me to meet my goals.
Patient centered observation form (PCOF) [72]	Tool to help healthcare providers communicate effectively with patients	Collaborative upfront agenda setting.

CONCLUSION OF THE CONCEPT ANALYSIS

Fig. 2 represents the overall synthesis of this concept analysis of goal-oriented care. Goal-oriented care could be described as a health care approach encompassing a multifaceted, dynamic and iterative process underpinned by the patient's context and values. The process is characterized by three stages: goal-elicitation, goal-setting and goal-evaluation in which patients' needs and preferences form the common thread. In order to be able to deliver care according to the principles of the goal-oriented care process, both providers and patients need to be prepared. In terms of the consequences of goal-oriented care literature points to the potential of goal-oriented care to improve patients' experiences and provider well-being, the potential to reduce costs and improve the overall health of the population. Furthermore, a model, a contrary and an additional case illustrated an example of goal-oriented care in practice. The empirical referents showed that it is currently not possible to measure goal-oriented care in its entirety and presented an overview of possible referents for each sub attribute. Although the literature allowed us to gain more insight into the concept of goal-oriented care, different aspects need to be further discussed.

Fig. 2 Schematic representation of the antecedents, attributes and consequences.

Discussion and conclusion

This concept analysis aimed to tackle the lack of a common understanding of goal-oriented care by identifying the attributes, antecedents and consequences using the method of Walker and Avant [25]. The overall analysis showed that a goal-oriented care generally entails three stages. Despite these three stages the process of goal-oriented care cannot be implemented as a linear protocol or checklist. Two underpinning attributes, the patient's context and the patient's needs and preferences form the common thread throughout this goal-oriented process of care. These underpinning attributes represent the philosophy of care. Goal-oriented care is a continuous interaction where you go back and forth to gain a person-centered approach (Fig. 2).

In the stage of goal-elicitation, greater consideration should be given to the patients' peripheral narrative reflecting their lived experiences [37]. Several authors have investigated components of goal-elicitation. Murdoch and colleagues performed a conversation analysis of patients-providers interaction during their encounters and found that eliciting the patients' understanding is an important component [73]. Ospina et al. investigated the extent to which patients' concerns are elicited across different clinical settings [74]. They concluded that providers seldom elicit the patients' agenda. This reduces the chance that providers will orient their consultation towards the specific aspects that matter to the patient [74]. One of the prerequisites to succeed in goal-elicitation is the mutual understanding about the expectations of the consultations between patients and providers and a qualitative relationship between patients and providers [73]. The literature also mentions that patients need to have a set of skills to make appropriate health decisions and reflect on their health care choices [75]. They have to be capable to open up and tell their story [76]. It is important that patients understand the meaning of information communicated by the provider, must appreciate the consequences of the treatment options, and must reason about the information based on his or her own values and preferences [76].

Besides the stage of goal-elicitation, the stage of goal-setting was defined. One of the remaining knowledge gaps is on what kind of goals patients set. In goal-oriented care it seems important to set goals based on the patients' needs and preferences (e.g. I want to take my grandchildren to school), while in other chronic disease management programs emphasis is mainly still on health-related goals (e.g. I want the patient to walk without pain) [4]. Various work in different settings identified that patients do not necessarily have clearly defined goals for themselves [73]. Although, several authors performed research on the categorization of patients' goals. Vermunt et al. performed for example a qualitative study to develop conceptual descriptions of goal-oriented care [47]. They presented a three-level goal hierarchy containing disease- or symptom specific goals, functional goals, and fundamental goals which provides more insight in the type of goals. A second example is the distinction made by Schellinger et al. between medical, nonmedical, multiple, and global goals [46]. Not only is there ambiguity on what goals patients set, it is also not clear how goals are being set. What is clear is that patients and providers must collaborate and negotiate on which goals are important. Nevertheless, this can still cause conflicts between the patients' goals and providers' goals [31, 66]. To overcome these conflicts, it is suggested to first set the patients' goals and then discuss about the medical goals, because conflicts are more likely when goals are placed on the same level [32]. It should however be noticed that setting the patients' goals on top does not legitimate full patients' responsibility over the care plan [32]. Another way to overcome these conflicts is to work with a facilitator as Naik et al. did in developing their *patients priorities identification process*. These facilitators supported patients in setting goals, choosing the most important goals to eventually communicate them with the provider [4]. Yet another strategy is to use tools to assess patient treatment priorities and preferences. Unfortunately, Mangin et al. found few relevant tools to set patients' goals [35]. They argue for the need to develop specific strategies to make patient priorities visible in the clinical record and medical-decision making [35].

Goal-evaluation was pointed out as the last stage. As presented in the results, several authors described that goals should be made measurable for evaluation [28, 67]. There are some pitfalls

related to goal-evaluation. Salter et al. described that not all goals lend themselves to being measured [28]. It is for example challenging to evaluate the goal ‘I want to take my grandchildren from school as long as possible’. Another pitfall is that patients’ goals would be simplified to what can be measured. Working towards goal-evaluation might increase the pressure on patients and providers to work in the same way as disease-specific guidelines do [77]. Especially from the perspective of patients with multimorbidity it can be questioned whether disease-specific guidelines that are good for the disease are also good for the patient [77]. Furthermore, evidence shows that older multimorbid patients place quantitative health outcomes, such as longer survival, on a lower level of importance [77]. The focus must be on the patients’ values and make healthcare more humane [45].

As mentioned for the antecedents it is important that patients and providers are prepared to work towards a goal-oriented process of care. The collaboration and co-creation between the two partners and in an interprofessional team is an important but insufficient prerequisite to succeed in providing goal-oriented care. Currently patients are not always stimulated to think about their care. They have to be stimulated to actively engage their narrative and to share their priorities. Also providers have to develop complementaty skills in which they learn to let go their own assumptions and solutions. They have to learn to integrate patients’ narrative in their care plan and improve their communication skills to strengthen the mutual understanding between them [78]. Voigt et al. observed that GPs are often unaware of patients’ priorities in daily life, which were in contrast with their perceived importance of patient’s medical goals [78]. Training and tools could provide the guidance needed to improve the communication[1, 4, 17, 28, 33, 38, 44]. It could support providers in structuring the conversation, to set goals in collaboration with patients, and to align their care to those goals. Not only does goal-oriented care offers a specific approach for one-on-one interaction between patients and providers, it could also facilitate interprofessional collaboration. It gives providers from divers disciplines the opportunity to deliver care following the same principles and to focus on pursuing patients’ goals [40]. Therefore training should also include the interprofessional perspective to facilitate a uniform attitude towards the patients’ goals and principles of goal-oriented care in the entire team. This will potentially

support providers to learn from and with each others' expertise and enable discussion between them in case that, for example, patients set goals that our out of the remit of the provider. Besides patient and provider preparedness, it could seem logical that also the system has to be prepared, but the current literature does not point to that.

In terms of the consequences of goal-oriented care, a limited number of studies have been able to demonstrate outcomes of goal-oriented care. Nonetheless, these studies showed mostly positive outcomes towards the patients, providers, health system, and overall population well-being. In that respect, goal-oriented care shows the potential to meet the components of the quadruple aim. It can be questioned if all providers experience increased satisfaction and well-being in providing goal-oriented care. Providers have to learn to cope with another way of delivering care. For example, a changed medication scheme as described in Josephs' case in order to work towards patients' goals. This goes against their basic principles to strive for the best possible health status including a comprehensive medication scheme. Besides that the provider well-being can be questioned, Blom et al. also contradicted the positive results for the health care system. They did not find a beneficial effect in health care use and costs when using a proactive, goal-oriented, integrated care model [33].

One of the reasons of the limited number of effectiveness studies of goal-oriented care is the lack of empirical referents. The concept must still undergo the transition towards an evaluable concept. Boyd et al. argue for measures for quality of care needed by older persons with multimorbidity as the current clinical guidelines have undesirable effects for this population [57]. Goal-oriented care is identified by Etz and colleagues as one of the main constructs when developing a new comprehensive measure of high-value aspects of primary care, however they did not mention how it has to be done [79]. Further Young et al. described outcome goals as a main construct when differentiating processes and outcomes for primary care and divided it further in goal-clarity for multimorbidity, goal-clarity for unique patient priorities and goal timing [80]. It is clear that in order to gain more insight in the consequences of goal-oriented care further research must primarily focus on how goal-oriented care

is provided and can be supported. In order to investigate the potential benefits of goal-oriented care, research also needs to work on developing indicators of the goal-oriented process of care.

Strengths, limitations, and recommendations

The method of Walker and Avant provides a rigorous and systematic approach to refine the concept of goal-oriented care through the existing literature. A concept analysis is an exploration of an evolving concept which will need to be enriched by new knowledge. Therefore, it is influenced by contextual factors and must undergo adjustments to new implications and new insights based on further research. Since there is no specification given by Walker and Avant on how to conduct the literature review, we followed the guidelines from a scoping review as described by Levac (2010) [26]. The iterative process of adding new articles following the snowballing method is one of the strengths compared to other types of reviews. In this concept analysis, this led to a larger number of articles than the original search. A possible explanation for this might be that goal-oriented care was covered by synonyms or similar concepts that were not covered by the original search. Despite the systematic approach, a concept analysis does not comprise a quality assessment of the literature. However, it seemed to be an appropriate method to provide the knowledge needed to understand the different components of goal-oriented care in its entirety. The literature that was included in this study were only English written and peer reviewed. It would however be interesting to add also non-English literature to be able to capture more differences (e.g. cultural differences).

The literature search identified both original research papers and position papers. Some original research papers [3, 4, 28, 43, 46] evaluated goal-oriented care in clinical practice. These papers identified and described goal-oriented care as a stepwise intervention. Position papers [1, 12, 13, 40, 42] mostly described components of goal-oriented care rather than such a stepwise approach. The combination of both types gave more insight in the broad components of goal-oriented care.

This concept analysis could also be considered as a preliminary step to facilitate further research. One of the knowledge gaps revealed in this concept analysis is the lack of knowledge on what patients' goals are set, how goal-oriented care is delivered, and how it is best put into practice in both one-on-one interactions between patients and providers and in interprofessional collaboration. Regarding patients it is important to gain more insight in how they are preferably prepared for discussing their personal goals. In addition, the list of empirical referents made clear that a golden standard to evaluate goal-oriented care is missing. Initiating the development of an evaluation method could enable future intervention studies to gain more insight in the consequences of goal-oriented care and to make results comparable. Increasing insights from effective goal-oriented care could highlight its multiple benefits towards providers and policy makers. These results might also inform the healthcare system in which resources they need to facilitate goal-oriented care. A following step will first be to discuss these theoretical insights with patients and providers and deepen this information with insights from practices. Then, when goal-oriented care is well understood, a critical review can be set up to perform in-depth comparison between other concepts and frameworks. At this moment, we have (unfortunately) insufficient information to do this.

Goal-oriented care shows the potential to be a way forward for patients with chronic conditions and multimorbidity. However, further research is needed to translate the current knowledge on the concept of goal-oriented care into a tangible workflow process of care that entails the three stages. This workflow should consists of tools to prepare patients and providers to offer goal-oriented care. This could contribute to finding a common ground in the goals and implementing goal-oriented care in practice.

Conclusion

This concept analysis aimed to translate the concept of goal-oriented care into a common understanding so providers can better understand and use this concept in clinical practice. The various literature on goal-oriented care, based on position and original research papers, showed a stepwise approach of three stages. Overall, the underpinning attributes of patients' context and patients' values form a philosophy of care to which the process must be reflected. Furthermore, both patients and the providers need to develop new skills in order to rethink the way care is provided. Patients must therefore be enabled to open up and reflect on their own agenda. Providers instead must learn to let go their own assumptions and solutions and communicate with their patients in a more balanced context. Based on the literature goal-oriented care shows the potential to improve patients' experience by listening to their needs and preferences, improve providers' well-being by the feeling of more satisfaction and reduce health care costs. Goal-oriented care could answer the challenges patients face with multiple care processes by initiating interprofessional collaboration. However, further research must focus on what and how goals are set, the translation of these findings into a workflow and must initiate the development of an evaluation method in order to investigate the effects of goal-oriented care processes on patients, providers and the health care system.

Acknowledgments

We are grateful for the partnership with the Primary Care Academy (academie-eerstelijn.be) and want to thank the King Baudouin Foundation and Fund Daniel De Coninck for the opportunity they offer us for conducting research and have impact on the primary care of Flanders, Belgium. The consortium of the Primary Care Academy consists of: Lead author: Roy Remmen – roy.remmen@uantwerpen.be - Department of Primary Care and Interdisciplinary Care, Faculty of Medicine and Health Sciences. University of Antwerp. Antwerp. Belgium; Emily Verté - Department of Primary Care and Interdisciplinary Care, Faculty of Medicine and Health Sciences. University of Antwerp. Antwerp.

Belgium, Department of Family Medicine and Chronic Care, Faculty of Medicine and Pharmacy. Vrije Universiteit Brussel. Brussel. Belgium; Muhammed Mustafa Sirimsi - Centre for research and innovation in care, Faculty of Medicine and Health Sciences. University of Antwerp. Antwerp. Belgium; Peter Van Bogaert - Workforce Management and Outcomes Research in Care, Faculty of Medicine and Health Sciences. University of Antwerp. Belgium; Hans De Loof - Laboratory of Physio pharmacology, Faculty of Pharmaceutical Biomedical and Veterinary Sciences. University of Antwerp. Belgium; Kris Van den Broeck - Department of Primary Care and Interdisciplinary Care, Faculty of Medicine and Health Sciences. University of Antwerp. Antwerp. Belgium.; Sibyl Anthierens - Department of Primary Care and Interdisciplinary Care, Faculty of Medicine and Health Sciences. University of Antwerp. Antwerp. Belgium; Ine Huybrechts - Department of Primary Care and Interdisciplinary Care, Faculty of Medicine and Health Sciences. University of Antwerp. Antwerp. Belgium.; Peter Raeymaeckers - Department of Sociology, Faculty of Social Sciences, Faculty of Social Sciences. University of Antwerp. Belgium; Veerle Buffel- Department of Sociology; centre for population, family and health, Faculty of Social Sciences. University of Antwerp. Belgium. ; Dirk Devroey- Department of Family Medicine and Chronic Care, Faculty of Medicine and Pharmacy. Vrije Universiteit Brussel. Brussel.; Bert Aertgeerts - Academic Centre for General Practice, Faculty of Medicine. KU Leuven. Leuven, Department of Public Health and Primary Care, Faculty of Medicine, KU Leuven. Leuven; Birgitte Schoenmakers - Department of Public Health and Primary Care, Faculty of Medicine, KU Leuven. Leuven. Belgium; Lotte Timmermans - Department of Public Health and Primary Care, Faculty of Medicine, KU Leuven. Leuven. Belgium.; Veerle Foulon - Department of Pharmaceutical and Pharmacological Sciences, Faculty Pharmaceutical Sciences. KU Leuven. Leuven. Belgium.; Anja Declerq - LUCAS-Centre for Care Research and Consultancy, Faculty of Social Sciences. KU Leuven. Leuven. Belgium.; Nick Verhaeghe - Research Group Social and Economic Policy and Social Inclusion, Research Institute for Work and Society. KU Leuven. Belgium.; Dominique Van de Velde Department of Rehabilitation Sciences, Occupational Therapy. Faculty of Medicine and Health Sciences. University of Ghent. Belgium., Department of Occupational Therapy. Artevelde University of

Applied Sciences. Ghent. Belgium.; Pauline Boeckxstaens - Department of Public Health and Primary Care, Faculty of Medicine and Health sciences. University of Ghent. Belgium.; An De Sutter - Department of Public Health and Primary Care, Faculty of Medicine and Health sciences. University of Ghent. Belgium.; Patricia De Vriendt - Department of Rehabilitation Sciences, Occupational Therapy. Faculty of Medicine and Health Sciences. University of Ghent. Belgium., Frailty in Ageing (FRIA) Research Group, Department of Gerontology and Mental Health and Wellbeing (MENT) research group, Faculty of Medicine and Pharmacy. Vrije Universiteit. Brussels. Belgium., Department of Occupational Therapy. Artevelde University of Applied Sciences. Ghent. Belgium.; Lies Lahousse - Department of Bioanalysis, Faculty of Pharmaceutical Sciences, Ghent University. Ghent. Belgium.; Peter Pype - Department of Public Health and Primary Care, Faculty of Medicine and Health sciences. University of Ghent. Belgium., End-of-Life Care Research Group, Faculty of Medicine and Health Sciences. Vrije Universiteit Brussel and Ghent University. Ghent. Belgium.; Dagje Boeykens- Department of Rehabilitation Sciences, Occupational Therapy. Faculty of Medicine and Health Sciences. University of Ghent. Belgium., Department of Public Health and Primary Care, Faculty of Medicine and Health sciences. University of Ghent. Belgium.; Ann Van Hecke - Department of Public Health and Primary Care, Faculty of Medicine and Health sciences. University of Ghent. Belgium., University Centre of Nursing and Midwifery, Faculty of Medicine and Health Sciences. University of Ghent. Belgium.; Peter Decat - Department of Public Health and Primary Care, Faculty of Medicine and Health sciences. University of Ghent. Belgium.; Rudi Roose - Department of Social Work and Social Pedagogy, Faculty of Psychology and Educational Sciences. University Ghent. Belgium.; Sandra Martin - Expertise Centre Health Innovation. University College Leuven-Limburg. Leuven. Belgium.; Erica Rutten - Expertise Centre Health Innovation. University College Leuven-Limburg. Leuven. Belgium.; Sam Pless - Expertise Centre Health Innovation. University College Leuven-Limburg. Leuven. Belgium.; Vanessa Gauwe - Department of Occupational Therapy. Artevelde University of Applied Sciences. Ghent. Belgium.; Didier Reynaert- E-QUAL, University College of Applied Sciences Ghent. Ghent. Belgium.; Leen Van Landschoot - Department of Nursing, University of Applied Sciences Ghent. Ghent.

Belgium.; Maja Lopez Hartmann - Department of Welfare and Health, Karel de Grote University of Applied Sciences and Arts. Antwerp. Belgium.; Tony Claeys- LiveLab, VIVES University of Applied Sciences. Kortrijk. Belgium.; Hilde Vandenhoudt - LiCalab, Thomas University of Applied Sciences. Turnhout. Belgium.; Kristel De Vliegher - Department of Nursing – homecare, White-Yellow Cross. Brussels. Belgium.; Susanne Op de Beeck - Flemish Patient Platform. Heverlee. Belgium.

References

1. Tinetti ME, Esterson J, Ferris R, Posner P, Blaum CS. Patient Priority-Directed Decision Making and Care for Older Adults with Multiple Chronic Conditions. Clinics in geriatric medicine. 2016;32(2):261-75. PubMed PMID: 27113145.
2. Rijken M, Bekkema N, Boeckxstaens P, Schellevis FG, De Maeseneer JM, Groenewegen PP. Chronic Disease Management Programmes: an adequate response to patients' needs? Health Expect. 2014;17(5):608-21. Epub 2012/06/21. doi: 10.1111/j.1369-7625.2012.00786.x. PubMed PMID: 22712877; PubMed Central PMCID: PMCPMC5060914.
3. Feder SL, Kiwak E, Costello D, Dindo L, Hern, ez-Bigos K, et al. Perspectives of Patients in Identifying Their Values-Based Health Priorities. Journal of the American Geriatrics Society. 2019 67(7):1379-85. PubMed PMID: 30844080.
4. Naik AD, Dindo LN, Van Liew JR, Hundt NE, Vo L, Hern, et al. Development of a Clinically Feasible Process for Identifying Individual Health Priorities. Journal of the American Geriatrics Society. 2018;66(10):1872-9. PubMed PMID: rayyan-47069467.
5. Cassell A, Edwards D, Harshfield A, Rhodes K, Brimicombe J, Payne R, et al. The epidemiology of multimorbidity in primary care: a retrospective cohort study. Br J Gen Pract. 2018;68(669):e245-e51. Epub 2018/03/14. doi: 10.3399/bjgp18X695465. PubMed PMID: 29530918; PubMed Central PMCID: PMCPMC5863678.
6. Berntsen G, Hoyem A, Lettrem I, Ruland C, Rumpsfeld M, Gammon D. A person-centered integrated care quality framework, based on a qualitative study of patients' evaluation of care in light of chronic care ideals. BMC Health Serv Res. 2018;18(1):479. Epub 2018/06/22. doi: 10.1186/s12913-018-3246-z. PubMed PMID: 29925357; PubMed Central PMCID: PMCPMC6011266.
7. Ploeg J, Matthew-Maich N, Fraser K, Dufour S, McAiney C, Kaasalainen S, et al. Managing multiple chronic conditions in the community: a Canadian qualitative study of the experiences of older adults, family caregivers and healthcare providers. BMC Geriatr. 2017;17(1):40.
8. Kramer MH, Bauer W, Dicker D, Durusu-Tanriover M, Ferreira F, Rigby SP, et al. The changing face of internal medicine: patient centred care. European journal of internal medicine. 25(2):125-7. PubMed PMID: 24472695.
9. Kuluski K, Peckham A, Gill A, Gagnon D, Wong-Cornall C, McKillop A, et al. What is Important to Older People with Multimorbidity and Their Caregivers? Identifying Attributes of Person Centered Care from the User Perspective. Int J Integr Care. 2019;19(3):4. Epub 2019/08/02. doi: 10.5334/ijic.4655. PubMed PMID: 31367203; PubMed Central PMCID: PMCPMC6659759.
10. Reuben DB, Tinetti ME. Goal-oriented patient care--an alternative health outcomes paradigm. The New England journal of medicine. 2012;366(9):777-9. Epub 2012/03/02. doi: 10.1056/NEJMp1113631. PubMed PMID: 22375966.
11. Kuipers SJ, Cramm JM, Nieboer AP. The importance of patient-centered care and co-creation of care for satisfaction with care and physical and social well-being of patients with multi-morbidity in the primary care setting. BMC Health Serv Res. 2019;19(1):1-9.
12. Reuben DB, Jennings LA. Putting Goal-Oriented Patient Care Into Practice. Journal of the American Geriatrics Society. 2019;67(7):1342-4. PubMed PMID: 30882888.
13. Mold JW, Blake GH, Becker LA. Goal-oriented medical care. Family medicine. 1991;23(1):46-51. Epub 1991/01/01. PubMed PMID: 2001782.
14. Sackett DL, Rosenberg WM, Gray JM, Haynes RB, Richardson WS. Evidence based medicine: what it is and what it isn't. British Medical Journal Publishing Group; 1996.
15. Baker A. Crossing the quality chasm: a new health system for the 21st century: British Medical Journal Publishing Group; 2001.

16. Elwyn G, Frosch D, Thomson R, Joseph-Williams N, Lloyd A, Kinnersley P, et al. Shared decision making: a model for clinical practice. Journal of general internal medicine. 2012;27(10):1361-7.
17. Vermunt N, Harmsen M, Westert GP, Olde Rikkert MGM, Faber MJ. Collaborative goal setting with elderly patients with chronic disease or multimorbidity: a systematic review. BMC Geriatr. 2017;17(1):167. Epub 2017/08/02. doi: 10.1186/s12877-017-0534-0. PubMed PMID: 28760149; PubMed Central PMCID: PMCPMC5537926.
18. Poitras ME, Maltais ME, Bestard-Denomme L, Stewart M, Fortin M. What are the effective elements in patient-centered and multimorbidity care? A scoping review. BMC Health Serv Res. 2018;18(1):446. Epub 2018/06/15. doi: 10.1186/s12913-018-3213-8. PubMed PMID: 29898713; PubMed Central PMCID: PMCPMC6001147.
19. Hobbs FR, Bankhead C, Mukhtar T, Stevens S, Perera-Salazar R, Holt T, et al. Clinical workload in UK primary care: a retrospective analysis of 100 million consultations in England, 2007–14. The Lancet. 2016;387(10035):2323-30.
21. Institute of Medicine Committee on the Future of Primary C. In: Donaldson M, Yordy K, Vanselow N, editors. Defining Primary Care: An Interim Report. Washington (DC): National Academies Press (US) Copyright 1994 by the National Academy of Sciences.; 1994.
22. Ellner AL, Phillips RS. The Coming Primary Care Revolution. J Gen Intern Med. 2017;32(4):380-6. Epub 2017/03/01. doi: 10.1007/s11606-016-3944-3. PubMed PMID: 28243869; PubMed Central PMCID: PMCPMC5377886.
23. Elwyn G, Durand MA, Song J, Aarts J, Barr PJ, Berger Z, et al. A three-talk model for shared decision making: multistage consultation process. bmj. 2017;359:j4891.
24. Lusk JM, Fater K. A concept analysis of patient-centered care. Nurs Forum. 2013;48(2):89-98. Epub 2013/04/23. doi: 10.1111/nuf.12019. PubMed PMID: 23600637.
25. Walker LOA, K.C. Strategies for theory constructing in nursing. Texas2019.
26. Levac D, Colquhoun H, O'Brien KK. Scoping studies: advancing the methodology. Implement Sci. 2010;5:69. Epub 2010/09/22. doi: 10.1186/1748-5908-5-69. PubMed PMID: 20854677; PubMed Central PMCID: PMCPMC2954944.
27. Mourad Ouzzani HH, Zbys Fedorowicz, and Ahmed Elmagarmid. Rayyan - a web and mobile app for systematic reviews. . Systematic Reviews. 2016;5(210). doi: 10.1186/s13643-016-0384-4.
28. Salter C, Shiner A, Lenaghan E, Murdoch J, Ford JA, Winterburn S, et al. Setting goals with patients living with multimorbidity: qualitative analysis of general practice consultations. Br J Gen Pract. 2019;69(684):e479-e88. Epub 2019/06/05. doi: 10.3399/bjgp19X704129. PubMed PMID: 31160370; PubMed Central PMCID: PMCPMC6592350.
29. Kessler D, Walker I, Sauve-Schenk K, Egan M. Goal setting dynamics that facilitate or impede a client-centered approach. Scandinavian journal of occupational therapy. 2018;26(5):315-24. PubMed PMID: 29671662.
30. De Maeseneer J, Boeckxstaens P. Care for noncommunicable diseases (NCDs): time for a paradigm-shift. World hospitals and health services : the official journal of the International Hospital Federation. 2011;47(4):30-3. PubMed PMID: 22619872.
31. Bayliss EA, Bonds DE, Boyd CM, Davis MM, Finke B, Fox MH, et al. Understanding the context of health for persons with multiple chronic conditions: moving from what is the matter to what matters. Ann Fam Med. 2014;12(3):260-9. Epub 2014/05/14. doi: 10.1370/afm.1643. PubMed PMID: 24821898; PubMed Central PMCID: PMCPMC4018375.
32. Berntsen GK, Gammon D, Steinsbekk A, Salamonsen A, Foss N, Ruland C, et al. How do we deal with multiple goals for care within an individual patient trajectory? A document content analysis of health service research papers on goals for care. BMJ open. 2015;5(12):e009403. Epub 2015/12/15. doi: 10.1136/bmjopen-2015-009403. PubMed PMID: 26656243; PubMed Central PMCID: PMCPMC4679896.
33. Blom J, Elzen WD, Houwelingen Anne HV, Heijmans M, Stijnen T, Van Den Hout W, et al. Effectiveness and cost-effectiveness of a proactive, goal-oriented, integrated care model in general

practice for older people. A cluster randomised controlled trial: Integrated systematic care for older people-the ISCOPE study. Age and Ageing. 2016;45(1):30-41. doi: 10.1093/ageing/afv174.
34. Boeckxstaens, Willems, Lanssens, Decuypere, Brusselle, Kühlein, et al. A qualitative interpretation of challenges associated with helping patients with multiple chronic diseases identify their goals. Journal of comorbidity. 2016;6(2):120-6.
35. Mangin D, Stephen G, Bismah V, Risdon C. Making patient values visible in healthcare: a systematic review of tools to assess patient treatment priorities and preferences in the context of multimorbidity. BMJ open. 2016;6(6):e010903.
36. Schmidt K, Babac A, Pauer F, Damm K, von der Schulenburg JM. Measuring patients' priorities using the Analytic Hierarchy Process in comparison with Best-Worst-Scaling and rating cards: methodological aspects and ranking tasks. Health economics review. 2016;6(1):50. PubMed PMID: 27844450.
37. Franklin M, Lewis S, Willis K, Rogers A, Venville A, Smith L. Controlled, Constrained, or Flexible? How Self-Management Goals Are Shaped By Patient-Provider Interactions. Qualitative health research. 2019:1049732318774324. PubMed PMID: 29871583.
38. Tinetti M, Dindo L, Smith CD, Blaum C, Costello D, Ouellet G, et al. Challenges and strategies in patients' health priorities-aligned decision-making for older adults with multiple chronic conditions. PloS one. 2019;14(6):e0218249. PubMed PMID: 31181117.
39. Hurn J, Kneebone I, Cropley M. Goal setting as an outcome measure: A systematic review. Clinical rehabilitation. 2006;20(9):756-72. Epub 2006/09/29. doi: 10.1177/0269215506070793. PubMed PMID: 17005500.
40. Bodenheimer T, Handley MA. Goal-setting for behavior change in primary care: an exploration and status report. Patient education and counseling. 2009;76(2):174-80. Epub 2009/06/30. doi: 10.1016/j.pec.2009.06.001. PubMed PMID: 19560895.
41. Junius-Walker U, Stolberg D, Steinke P, Theile G, Hummers-Pradier E, Dierks M-L. Health and treatment priorities of older patients and their general practitioners: a cross-sectional study. Quality in primary care. 2011;19(2).
42. Lenzen SA, Daniels R, van Bokhoven MA, van der Weijden T, Beurskens A. Setting goals in chronic care: Shared decision making as self-management support by the family physician. The European journal of general practice. 2015;21(2):138-44. Epub 2014/12/30. doi: 10.3109/13814788.2014.973844. PubMed PMID: 25541857.
43. Steele Gray C, Wodchis WP, Upshur R, Cott C, McKinstry B, Mercer S, et al. Supporting Goal-Oriented Primary Health Care for Seniors with Complex Care Needs Using Mobile Technology: Evaluation and Implementation of the Health System Performance Research Network, Bridgepoint Electronic Patient Reported Outcome Tool. JMIR research protocols. 2016;5(2):e126. PubMed PMID: 27341765.
44. Kangovi S, Mitra N, Smith RA, Kulkarni R, Turr L, Huo H, et al. Decision-making and goal-setting in chronic disease management: Baseline findings of a randomized controlled trial. Patient Educ Couns. 2017;100(3):449-55. Epub 2016/10/09. doi: 10.1016/j.pec.2016.09.019. PubMed PMID: 27717532; PubMed Central PMCID: PMCPMC5437864.
45. Mold. Goal-directed health care: redefining health and health care in the era of value-based care. Cureus 2017;9(2).
46. Schellinger SE, Anderson EW, Frazer MS, Cain CL. Patient Self-Defined Goals: Essentials of Person-Centered Care for Serious Illness. The American journal of hospice & palliative care. 2018;35(1):159-65. Epub 2017/03/24. doi: 10.1177/1049909117699600. PubMed PMID: 28330379; PubMed Central PMCID: PMCPMC5704564.
47. Vermunt NP, Harmsen M, Elwyn G, Westert GP, Burgers JS, Olde Rikkert MG, et al. A three-goal model for patients with multimorbidity: A qualitative approach. Health expectations : an international journal of public participation in health care and health policy. 2018;21(2):528-38. PubMed PMID: 29193557.
48. de Groot E, Schonrock-Adema J, Zwart D, Damoiseaux R, Van den Bogerd K, Diemers A, et al. Learning from patients about patient-centredness: A realist review: BEME Guide No. 60. Medical

teacher. 2019:1-13. Epub 2019/12/20. doi: 10.1080/0142159x.2019.1695767. PubMed PMID: 31852313.
49. Kuluski K, Guilcher SJT. Toward a Person-Centred Learning Health System: Understanding Value from the Perspectives of Patients and Caregivers. HealthcarePapers. 2019;18(4):36-46. Epub 2020/01/05. doi: 10.12927/hcpap.2019.26030. PubMed PMID: 31901067.
50. Tinetti ME, Naik AD, Dindo L, Costello DM, Esterson J, Geda M, et al. Association of Patient Priorities-Aligned Decision-Making with Patient Outcomes and Ambulatory Health Care Burden among Older Adults with Multiple Chronic Conditions: A Nonrandomized Clinical Trial. JAMA Internal Medicine. 2019. PubMed PMID: rayyan-47069570.
51. Eckhoff DO, Weiss J. Goal setting: A concept analysis. Nursing forum. 2020. Epub 2020/01/07. doi: 10.1111/nuf.12426. PubMed PMID: 31902133.
53. Sathanapally H, Sidhu M, Fahami R, Gillies C, Kadam U, Davies MJ, et al. Priorities of patients with multimorbidity and of clinicians regarding treatment and health outcomes: a systematic mixed studies review. BMJ open. 2020;10(2):e033445. Epub 2020/02/14. doi: 10.1136/bmjopen-2019-033445. PubMed PMID: 32051314.
54. Reuben DB, Tinetti ME. Goal-oriented patient care - An alternative health outcomes paradigm. New England Journal of Medicine. 2012;366(9):777-9. doi: 10.1056/NEJMp1113631.
55. Heisler M, Bouknight RR, Hayward RA, Smith DM, Kerr EA. The relative importance of physician communication, participatory decision making, and patient understanding in diabetes self-management. Journal of general internal medicine. 2002;17(4):243-52.
56. Purkaple BA, Nagykaldi ZJ, Allahyar A, Todd R, Mold JW. Physicians' Response to Patients' Quality-of-Life Goals. J Am Board Fam Med. 2020;33(1):71-9. Epub 2020/01/08. doi: 10.3122/jabfm.2020.01.190169. PubMed PMID: 31907248.
57. Boyd CM, Darer J, Boult C, Fried LP, Boult L, Wu AW. Clinical practice guidelines and quality of care for older patients with multiple comorbid diseases: implications for pay for performance. Jama. 2005;294(6):716-24. Epub 2005/08/11. doi: 10.1001/jama.294.6.716. PubMed PMID: 16091574.
58. Mangin D, Stephen G, Bismah V, Risdon C. Making patient values visible in healthcare: A systematic review of tools to assess patient treatment priorities and preferences in the context of multimorbidity. BMJ Open. 2016;6(6). doi: 10.1136/bmjopen-2015-010903.
59. Vermunt NP, Harmsen M, Westert GP, Rikkert MGO, Faber MJ. Collaborative goal setting with elderly patients with chronic disease or multimorbidity: a systematic review. BMC geriatrics. 2017;17(1):167.
60. Boyd CM, Wolff JL, Giovannetti E, Reider L, Weiss C, Xue Q-l, et al. Health care task difficulty among older adults with multimorbidity. Medical care. 2014;52(0 3):S118.
61. Locke EA, Latham GP. A theory of goal setting & task performance: Prentice-Hall, Inc; 1990.
62. Callahan EJ, Bertakis KD. Development and validation of the Davis Observation Code. Fam Med. 1991;23(1):19-24. Epub 1991/01/01. PubMed PMID: 2001776.
63. Åsenlöf P, Siljebäck K. The patient goal priority questionnaire is moderately reproducible in people with persistent musculoskeletal pain. Physical therapy. 2009;89(11):1226-34.
64. Melville LL, Baltic TA, Bettcher TW, Nelson DL. Patients' perspectives on the self-identified goals assessment. Am J Occup Ther. 2002;56(6):650-9. Epub 2002/12/03. doi: 10.5014/ajot.56.6.650. PubMed PMID: 12458857.
65. Dedding C, Cardol M, Eyssen IC, Dekker J, Beelen A. Validity of the Canadian Occupational Performance Measure: a client-centred outcome measurement. Clinical rehabilitation. 2004;18(6):660-7. Epub 2004/10/12. doi: 10.1191/0269215504cr746oa. PubMed PMID: 15473118.
66. Fried TR, Tinetti M, Agostini J, Iannone L, Towle V. Health outcome prioritization to elicit preferences of older persons with multiple health conditions. Patient education and counseling. 2011;83(2):278-82.
67. Steele Gray C, Gill A, Khan AI, Hans PK, Kuluski K, Cott C. The Electronic Patient Reported Outcome Tool: Testing Usability and Feasibility of a Mobile App and Portal to Support Care for Patients With Complex Chronic Disease and Disability in Primary Care Settings. JMIR Mhealth

Uhealth. 2016;4(2):e58. Epub 2016/06/04. doi: 10.2196/mhealth.5331. PubMed PMID: 27256035; PubMed Central PMCID: PMCPMC4911509.
68. Toto PE, Skidmore ER, Terhorst L, Rosen J, Weiner DK. Goal Attainment Scaling (GAS) in geriatric primary care: a feasibility study. Arch Gerontol Geriatr. 2015;60(1):16-21. Epub 2014/12/04. doi: 10.1016/j.archger.2014.10.022. PubMed PMID: 25465505.
69. Glasgow RE, Wagner EH, Schaefer J, Mahoney LD, Reid RJ, Greene SM. Development and validation of the Patient Assessment of Chronic Illness Care (PACIC). Med Care. 2005;43(5):436-44. Epub 2005/04/20. doi: 10.1097/01.mlr.0000160375.47920.8c. PubMed PMID: 15838407.
70. Teal CR, Haidet P, Balasubramanyam AS, Rodriguez E, Naik AD. Measuring the quality of patients' goals and action plans: development and validation of a novel tool. BMC medical informatics and decision making. 2012;12:152. Epub 2012/12/29. doi: 10.1186/1472-6947-12-152. PubMed PMID: 23270422; PubMed Central PMCID: PMCPMC3544573.
71. Etz RS, Zyzanski SJ, Gonzalez MM, Reves SR, O'Neal JP, Stange KC. A New Comprehensive Measure of High-Value Aspects of Primary Care. Annals of family medicine. 2019;17(3):221-30. PubMed PMID: rayyan-47069321.
72. Adam P, Murphy CF, Dierich M, Hager KD. Seven Years of Teaching Communication With the Patient-Centered Observation Form. Fam Med. 2018;50(2):132-7. Epub 2018/02/13. doi: 10.22454/FamMed.2018.516713. PubMed PMID: 29432629.
73. Murdoch J, Salter C, Ford J, Lenaghan E, Shiner A, Steel N. The "unknown territory" of goal-setting: Negotiating a novel interactional activity within primary care doctor-patient consultations for patients with multiple chronic conditions. Soc Sci Med. 2020;256:113040. Epub 2020/05/31. doi: 10.1016/j.socscimed.2020.113040. PubMed PMID: 32473530; PubMed Central PMCID: PMCPMC7306159.
74. Singh Ospina N, Phillips KA, Rodriguez-Gutierrez R, Castaneda-Guarderas A, Gionfriddo MR, Branda ME, et al. Eliciting the Patient's Agenda- Secondary Analysis of Recorded Clinical Encounters. J Gen Intern Med. 2019;34(1):36-40. Epub 2018/07/04. doi: 10.1007/s11606-018-4540-5. PubMed PMID: 29968051; PubMed Central PMCID: PMCPMC6318197.
75. Hersh L, Salzman B, Snyderman D. Health Literacy in Primary Care Practice. Am Fam Physician. 2015;92(2):118-24. Epub 2015/07/16. PubMed PMID: 26176370.
76. Sine DM, Sharpe VA. Ethics, risk, and patient-centered care: how collaboration between clinical ethicists and risk management leads to respectful patient care. J Healthc Risk Manag. 2011;31(1):32-7. Epub 2011/07/28. doi: 10.1002/jhrm.20077. PubMed PMID: 21793115.
77. Tinetti ME, Bogardus Jr ST, Agostini JV. Potential pitfalls of disease-specific guidelines for patients with multiple conditions. N Engl j Med. 2004;351(27):2870-4.
78. Voigt I, Wrede J, Diederichs-Egidi H, Dierks ML, Junius-Walker U. Priority setting in general practice: health priorities of older patients differ from treatment priorities of their physicians. Croatian medical journal. 2010;51(6):483-92. Epub 2010/12/17. doi: 10.3325/cmj.2010.51.483. PubMed PMID: 21162160; PubMed Central PMCID: PMCPMC3012393.
79. Etz R, Stange KC. Measuring what matters in primary care. Global Advances in Health and Medicine. 2018;7:263. doi: 10.1177/2164956118773837.
80. Young RA, Roberts RG, Holden RJ. The Challenges of Measuring, Improving, and Reporting Quality in Primary Care. Annals of family medicine. 2017;15(2):175-82. Epub 2017/03/16. doi: 10.1370/afm.2014. PubMed PMID: 28289120; PubMed Central PMCID: PMCPMC5348238.

Supporting information

S1 Table 1. Overview preliminary version attributes.

S2 Table 2. Overview training.

S1 File. Prisma scr checklist.

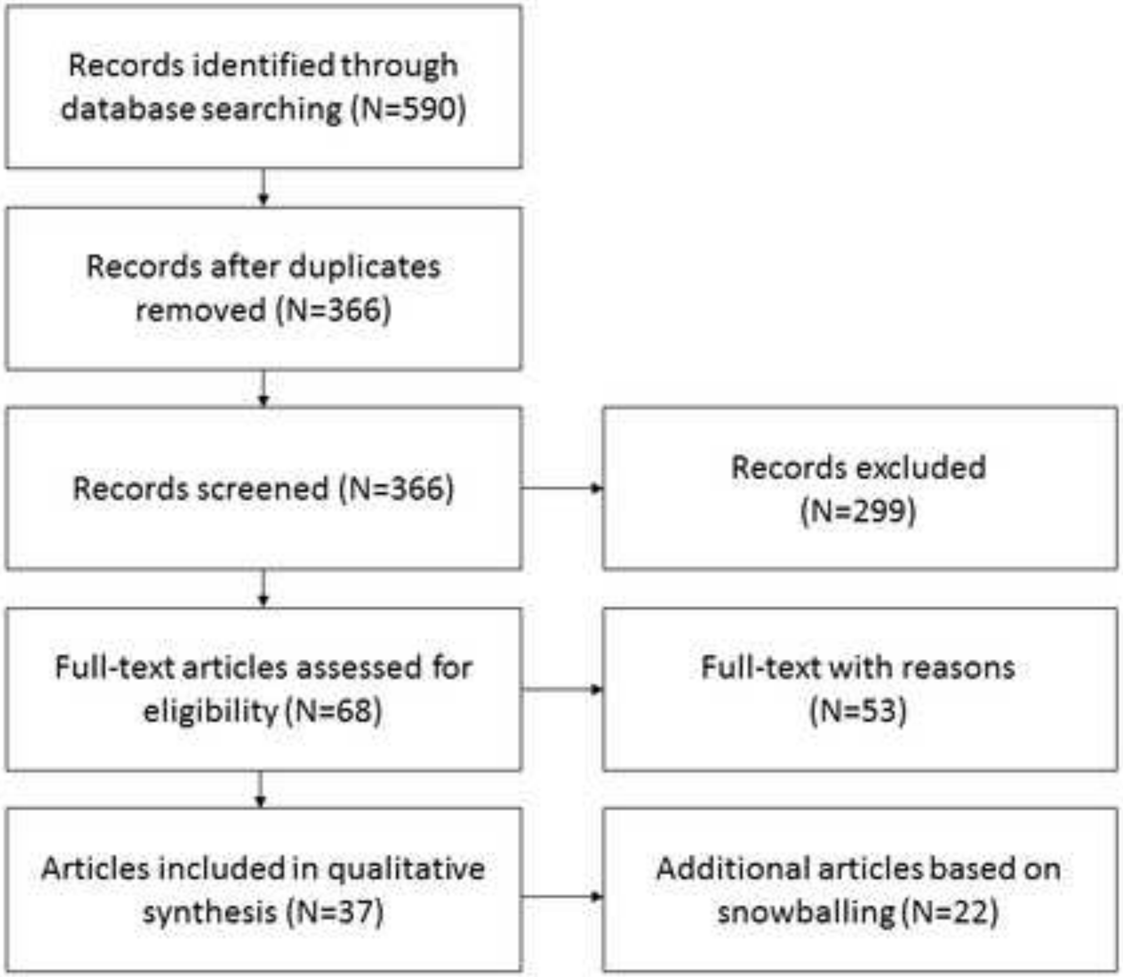

Figure 1 Flow chart demonstrating the search string

Figure 2

Click here to access/download;Figure;Fig 2.tif

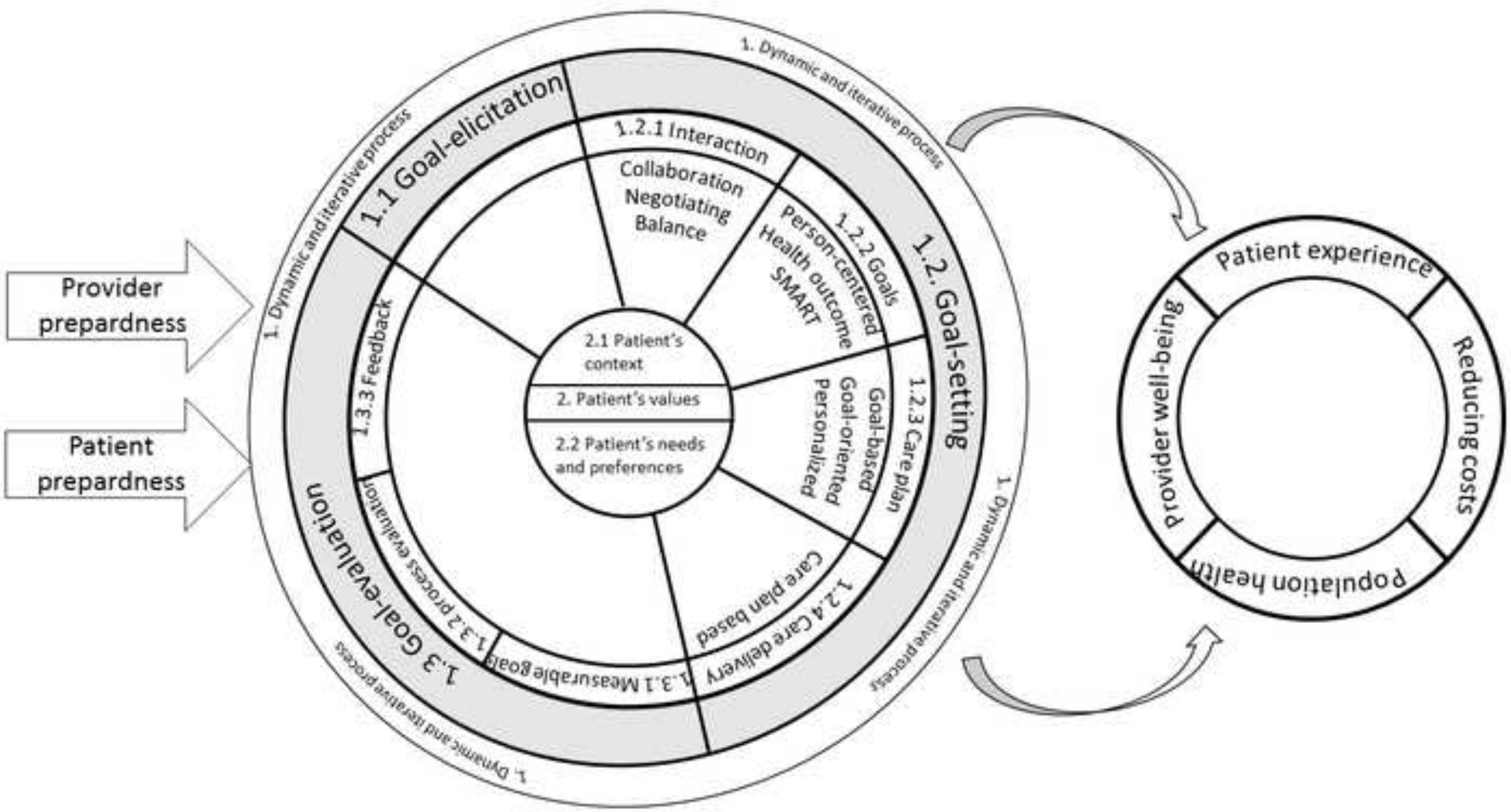

Supporting Information Table 1

Click here to access/download
Supporting Information
S1 Table 1.pdf

Supporting Information Table 2

Click here to access/download
Supporting Information
S2 Table2.pdf

Supporting Information File 1

Click here to access/download
Supporting Information
S1 File.pdf

Revised Manuscript with Track Changes

Goal-oriented care for patients with chronic conditions or multimorbidity in primary care: a scoping review and concept analysis.

Dagje Boeykens [12¶*], Pauline Boeckxstaens [2¶], An De Sutter [2], Lies Lahousse [3], Peter Pype [245], Patricia De Vriendt [156&], Dominique Van de Velde[15&], on behalf of the Primary Care Academy[^].

1. Department of Rehabilitation Sciences, Occupational Therapy. Faculty of Medicine and Health Sciences. Ghent University. Ghent. Belgium.
2. Department of Public Health and Primary Care, Faculty of Medicine and Health Sciences. Ghent University. Ghent. Belgium.
3. Department of Bioanalysis, Faculty of Pharmaceutical Sciences, Ghent University. Ghent. Belgium.
4. End-of-Life Care Research Group, Faculty of Medicine and Health Sciences. Vrije Universiteit BrusselUB and Ghent University. Ghent. Belgium.
5. Department of Occupational Therapy. Artevelde University College. Ghent, Belgium.
6. Department of Gerontology and Frailty in Ageing Research Group, Faculty of Medicine and Pharmacy. Vrije Universiteit Brussel. Brussels. Belgium.
6. Frailty in Ageing (FRIA) Research Group, Department of Gerontology and Mental Health and Wellbeing (MENT) research group, Faculty of Medicine and Pharmacy. Vrije Universiteit Brussel. Brussel. Belgium.

* Corresponding author: Dagje.boeykens@ugent.be

¶: These authors contributed equally to this work.

&: These authors also contributed equally to this work.

^Membership of the Primary Care Academy is provided in the Acknowledgments.

Abstract

Background

The healthcare system is faced by an ageing population, increase in chronic conditions and multimorbidity. Multimorbid patients are faced with multiple parallel care processes leading to a risk of~~for~~ fragmented care. These problems relate to the disease-oriented paradigm. In this paradigm the treatment goals can be in contrast with what patients value.

The concept of goal-oriented care is proposed as an alternative way of providing care as meeting patients' goals could have potential benefits. Though, there ~~. There~~ is a need to translate this concept into tangible knowledge so providers can better understand and use the concept in clinical practice. The aim of this study is to address this need by means of a concept analysis.

Method

This concept analysis using the method of Walker and Avant is based on a literature search in PubMed, Embase, Cochrane Library, PsychInfo, CINAHL, OTSeeker and Web of Science. The method provides eight iterative steps: select a concept, determine purpose, determine defining attributes, identify model case, identify additional case, identify antecedents and consequences and define empirical referents.

Results

The analysis of 37 articles revealed that goal-oriented care is a dynamic and iterative process of three stages: goal-elicitation, goal-setting, and goal-evaluation. The process is underpinned by the patient's context and values. Provider and patient preparedness are required to provide goal-oriented care. Goal-oriented care has the potential to improve patients' experiences and providers' well-being, to reduce costs, and improve the overall population health. The challenge is to identify empirical referents to evaluate the process of goal-oriented care.

Conclusion

A common understanding of goal-oriented care is presented. Further research should focus on how and what goals are set by the patient, how this knowledge could be translated into a tangible workflow and should support the development of a strategy to evaluate the goal-oriented process of care.

Keywords

goal-oriented care, goal-setting, patient-centeredness, chronic conditions, multimorbidity, review, concept analysis

Introduction

The healthcare system is faced by an ageing population and an increase in chronic conditions and multimorbidity [1]. More and more people are forced to live with the consequences of these demographic changes and require ongoing (chronic) care on top of acute care [2]. At the same time, patient autonomy is gaining importance and patients are considered as an active and important partner in their care [3, 4]. Patients with chronic conditions are often consulting multiple health care providers [3] leading to a higher rate of encounters. They also receive a larger amount of prescriptions [5] and they are asked to complete a diverse set of self-monitoring tasks such as managing, exacerbations or monitoring biomedical targets [3]. Since patients with (multiple) chronic conditions are faced with multiple parallel care process for their different conditions, there is a considerable risk of~~for~~ fragmented care. Especially when health~~-~~care providers focus on disease control, patients can experience lack of care continuity and issues with communication as patients themselves focus on the meaning of care and more on personal wellbeing [6, 7]. As a result, treatment goals can be in contrast with what patients value in their personal lives [3].

The health~~-~~care system is oriented towards a disease-oriented paradigm to which many of these problems relate [8-10]. In this paradigm, care is mainly organized according to disease-oriented guidelines [10]. This may work well for patients with a single disease, but becomes inappropriate for patients with multiple problems. The focus on single disease guidelines might distract providers from what really matters to the patient [10]. A possible way to overcome many of the challenges is to shift care back from ‘what’s the matter with the patient’ to ‘what matters to the patient’. It creates health care processes in which patients’ needs are actively sought and met [9]. Meeting those patients’ needs and tailoring care more to what patients want in a co-creation process could result in better social well-being, physical well-being, and satisfaction for patients and healthcare providers [11].

One of the possible strategies is to actively engage patients in identifying their personal goals and aligning care to those goals, which could be achieved by goal-oriented care [12].

The concept of goal-oriented care has been launched and mentioned for the first time in 1991 by Mold who proposed the concept as an alternative way of providing care [13]. [4]Later on, in 2012, Reuben and Tinetti took the concept of goal-oriented care a step forward by stating that care "must above all consider patients' preferred outcomes" [10]. The focus on setting goals based on the patients' needs and preferences rather than on health-related outcomes became one of the main novelties in chronic disease management [4]. Not only could goal-oriented care be proposed as an important paradigm to overcome some of the new challenges for chronic patients [9], it might also corresponded to the original concept of evidence based medicine (EBM) [14]. EBM was first published by Sackett in 1996 who described three key components: 1. best external evidence, 2. individual clinical expertise, and 3. patients' values and expectations [14]. Since the first description of EBM, multiple approaches and paradigms has been developed to compromise between those three components [15]. For example, patient-centered care (PCC), which is already a well-known and widely used concept, is defined as "providing care that is respectful of, and responsive to individual patient preferences, needs, and values and ensuring that patients values guide all clinical decisions" [15]. Shared-decision making, on the other hand, also strives to share evidence and engage patients in care as it is "an approach where clinicians and patients share the best available evidence when faced with the task of making decisions, and where patients are supported to consider options, and to achieve informed preferences" [16]. Goal-oriented care is proposed as a promising healthcare paradigm and approach to operationalize EBM and return to where it all started [10]. However, in contrast to the other approaches and paradigms, goal-oriented care is ill defined. Developing a common understanding on the concept could potentially contribute to the clarification and in-depth comparison between the related concepts and eventually lead to better use in clinical practice. However, some healthcare providers might already assume that they practice goal-oriented care spontaneously, but there still is a lack of underpinning knowledge and guidance on how to provide goal-oriented care to patients. The main pitfall in most of these goal-setting activities is that the goals are not necessarily related to the patients' needs and preferences while in goal-oriented care these patients' needs and preferences are put on the forefront

and are not necessarily health-related. [17, 18]. From this perspective, goal-setting and goal-oriented care should be taken together and focus on the patients' needs and preferences.

As a first step in exploring the potential of goal-oriented care in chronic care, it is important to gain in-depth knowledge on what goal-oriented care is about and how it can be generally described.

As goal-oriented care could be well-suited in primary care, as this context is often the linchpin for patients with chronic conditions, this will be the focus of this study [19]. This study aimed to describe a structured approach to deepen the concept of goal-oriented care for patients with chronic conditions or multimorbidity in the primary care context. It has been suggested to contribute to patients' wellbeing and quality of life [17]. Goal-oriented care as a new paradigm of care has the potential to overcome some of the new challenges for chronical patients [9].

Primary care is often the linchpin of care for these patients [19]. It is easy accessible care in which providers address a large majority of health and social needs and develop sustained partnerships with patients in their community [20, 21]. Primary care offers a first contact point for new health needs, provides care continuity and care coordination in ongoing and complex cases [22]. The aim of this study is to address these knowledge gaps by means of a concept analysis to clarify the existing ambiguity and make an overview of the already existing knowledge. Clarity on the concept of goal-oriented care will enhance the understanding and will (potentially) facilitate the implementation of goal-oriented care interventions.

lthough many primary care providers assume they practice goal-oriented care spontaneously, there is a lack of underpinning knowledge and guidance on how to provide goal-oriented care to patients [17, 18]. There is an urgent need to translate the paradigm of goal-oriented care into tangible knowledge so providers can better understand and use this concept in clinical practice. The knowledge gap on goal-oriented care is not only characterized by a lack of in-depth knowledge of the concept. There are also related concepts (such as shared decision making [23] and patient centered care [24]) that challenge the common understanding of goal-oriented care.

~~The aim of this study is to address these knowledge gaps by means of a concept analysis to clarify the existing ambiguity and make an overview of the already existing knowledge. Clarity on the concept of goal-oriented care will enhance the understanding and will (potentially) facilitate the implementation of goal-oriented care interventions.~~

Method

This concept analysis aims to present an overview and synthetization of the existing literature regarding goal-oriented care for chronically ill patients in primary care. This will be performed by analyzing the concept into antecedents, attributes, and consequences following the method of Walker and Avant [25]. This method provides a framework of eight iterative steps: 1. select a concept, 2. determine the aims or purposes of analysis, 3. identify all concept definitions and select the literature, 4. determine different attributes, 5. identify a model case, 6. identify an additional case, 7. identify antecedents and consequences, and 8. define empirical referents [25]. In this concept analysis the attributes are the heart and will present the characteristics of goal-oriented care and allow the broadest insight into the concept [25].

Step 1: select a concept

Goal-oriented care has been defined as an underpinning strategy for primary care reform in Flanders, Belgium. The concept is presented as one of the main topics of 'The Primary Care Academy' (PCA). The PCA is a consortium consisting of four universities (Ghent University, University of Antwerp, Catholic university of Leuven, Vrije Universiteit of Brussels), six universities of applied sciences (UAC VIVES, UAC Artevelde, UAC Ghent, UAC Leuven-Limburg, UAC Karel de Grote, UAC Thomas More), and important stakeholders (Flemish Patient Platform and White-Yellow Cross; a home care organization) in Belgium with the aim to strengthen the primary care organization and delivery. The PCA includes experts in primary care from a variety of healthcare and welfare disciplines. Discussions in the research group working on goal-oriented care created a necessity to clarify the concept.

Step 2: determine the aims and purposes of the analysis

The aim of this concept analysis is to build a common understanding to eliminate ambiguity between the concepts related to goal-oriented care. Specifically, the scope of the concept analysis is to define goal-oriented care for people with chronic conditions at the level of primary care.

Step 3: select the literature

The literature search was conducted between January 2020 and April 2020. As the method of a concept analysis does not specify how the literature search has to be performed, this search was based on the method of a scoping review described by Levac (2010) [26]. A preliminary combination of search terms was identified: ‘goal-oriented care’, ‘chronic care’, and ‘primary care’. Based on these keywords a first search was performed to identify adjacent terms in the literature. The search strategy was revised in consultation with the librarian of the university and the senior researchers. The definitive keywords were: ‘goal-oriented care’, ‘goal-oriented medical care’, ‘person-centered goal-setting’, ‘patient-centered goal-setting’, ‘goal-oriented patient care’, and ‘patient priorities’, emphasized goal-oriented care and it synonyms. Related concepts such as patient-centered care, value-based care, etc. were not included as the method of concept analysis prescribes to deepen all the attributes of one concept. In a first phase, the keywords were entered in PubMed, Embase, and Cochrane Library (~~t~~Table 1). In a second phase, CINAHL, OTSeeker, PsycINFO, and Web of Science were consulted and confirmed the first results as no new studies were identified~~.~~

Table 1, Overview of the search strings.

PubMed

(goal-directed care[MeSH Terms]) OR goal-oriented care [Title/abstract]) OR goal-oriented medical care [Title/abstract]) OR person-centered goal-setting [Title/abstract]) OR patient centered goal-setting [Title/abstract]) OR goal-oriented patient care[Title/abstract]) OR patient priorities [Title/abstract])

Embase

‘**goal-oriented care’**:ab,ti OR ‘**goal-oriented medical care’**:ab,ti OR ‘**person-centered goal-setting’**:ab,ti OR ‘**patient centered goal-setting’**:ab,ti OR **‘goal-oriented patient care’**: ab,ti OR ‘**patient priorities’**:ab,ti

Cochrane

goal-oriented care in Title Abstract Keyword OR goal-oriented medical care in Title Abstract Keyword OR person-centered goal-setting in Title Abstract Keyword OR patient-centered goal-setting in Title Abstract Keyword OR goal-oriented patient care OR patient priorities in Title Abstract Keyword - (Word variations have been searched)

Articles resulting from this search were put in Rayyan [27] to administer the data. A first selection based on title and abstract was performed with regard to the predefined in- and exclusion criteria. Inclusion criteria: (a) goal-oriented care as a health-related concept, (b) mentioning goal-setting, goal-oriented care or related concept (e.g. person-centered integrated care), and (c) focusing on patients with one or more chronic conditions ~~a chronic condition or multimorbidity~~. Exclusion criteria: (a) focusing on single-disease management, (b) goals regarding disease-specific outcomes (e.g. cancer or diabetes), ~~and~~ (c) focusing on goal-oriented care in a specific context (e.g. rehabilitation center), and (d) specifically mentioning patient-centered care, shared-decision making, etc. as they will hamper the understanding of specifically goal-oriented care. Articles resulting from this first search were subjected to a full text screening based on the initial criteria and: (a) full text available, (b) written in English, (c)

Formatted: English (United States)

Formatted: Left, None, Space After: 8 pt, Line spacing: Double, Pattern: Clear

referring to goal-oriented care or related concepts as a concept, and (d) containing information of a theoretical building of a definition. There was no restriction by study design to gain as much insight in goal-oriented care from different data sources.

Step 4: defining the attributes

The determination of the attributes started with a discussion of four key articles [1, 6, 28, 29] selected by the first author based on the divers approaches of goal-oriented care and presented to the research group. Similar to a qualitative, thematic analysis, the key articles were analyzed based on an open coding and then grouped into codes ~~These key articles were analyzed~~(Table 2 – example of data analysis). These codes were then presented to and discussed with the co-authors. In these discussion rounds, codes were translated into attributes. ~~, deconstructed into codes and discussed with the entire research group resulting in a first overview of attributes of goal-oriented care.~~ In a second phase, new articles were added and analyzed based on the same method as the key articles until all relevant literature (based on the inclusion criteria) was included. The different codes were put into NVIVO12 to synthesize the data and to initiate further discussion with the research group. This resulted in the final attributes (T~~t~~able 4). The method starting from reading the first article to defining the attributes wa~~i~~s characterized by an iterative process in which the attributes were reformulated until consensus with the research group was reached.

Table 2. Example of analysis process of the study of Bernsten et al. 2018.

**Extract from article**	**Code**	**Attribute**
…A professional and a personal goal clashes in a decision process regarding the discontinuation of a medication the informant had been using for years…	Negotiation goals between professionals and patients.	Goal-setting – patient-provider interaction
… However "What matters to you?" gave a richer and more immediate insight into areas threatened by health issues…	Patient centeredness	Tailoring to patients' needs and preferences

Formatted: English (United States)
Formatted: English (United States)
Formatted: English (United States)
Formatted: Caption, Keep with next
Formatted: English (United States)
Field Code Changed
Formatted: Font: Bold
Formatted Table

…Goal evaluation serves as feedback to all contributors in the seamless care process… The result should be documented and linked back to goal adjustment and learning for the next cycle…	Feedback to the care process	Goal-evaluation

StepTEP 5: identify a model case, a contrary case, and a borderline case IDENTIFY A MODEL CASES, A CONTRARY CASE AND A BORDERLINE CASE

A model case is presented as a narrative of how goal-oriented care could be conceptualized and illustrates all defined attributes of goal-oriented care [25]. A contrary and borderline case differ from this model case and do not include all of the attributes and/or differ in one of them.

Step 6: identify antecedents and consequences

Antecedents are events or incidents that precede the process of applying goal-oriented care. Consequences are those events or incidents as a result of applying goal-oriented care [25]. The antecedents and consequences were searched simultaneously with the attributes (step 4). Results have been discussed by the entire research group until consensus was reached.

Step 7: define empirical referents

Empirical referents provide an overview of the identified assessment tools related to the attributes aiming to make the concept, goal-oriented care, measurable. These assessment tools may be seen as the underpinning needs and characteristics when developing an evaluation method of goal-oriented care.

Formatted: Font: 16 pt, Bold

Formatted: Font: 16 pt, Bold

Formatted: Font: 16 pt, Bold

Results

Step 1-3

A first search based on the predefined terms (Table 1) resulted in 590 articles; 82 from Cochrane Library, 188 from Embase, and 313 from PubMed. After removing the duplicates, 366 articles were screened by title and abstract yielding 68 articles. A full text screening of these 68 articles lead to 15 articles that fitted the predefined in- and exclusion criteria (step 3). Based on the snowballing method of adding new articles based on references, citations, and similar articles 22 additional articles were added. This resulted in a total of 37 articles (Fig. 1 and Table 3) that were selected for the full text analysis. These articles represented a broad range of study types: 4 systematic reviews, 4 experimental studies (e.g. randomized controlled trial), 13 qualitative studies, 3 survey studies, 1 concept analysis, 1 methodology paper, 4 reviews, 2 position papers, 1 background paper, 1 status report, 1 commentary, 1 opinion paper, and 1 perspective.

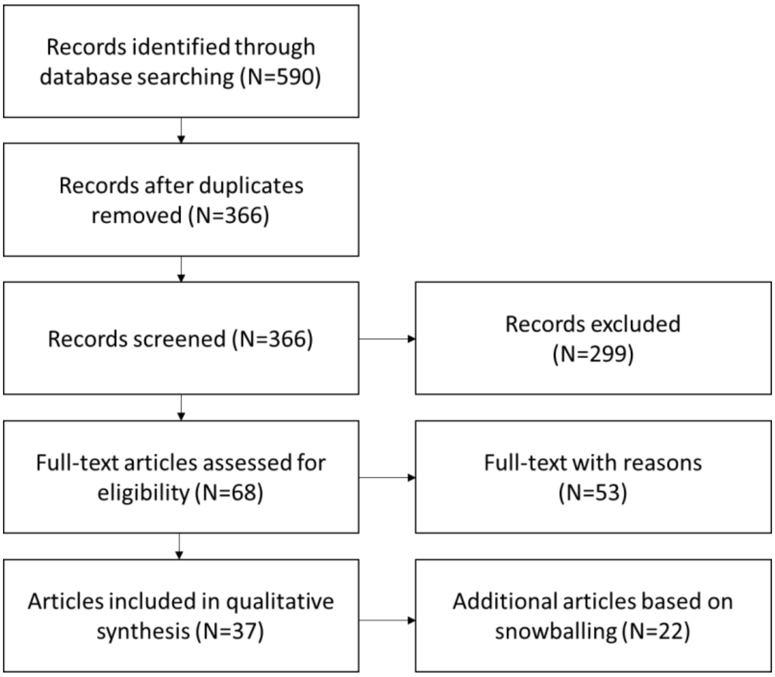

Fig. 1 Flow chart demonstrating the search string.

Table 3. Overview of the included articles.

**Papers identified based on full text screening**				
No.	Year	Authors	Title	Study design + method
1	1991	Mold, Blake, Lorne, Becker [13]	Goal-oriented medical care.	Position paper
2	2011	De Maeseneer, Boeckxstaens [30]	Care for non-communicable diseases (NCD's): time for a paradigm-shift.	Opinion paper
3	2012	Reuben, Tinetti [10]	Goal-oriented patient care- an alternative health outcomes paradigm.	Perspective
4	2014	Bayliss, Bonds, Boyd, Davis, Finke, Fox, Stange [31]	Understanding the context of health for persons with multiple chronic conditions: moving from what is the matter to what matters.	Forty-five experts met to critically consider four aspects of incorporating context into research on multiple chronic conditions.
5	2014	Kramer, Bauer, Dicker, Durusu-Tranriover, Ferreira, Rigby, van Hulsteijn [8]	The changing face of internal medicine: patient- centered care.	Position paper
6	2015	Bernsten, Gammon, Steinsbekk, Salamonsen, Foss, Ruland, Fonnebo [32]	How do we deal with multiple goals for care within an individual patient trajectory? A document content analysis of health service research papers on goals for care.	Document content analysis of seventy health service research papers on the topic of 'goals of care'.
7	2016	Blom, Elzen, Houwelingen, Heijmans, Stijnen, Van Den Hout, Gussekloo [33]	Effectiveness and cost-effectiveness of a proactive, goal-oriented, integrated care model in general practice for older people. A cluster randomized controlled trial: integrated systematic care for older people-the ISCOPE study.	Cluster randomized controlled trial –intervention group: general practitioners made an integrated care plan using functional geriatric approach; control group: care as usual; 59 general practices were included (30 intervention, 29 control); outcome measures on quality of life, activities of daily living, satisfaction with delivered healthcare, and cost-effectiveness of the intervention 1-year follow-up.

8	2016	Boeckxstaens, Willems, Lanssens, Decuypere, Brusselle, Kühlein, Sutter [34]	A qualitative interpretation of challenges associated with helping patients with multiple chronic diseases identify their goals.	Qualitative research – qualitative interviews with nineteen patients diagnosed with chronic, obstructive pulmonary disease and comorbidities to explore goal-setting in patients with multimorbidity.
9	2016	Mangin, Stephen, Bismah, Risdon [35]	Making patient values visible in healthcare: a systematic review of tools to assess patient treatment priorities and preferences in the context of multimorbidity.	Systematic review – data sources: Medline, Embase, Cochrane databases; citations were included if they reported a tool to use a record patient priorities or preferences for treatment, and quantitative or qualitative results following administration of the tool.
10	2016	Schimdt, Babac, Pauer, Damm, von der Schulenberg [36]	Measuring patients priorities using the Analytic hierarchy process in comparison with best-worst scaling and rating cards: methodological aspects and ranking tasks.	Analysis of the results of non-standardized Analytic Hierarchy Process (AHP)for different consistency ration threshold, aggregation methods, and sensitivity analysis; comparison of rakings criteria of AHP with best-worst-scaling and ranking cards results by Kendall's tau b.
11	2016	Tinetti, Esterson, Ferris, Posner, Blaum [1]	Patient priority-directed decision making and care for older adults with multiple chronic conditions.	Review
12	2018	Bernsten, Hoyem, Lettrem, Rul, Rumpsfeld, Gammon [6]	A person-centered integrated care quality framework, based on qualitative study of patient's evaluation of care in light of chronic care ideals.	Qualitative evaluative review of the individual patient pathways experiences of nineteen strategically chosen persons with multimorbidity.
13	2019	Feder, Kiwak, Costello, Dindo, Hern, Bigos, Naik [3]	Perspective of patients in identifying their values-based health priorities.	Qualitative study using in-depth semi structured telephone and in-person interviews; open-ended questions about patient perceptions of the patient health priorities identification process, perceived benefits of the process, enables and barriers to PHPI, and recommendation for process enhancement.
14	2019	Franklin, Lewis, Willis, Roger,	Controlled, constrained or flexible? How self-management goals are shaped by patient-provider interactions.	Conversation analysis; observations of consultations for chronic care management between patients and their health professionals.

		Venville, Smith [37]		
15	2019	Tinetti, Dindo, Smith, Blaum, Costello, Ouellet, Naik [38]	Challenges and strategies in patient's health priorities-aligned decision-making for older adults with multiple chronic conditions.	Participant observation qualitative study – clinicians followed a training and had experiences in providing patient priorities care (PPC), clinicians and PPC implementation team participated in 21 case-based, group discussions. Using emergent learning, participants discussed challenges, posed solutions, and worked together to determine how to align care options with the health priorities of 35 patients participating in the patient priorities care pilot.
Papers identified through snowballing				
No.	Year	Authors	Title	Study design
16	2006	Hurn, Kneebone, Cropley [39]	Goal setting as an outcome measure: a systematic review	Systematic review – data sources included a computer-aid literature search of studies examining the reliability, validity, and sensitivity of goal-setting/ goal-attainment scaling, with snowballing.
17	2009	Bodenheimer, Handley [40]	Goal-setting for behavior change in primary care: an exploration and status report.	Exploration and Status report – literature search on goal-setting interventions for promoting behavior change; resulting in eight articles.
18	2011	Junius-Walker, Stolberg, Steinke, Theile, Hummers-Pradier, Dierks [41]	Health and treatment priorities of older patients and their general practitioners: a cross-sectional study.	Cross-sectional study – 123 older patients and 11 general practitioners evaluated the importance and severity of patients' individual health problems. Patients received a geriatric assessment, then GPS rated the importance and components of severity of each problem; assessing proportion of important problems and the chance corrected agreement; multilevel logistic regression models were used to relate the importance of a problem with its severity components.

19	2012	Rijken, Bekkema, Boeckxstaens, Schellevis, De Maeseneer, Groenewegen [2]	Chronic disease management programs: an adequate response to patients' needs?	Survey among country-experts resulting in information about existing disease management programs; in addition scientific literature.
20	2014	Lenzen, Daniëls, van Bokhoven, der Weijden, Beurskens [42]	Setting goals in chronic care: shared decision making as self-management support by the family physician.	Background paper to contribute to the understanding of goal-setting within self-management and to identify elements that need further development for practical use.
21	2016	Steel Gray, Wodchis, Upshur, Cott, McKinstry, Mercer, Palen, Ramsay, Thavorn [43]	Supporting goal-oriented primary health care for seniors with complex care needs using mobile technology: evaluation and implementation of the health system performance research network, Bridgepoint electronic patient reported outcome tool.	Pragmatic cluster randomized controlled trial – intervention groups using ePRO tool compared with control groups on measure of quality of life, patient experience, and cost-effectiveness; evaluating of tool.
22	2017	Kangovi, Mitra, Smith, Kulkarni, Turr, Huo, Glanz, Grande, Long [44]	Decision-making and goal-setting in chronic disease management: baseline findings of a randomized controlled trial.	Randomized controlled trial – patients used low-literacy aid to prioritize one of their chronic conditions and then set a goal for that condition with their primary care provider; patients created patient-driven action plans for reaching these goals.
23	2017	Mold [45]	Goal-directed health care: redefining health and health care in the era of value-based care.	Review
24	2017	Schellinger, Anderson, Frazer, Cain [46]	Patient self-defined goals: essentials of person-centered care for serious illness.	Descriptive qualitative analysis – initial inquiry to describe self-defined goals patients living with advanced heart failure, cancer, and dementia; goals were entered in electronic health record flow sheet using patients' quotes; analysis of 160 flow sheets with a deductive approach.
25	2017	Vermunt, Harmsen, Elwyn, Westert, Burgers, Rikkert, Faber [47]	A three-goal model for patients with multimorbidity: a qualitative approach.	Qualitative study – qualitative interviews with general practitioners and clinical geriatricians and analyzed following a thematic approach.

26	2017	Vermunt, Harmsen, Westert, Rikkert, Faber [17]	Collaborative goal setting with elderly patients with chronic disease or multimorbidity: a systematic review.	Systematic review based on EPOC, PRISMA and MOOSE guidelines; Pubmed, PsychInfo, CINAHL, Web of Science, Embase, Cochrane Central Register of Controlled Trials were searched systematically; eligibility criteria: 1) Randomized (cluster) controlled trials, non-randomized controlled trials, controlled before-after studies, interrupted time series or repeated measures study design; 2) Single intervention directed specifically at collaborative goal setting or health priority setting or a multifactorial intervention including these elements; 3) Study population of patients with multimorbidity or at least one chronic disease (mean age ± standard deviation (SD) incl. age 65). 4) Studies reporting on outcome measures reducible to outcomes for collaborative goal setting or health priority setting.
27	2018	Kessler, Walker, Sauvé-Schenk, Egan [29]	Goal setting dynamics that facilitate or impede a client-centered approach.	Conversation analysis on goal-setting conversations; purposively selected from a pilot randomized controlled trial of OPC-stroke
28	2018	Naik, Dindo, Van Liew, Hundt, Vo, Hernandez-Bigos, Esterson, Geda, Rosen, Blaum, Tinetti [4]	Development of a clinically feasible process for identifying individual health priorities.	Prospective development and feasibility study – development team of patients, caregivers, clinicians using a user-centered design to develop and refine value-based patient priorities care process and medical record template; descriptive statistics and qualitative analysis of barriers and enablers.
29	2019	De Groot, Schönrock-Adema, Zwart, Damoiseaux, Jaarsma, Mol, Bombeke [48]	Learning from patients about patient-centeredness: a realist review: BEME guide No.60	Realist review – realist review approach; literature search in scoping phase, deductive and inductive coding to extent rough program theory.

30	2019	Kuluski, Guilcher [49]	Towards a person-centred learning health system: understanding value from the perspectives of patients and caregivers.	Commentary; call to action to combine the tenets from person-centered care, value-based healthcare, and learning health systems.
31	2019	Kuluski, Peckham, Gill, Gagnon, Wong-Cornall, McKillop, Parsons, Sheridan [9]	What is important to older people with multimorbidity and their caregivers? Identifying attributes of person centered care from the user perspective.	Qualitative descriptive study; 1-1 interviews semi-structured interviews with 172 patients and caregivers from 9 community based primary healthcare.
32	2019	Reuben, Jennings [12]	Putting goal-oriented patient care into practice.	Review
33	2019	Salter, Shiner, Lenaghan, Murdoch, Ford, Winterburn, Steel [28]	Setting goals with patients living with multimorbidity: qualitative analysis of general practice consultations.	Qualitative analysis of general practice consultations – analysis of video recorded doctor-patient interactions; focus groups to identify core challenges of goal-setting.
34	2019	Tinetti, Naik, Dindo, Costello, Esterson, Geda, Rosen, Hernandez-Bigos, Smith, Ouellet, Kang, Lee, Blaum [50]	Association of patient priorities-aligned decision-making with patient outcomes and ambulatory health care burden among older adults with multiple chronic conditions.	Nonrandomized clinical trial with propensity adjustment conducted at one patient priorities care (PPC)and one usual care; participants included 163 adults aged 65 years or older who had three or more chronic conditions care for by ten primary care practitioners (PCP) trained in PPC and 203 similar patients who received usual care from 7 PCPs not trained in PPC.
35	2020	Eckhoff, Weiss [51]	Goal-setting: a concept analysis	Concept analysis – method of Walker and Avant, articles and book chapters were reviewed from Cumulative Index to Nursing and Allied Health Literature, Education Resources Information Center, Psych Index.
36	2020	Purkaple, Nagyaldi, Todd, Mold [52]	Physician's respone to patient's quality-of-life goals.	Randomized controlled trial – patients were given a previsit questionnaire that included quality of life questions; physicians in the control were given no further prompting; intervention physicians were prompted to ask quality of life questions; a two-

				pronged design was used: prepost group where three physicians participated in 5 control and 5 intervention encounters (n = 30) and a randomized group in which 11 physicians and their patients were randomly assigned to control or intervention groups (n = 30). Video recordings of the encounters were reviewed to determine if QOL goals were mentioned and if they were utilized in decision making.
37	2020	Sathanpally, Sidhu, Fahami, Gillies, Kadam, Davies, Khunti, Seidu [53]	Priorities of patients with multimorbidity and of clinicians regarding treatment and health outcomes: a systematic mixed studies review.	Systematic review – MEDLINE, EMBASE, CINAHL, and Cochrane databases were searched; included studies reported health outcome and treatment priorities of adults with multimorbidity, defined as suffering from two or more chronic conditions, or of clinicians in the context of multimorbidity or both; no restriction by study design, and studies using quantitative and/ or qualitative methodologies were included.

Step 4: attributes

The systematic analysis of the 37 selected papers could identify many different attributes of goal-oriented care (S1 Table 1). Synthesizing these attributes, goal-oriented care could be described as a multifaceted dynamic and iterative process of care (first main attribute) underpinned by patients' values (second main attribute). For the process of goal-oriented care five~~5~~ sub attributes and ~~7~~seven descriptive items could be identified (Table 4~~3~~). These attributes interact and cannot be interpreted separately.

Table 4~~3~~. Overview of attributes.

<table>
<tr><td rowspan="8">~~1.~~ Goal-oriented care is a multifaceted, dynamic and iterative process.
[1, 3, 4, 6, 12, 13, 17, 28, 29, 40, 42, 43, 46, 54, 55]</td><td colspan="2">1.1 Goal-elicitation builds a patient-provider relationship. [1, 28, 29, 45, 56]</td></tr>
<tr><td rowspan="4">1.2 Goal-oriented care entails goal-setting.</td><td>1.2.1Patient-provider interaction guides goal-setting.[2, 4, 12, 13, 17, 28, 29, 35, 40, 42-45, 47, 49]</td></tr>
<tr><td>1.2.2 Patients' needs and preferences are the foundation of SMART formulated goals. [1-4, 6, 10, 13, 28, 29, 31, 35, 37, 41, 44, 46, 49, 50, 56, 57]</td></tr>
<tr><td>1.2.3 Care plan is based on patients' needs and preferences. [1, 3, 4, 6, 10, 12, 13, 17, 31, 33, 35, 38]</td></tr>
<tr><td>1.2.4 Care is delivered according to the care plan.[1, 6]</td></tr>
<tr><td rowspan="3">1.3 Goal-evaluation is a reflexive process.</td><td>1.3.1 Feedback should be given to the goals. [38, 54]</td></tr>
<tr><td>1.3.2 Evaluation entails questioning how goals are being met. [12]</td></tr>
<tr><td>1.3.3 Goals must be measurable. [13], 33)</td></tr>
<tr><td rowspan="2">2. Goal-oriented care embraces patients' values.</td><td colspan="2">2.1 Goal-oriented care must be placed in patients' context. [3, 12, 31, 35, 42]</td></tr>
<tr><td colspan="2">2.2 Goal-oriented care must be tailored to patients' needs and preferences. [1, 6, 28, 29, 38]</td></tr>
</table>

Goal-oriented care is a multifaceted, dynamic and iterative process

The majority of the authors presented goal-oriented care as a stepwise approach [1, 3, 4, 6, 12, 13, 17, 28, 29, 40, 42, 43, 46, 54, 55]. Even though every paper defined their own approach, overall three stages could be identified: (a) goal-elicitation, (b) the actual stage of goal-setting, and (c) a reflexive goal-evaluation stage. These three stages will be further discussed.

Bernsten et al. [6] emphasized the dynamic and iterative characteristics of the goal-oriented process of care [6]. They described that goal-oriented care entails going back and forth between the three stages [6]. From this perspective, goals are not described as an endpoint, but they can be adjusted, discarded, modified or new goals might be set [12, 38]. This will be further discussed in the stage of goal-evaluation.

Overall, in the goal-oriented process of care, the patient is described as an active partner [1]. Therefore, a good communication in a continuous patient-provider relationship is described to be of utmost importance [46]. In addition, goal-oriented care should be considered as care over time rather than a one-time intervention [58]. In terms of outcomes, it is not entirely clear whether goal-oriented care should focus on (a) maintaining the status quo or (b) improving the patients' situation [12]. Although there is consensus that the care process is oriented to the current needed care rather than care needed in the future [1].

Goal-elicitation builds a patient-provider relationship

Formatted: Font: 14 pt

As described earlier, the overall analysis could identify goal-elicitation as the first stage in the process of goal-oriented care. In this first stage, providers are presumed to offer time and space to patients to tell their stories in order to work towards the patients' agenda [29]. Therefore, patients have to be ready and should be actively encouraged to tell their story. Tinetti and colleagues described this as 'the patient's state of readiness' [1]. This first stage is considered to be essential to work towards a balanced patient-provider conversation and relation [52, 56]. Salter et al. described this stage as a shared process between patients and providers that reinforces and further builds their relationship

[28]. This specific part of the process of goal-oriented care is also described as a mean to achieve a greater level of shared understanding and mutual commitment between the patient and the provider [45]. Specific attention to the stage of goal-elicitation is described to create a supportive context for effective goal-setting in the next stage [28].

Goal-oriented care entails goal-setting

Formatted: Font: 14 pt

Besides the goal-elicitation stage, the literature identifies a goal-setting stage. Franklin and colleagues analyzed patient-provider conversations during goal-setting and concluded that the goal-setting stage serves as a mechanism to embrace patients' needs within the social context he lives in [37]. When this process is done properly, goal-setting should support the patients to continue doing what matters most to them which would help ~~. This would help~~ them to cope with their conditions [37]. Within this process of goal-setting different sub attributes, that are considered necessary for proper goal-setting, could be identified. ~~that are considered necessary for proper goal-setting~~

Patient-provider interaction guides goal-setting

Formatted: Font: 14 pt

The patient-provider interaction is characterized by a patient-centered approach [28] in which goals are set in collaboration [47]. Hereby, patients and providers agree on health-related goals [2, 12, 13, 40, 43, 47, 55, 59] and find common ground [58]. Tinetti et al. described the importance of considering patients as active partners in the goal-setting process [38]. Rijken et al. mentioned that patients' goals have to be discussed in a dynamic conversation continuously taking the patients' needs, preferences, and abilities into account [2].

To facilitate a collaborative approach it is suggested that providers emphasize the patients' narratives reflecting their lived experience [45]. ~~Next to~~Besides a collaborative approach, negotiation is important and considered inevitable [4, 6, 28, 42, 54]. Lenzen et al. defined this as goal-negotiation, which involves discussion of any kind of problems, exploration of the patients' values, needs and capabilities, and deliberation on patients' goals [42]. In goal-negotiation, formulating and agreeing on a specific goal are important components [28].

~~Since~~ Because the goal-setting process needs to be driven by patients' needs and preferences, there seems to be a general understanding to shift the focus from the provider to the patient [29]. Different authors reported various strategies to facilitate this shift. Mold stated that the shift implies that prioritization of the individual health-related goals and the amount of effort in achieving them should be made by the individual [13]. Naik et al. stated that patients are indeed encouraged to share their priorities, but adds that providers are encouraged to align their care with the patients' health priorities [4]. More recent publications talking about goal-setting describe a circular and shared process aimed at improving the balance and power differentials in the patient-provider relationship [4, 44]. This balance can be improved by putting themselves in someone's shoes to understand the other's constraints [49].

Patients' needs and preferences are the foundation to set goals

Formatted: Font: 14 pt

One of the important challenges in our understanding of the concept of goal-oriented care is the lack of clear understanding on patient goals. Nearly all authors described that goals should be grounded on the patients' needs and preferences [1-4, 6, 28, 29, 37, 38, 44, 46, 52, 54, 58, 60, 61]. It is described that goals should be based on the context, resources and capabilities of patients [52], that they should be approved by patients [6], and that they should foremost represent what the patients want and not necessarily what the providers want [12, 46]. Other authors recommended that goals should be a combination of both the patients' goals and the providers' goals which in turn is related to goal-negotiation ~~the combination of patients' and providers' goals which could be related to the aspect of goal-negotiation~~ [29, 49]. In conclusion, no overall understanding on the goals could be formulated.

Besides this lack in understanding, there also seems to be ambiguity about the categorization of goals.

~~There also seems to be some confusion in the categorization of goals.~~ Some authors emphasized that goals should contain core values of patients (e.g. the broader aspects that matter most to the patient) [1, 4] . These goals are named as 'overarching goals' [6, 12, 29, 46] leading to a broad description of the goal (e.g. I want to live in my own home as long as possible [1]) [6]. Others argued that these

overarching goals might not be easy to work with and describe that these goals should be broken down into sub goals (e.g. I want to walk 2 blocks without shortness of breath [1]) [6]. Goals differ for each individual and will change over time [13]. Aside from overarching goals and sub goals many of the authors mention the importance of setting SMART goals [1, 6, 28, 29, 40, 51, 54, 55, 58]. A SMART goal is created when patients and providers collaborate to untangle the goal itself, the importance of that goal is emphasized to the patient, the perceived achievability of the goal is evaluated, as well as the timing of the goal, and any supports and resources available [40]. On the meta-perspective, overarching goals are too broad to make SMART (think about the grandmother aiming to get her grandchildren from school as long as possible). Therefore they should be divided in the sub-goals (~~in sub goals~~ such as I need to be able to walk without being tired after 10 yards) that are specific enough to be measured.

In one of his first publications Mold brings in a specific discourse around the ~~type~~ categorization of goals, namely that goal-oriented care should assist patients in achieving their maximum individual health potential [13], hereby making the link with health. One should however notice that health should be described from the patients' perspective; as the ability to live his life, and not as the absence of disease [1, 13]. Patients' goals are oriented towards health outcome goals. Patients hope to achieve these individual health outcomes through their health care (e.g. function, social activities, and symptom relief)[1]. Health outcome goals describe activities that promote change in physical and cognitive well-being or health [41]. Naik et al. specifically relate patient goals to the care they are willing to receive and able to perform [4].

Care plan is based on patients' needs and preferences

Formatted: Font: 14 pt

Many authors relate goal-oriented care to the construction of a care plan based on the patients' needs and preferences and specifically mention that these care plans should reflect the patients' personal goals that have been identified in the previous stage [1-3, 6, 12, 31, 33]. There is a consensus that the care plan should reflect the question: 'What matters to you?' [12, 38, 49, 54, 60]. Strategies to achieve

the patients' needs and preferences should be implemented in the care plan [13]. Furthermore, Bernsten and colleagues stated that the care plan might also include an interprofessional review of the goals [6]. Therefore, it is necessary to involve all providers and preferably patients' informal care-givers and family in the whole process [3, 6, 17]. In case that providers are confronted with patients' goals that are out of their own scope, they could benefit from an interprofessional review as they are enabled to discuss with and hand over to other providers with the required expertise. This could improve the coordination of the care plans between the different providers and facilitate integrated care delivery [1, 4, 35]. To guide this interprofessional review, no specification was given about which profile would be the best fit for having the lead. Vermunt et al. (2017) illustrated this as they found variation in who (e.g. GP, nurse, practice nurse, psychological wellbeing practitioner) should contribute to goal-setting [17].

An interprofessional review of the goals might benefit the coordination of the care plans between the different providers and facilitate integrated care delivery [1, 4, 35].

Care delivery according to the care plan

Formatted: Font: 14 pt

Patients and providers should implement the care plan and translate it into care delivery. Although, little is known about how care should be delivered, it is evident that it must be in accordance with the care plan that is set up in the previous stage [6]. For this stage Tinetti et al. specifically mentioned to start the stage of care delivery by prioritizing on simple interventions in order to achieve one or more small goals to keep patients motivated [1]. This simple interventions could focus on the sub-goals described in previous paragraphs to eventually work towards the overarching goals.

Goal-evaluation is a reflective process

Formatted: Font: 14 pt

The overall synthesis/analysis of the literature could identify goal-evaluation as the third and final stage in the process of goal-oriented care. For this stage authors described a dynamic and iterative process that allows reflection and feedback next to assessing whether and how goals have been met

[38, 54]. In this process goals can be redefined and adjusted. Possible reasons to adjust goals might be that goals have been too difficult to achieve or were no longer desired or relevant to the patients' situation [12]. Although many authors acknowledge the possibility and importance of goal adjustment, there is also discussion that goal-oriented processes of care requires that goals can be measured [13]. Steele Gray and colleagues described the importance of qualifying and quantifying the process proceeded to achieve the goals [43]. In contrast, Salter and colleagues described that making the goals measurable could overcomplicate and distance the patient from their own goal and might therefore not be beneficial to the process of goal-oriented care [28].

Goal-oriented care embraces patients' values

In the previous attributes, goal-oriented care is described as a dynamic and iterative process in which two underpinning values are identified [4]. First~~ly~~, goal-oriented care must be placed in the patient's context and second~~ly~~, goal-oriented care must be tailored to the patient's needs and preferences.

Goal-oriented care must be placed in patients' context

Formatted: Font: 14 pt

The whole goal-oriented process of care starting from goal-elicitation to goal-evaluation needs to be placed in the patient's context. According to different authors this means that the process must be tailored to the patient's situation [3, 12, 42, 60]. This does not only refer to the personal context, but also to the social and the cultural context. Therefore, this process is influenced by different contextual factors that should must be taken into account when developing the care plan [35, 42].

Goal-oriented care must be tailored to patients' needs and preferences

Formatted: Font: 14 pt

When reviewing the attributes, it is clear that patients' needs and preferences form the common thread. The question 'What is the matter with the patient' must be retranslated to 'What matters to the patient?' [1, 6, 28, 38]. This question enables patients to tell their story and open up in which they are considered to reflect on their achievements and personal agenda [29]. As a result, patients will have the feeling to be approached as a person instead of through their condition [6].

CASES

The method of Walker and Avant prescribes that several cases should be described to illustrate the attributes defined in step 4 [25]. The first case of Joseph encompasses all the attributes identified in the literature and is therefore identified as a model case. It is a fictive example of delivering care according to the goal-oriented process of care with focus on the underpinning attributes. The second case of Ben is identified as an additional case as ~~sin~~it lacks one or more of the attributes. E.g. in the case of Ben the stage of goal-evaluation is missing. This stage is needed to make adjustment and reflections according to the process of achieving the personal goals. Finally, the third case of Mary is an example of the opposite of goal-oriented care. This is described as a contrary case. In this case, the health care provider does not take the needs and preferences of Mary into account. The provider only

thinks about convincing Mary of a healthy lifestyle which for her is not the main reason to visit her health care provider. Her main focus is on being able to go on a city trip to Madrid.

Joseph, 68- year old suffers from diabetes, hypertension and ~~cardiovascular disease~~chronic obstructive pulmonary disease. Throughout his entire working life, he was a secondary school teacher. He has been retired for three years now. Despite the fact that he is limited by his health condition, he loves spending time with gardening and playing with his grandchildren.<br>A few years ago he was a passionate cyclist, but his racing bike has been stored for a long time now. His friends encourage him to cycle with them on a weekly base. His wife supports this initiative and argues that this will be beneficial for his social contact.<br>Every month Joseph visits his family doctor for a check-up. For each consultation, he prepares a list of things he wants to discuss. He has the chance to share his story in an open communication in which trust and mutual respect are key components.<br>In his monthly check-up with his family doctor he suggests his wishes to cycle again with his friends. His doctor doubts whether this will be possible and after discussion and negotiation, they plan that he would join his friends in their weekly cycling trip but only for the first two hours. The group will be asked to adapt their pace and Joseph will make sure that he does not need to return back home on his own. The doctor makes adjustments to the medication scheme according to the increased efforts Joseph will make. He will also contact the cardiologist to inform him about the changes to the medication schema. The family doctor and the cardiologist will collaborate in order to succeed in Joseph's goal.<br>The family doctor and Joseph agree to discuss and evaluate the course after three months. It is possible to increase or decrease the intensity depending on Joseph's health state and his own preferences.

Box 1 Model case of Joseph

Ben, a 30-year old man, was renovating a house that he bought with his girlfriend when he was diagnosed with MS. They made plans to marry next year and to make a world trip as honeymoon. These plans have been put aside due to the recent diagnosis. Although he was feeling down and did not have the energy to do anything he ended up with an excellent physician. Initiated by the interaction and the conversation with his physician he was enabled to set goals again and to look

Box 2 Additional case of Ben

Mary is a 40-year old mother of two young children and dealing with obesity since her childhood. Due to her weight, she has a lot of joints pain and is short of breath which limits her exercising capacity. Her children are already looking forward to playing outside with their mother during the summer holidays. Unfortunately, she is not able to play soccer or jump on the trampoline because of the pain. The pain becomes too much for her and after long hesitation she discusses this with her physician. The only thing she wants is to play and interact with her children as painless as possible and therefore asks her physician to prescribe some medication. Her physician does not support medication, but instructs her to first strive for a healthy weight as a solution to relieve the pain. This is not aligned with the wishes of Mary who only wanted a short-term solution to be able to play with her children. ~~In the upcoming summer, she wants to make a city trip with the entire family to Madrid. Therefore she is seeing her physician to discuss the options to travel as painless and comfortable as possible. Her physician does not allow the travel plans and instructs her to first strive for a healthy weight and then plan trip when she has lost weight. This is not aligned with the wishes of Mary who only want's a short-term solution to cope with her condition during the city trip.~~ In the end, she leaves the consultation room with a referral to a dietitian and sport coach.

Box 3 Contrary case of Mary

Antecedents

Antecedents are events or incidents that occur prior to the investigated concept. In this concept analysis, provider preparedness and patient preparedness are required to provide goal-oriented care.

In terms of provider preparedness many authors discussed the importance of training [6, 7, 29, 33, 37, 47, 55]. Notwithstanding that several authors [1, 4, 17, 28, 33, 38, 44] mentioned the importance of trained health care providers, there was a difference in the training they received (supplementary file ~~2~~3). Differences can be found in the target population reached with the training, both in monodisciplinary and interprofessional training (e.g. general practitioners [28], practice nurses [33], duration of the training (e.g. three hour [28], number of sessions [33]) and training method (e.g. role-play [38])~~-~~. Thereby, the content of the training was tailored to the skills needed to carry out the intervention correctly and differ therefore in each training (S3 Table 3).

A second aspect that is discussed concerning provider preparedness focused on the personal skills of providers [1, 6, 17, 28]. These include communication and balancing skills in which an open communication with the patient is necessary and in which an equal balance between the patient and provider is a premise [1, 6, 17, 28]. Other defined skills were the provider's ability to listen, understand and bearing witness to the patient's story [28] and their willingness to change and learn new skills to provide care according to the goal-oriented process of care [1].

~~Next~~ Besides~~to~~ provider preparedness some authors [1, 12, 47] specifically talked about the need of patient preparedness. Patients needed to be prepared to share their needs and preferences when entering a care relationship [1]. Some authors translate the importance of patient preparedness into patient education [1], others talked about patient guidance (11) or supporting patients in developing the skills to set personal goals [42].

Consequences

Consequences are those events or incidents that occur as a result of a concept. For the concept of goal-oriented care, the consequences defined throughout the papers could be categorized in: (a) patient-related consequences [1, 3, 4, 29, 35, 54], (b) provider-related consequences [1, 28, 35, 54], (c) care-related consequences [1, 28, 35] and (d) general consequences [4, 6, 35].

Patient-related consequences are the results for patients themselves after they received care following a goal-oriented process. A goal-directed approach could be expected to increase patient satisfaction, since the values, preferences, knowledge and opinions that each patient brought to the provider-patient relationship was more valued [45]. Also, emphasis was put on the changed way of communicating in which patients felt more freely and able to speak [3]. This led to the overall feeling of being heard, understood, respected and engaged in their care [35]. Furthermore, a goal-oriented process of care could lead to a better understanding and more in-depth knowledge of patients regarding their health, activation of patients to be more involved in their care and an increase in their overall commitment. This resulted in the increase of adherence [3]. Also Mold argued that it could contribute to a better adherence [13]. In general, the gained in-depth knowledge of patients concerning their health and a better understanding of their tasks could help to improve their quality of life [3]. This was enhanced by the maximization of function and the independence~~y~~ patients gained [13].

For providers, goal-oriented care assisted healthcare them in their decision-making [35] and gave them the opportunity to get to know their patients better. It enhanced patient-provider collaboration [13] and contributed therefore to more job satisfaction [28].

Care-related consequences were mainly focused on reducing costs, overtreatment and fragmentation [1, 28, 35], since care oriented to patients' priorities would reduce tests and treatments [50]. Bernsten

et al. stated also that goal-oriented care could lead to an improvement of quality of care and quality of life [6].

Although, many positive outcomes have been presented, Reuben et al. mentioned a possible downside of goal-oriented care [10]. They described that some decisions to strive for personal goals may worsen the providers' performance on aggregated health measures. For example, when a diabetic patient chooses to not follow his diet and keep on smoking, because it would be a too big lifestyle change, his HbA1c-level would not be aligned with the guidelines. Although, it could be a positive outcome from the patient perspective, it would influence the quality of care provided and the population health in a negative way.

Empirical referents

Empirical referents provide an overview of the identified assessments tools related to the attributes aiming to make the concept measurable.

None of the papers mentioned an empirical referent to measure the entire concept of goal-oriented care. Therefore, tools have been searched for each individual sub-attribute. Examples are listed in tTable 45 which gives an overview of possible tools and presents an example item presented in that tool. Listing the existing individual empirical referents might initiate the development of an overall empirical referent.

Formatted: English (United States)

Table 45. Overview empirical referents.

Attribute	Purpose of the tool	Example of item in the assessment tool
Goal-elicitation		
Davis Observation Code (DOC) [62]	20-item direct observation scale for physician-patient interactions	Discussing family, medical, or social history and/ or current family functioning.
Goal-setting		
Patient goal priority questionnaire [63]	Patient-specific measure for identification of behavioral goals and evaluation of clinically significant changes	Which activities are most important for you to manage?
Self-identified goals assessment [64]	1) Helps patients to identify personally meaningful occupational goals to be addressed in therapy<br>2) evaluate changes levels of patient-defined success in desired occupations	Think about all of the things you want to be able to do. It might help to think about the things you did at<br>home before you went to the hospital, and things that are hard to do now. What types of things would you like<br>to work on or improve on in therapy before you go back home?
Canadian Occupational Performance Measure (COPM) COPM [65]	Measure of a client's self-perception of occupational performance in the areas of self-care, productivity, and leisure	Semi-structured interview – discussing daily functioning and personal life.
Health outcome prioritization tool [66]	Tool for decision-making among older persons with multiple chronic conditions	I would like to know how important 'keeping you alive', 'maintaining independence', 'reducing or eliminating pain' and 'reducing or eliminating symptoms of dizziness, fatigue, shortness of breath' is to you.
Electronic Patient Reported Outcome Tool (EePRO-tool) [67]	Tool can help patients and providers to collaboratively develop healthcare goals	Goal-setting for five different areas identified as most important.
Goal-evaluation		
Goal-attainment scale [68]	Tool to measure in which extent patients' goals have been met	Determining goal-attainment using 5-point scale.
Patient Assessment of Care for Chronic Conditions (PACIC) [69]	Tool to measure quality of chronic disease care	Asked to talk about my goals in caring for my condition.

Formatted: Font:

Formatted: Position: Horizontal: 0", Relative to: Page, Vertical: 0.33", Relative to: Paragraph

Formatted: English (United States)

Formatted: English (United States)

Formatted: English (United States)

Formatted: English (United States)

Formatted: English (United States)

Formatted: English (United States)

Formatted: English (United States)

Goal-setting evaluation tool [70]	Tool to rate the quality of goals and action plans	Does the plan identify specific actions or activities that could help to reach the goal?
Person's context and patient's needs and preferences		
Person-centered primary care measure (PCPCM) [71]	11-item patient-reported measure to assess primary care aspects	My doctor or practice knows me as a person/ Over time, the practice helps me to meet my goals.
Patient centered observation form (PCOF) [72]	Tool to help healthcare providers communicate effectively with patients	Collaborative upfront agenda setting.

Formatted: English (United States)

CONCLUSION OF THE CONCEPT ANALYSIS

Fig. 2 represents the overall synthesis of this concept analysis of goal-oriented care. Goal-oriented care could be described as a health care approach encompassing a multifaceted, dynamic and iterative process underpinned by the patient's context and values. The process is characterized by three stages: goal-elicitation, goal-setting and goal-evaluation in which patients' needs and preferences form the common thread. In order to be able to deliver care according to the principles of the goal-oriented care process, both providers and patients need to be prepared. In terms of the consequences of goal-oriented care literature points to the potential of goal-oriented care to improve patients' experiences and provider well-being, the potential to reduce costs and improve the overall health of the population. Furthermore, a model, a contrary and an additional case illustrated an example of goal-oriented care in practice. The empirical referents showed that it is currently not possible to measure goal-oriented care in its entirety and presented an overview of possible referents for each sub attribute. Although the literature allowed us to gain more insight into the concept of goal-oriented care, different aspects need to be further discussed.

Fig. 2 Schematic representation of the antecedents, attributes and consequences.

Discussion and conclusion

This concept analysis aimed to tackle the lack of a common understanding of goal-oriented care by identifying the attributes, antecedents and consequences using the method of Walker and Avant [25]. The overall analysis showed that a goal-oriented care generally entails three stages. Despite these three stages the process of goal-oriented care cannot be implemented as a linear protocol or checklist. Two underpinning attributes, the patient's context and the patient's needs and preferences form the common thread throughout this goal-oriented process of care. These underpinning attributes represent the philosophy of care. Goal-oriented care is a continuous interaction where you go back and forth to gain a person-centered approach (Fig. 2).

In the stage of goal-elicitation, greater consideration should be given to the patients' peripheral narrative reflecting their lived experiences [37]. Several authors have investigated components of goal-elicitation. Murdoch and colleagues performed a conversation analysis of patients-providers interaction during their encounters and found that eliciting the patients' understanding is an important component [73]. Ospina et al. investigated the extent to which patients' concerns are elicited across different clinical settings [74]. They concluded that providers seldom elicit the patients' agenda. This reduces the chance that providers will orient their consultation towards the specific aspects that matter to the patient [74]. One of the prerequisites to succeed in goal-elicitation is the mutual understanding about the expectations of the consultations between patients and providers and a qualitative relationship between patients and providers [73]. The literature also mentions that patients need to have a set of skills to make appropriate health decisions and reflect on their health care choices [75]. They have to be capable to open up and tell their story [76]. It is important that patients understand the meaning of information communicated by the provider, must appreciate the consequences of the treatment options, and must reason about the information based on his or her own values and preferences [76].

~~Next to~~Besides the stage of goal-elicitation, the stage of goal-setting wa~~i~~s defined. One of the remaining knowledge gaps is on what kind of goals patients set. In goal-oriented care it seems important to set goals based on the patients' needs and preferences (e.g. I want to take my grandchildren to school), while in other chronic disease management programs emphasis is mainly still on health-related goals (e.g. I want the patient to walk without pain) [4]. Various work in different settings identified that patients do not necessarily have clearly defined goals for themselves [73]. Although, several authors performed research on the categorization of patients' goals. Vermunt et al. performed for example a qualitative study to develop conceptual descriptions of goal-oriented care [47]. They presented a three-level goal hierarchy containing disease- or symptom specific goals, functional goals, and fundamental goals which provides more insight in the type of goals. A second example is the distinction made by Schellinger et al. between medical, nonmedical, multiple, and global goals [46]. Not only is there ~~is~~ ambiguity on what goals patients set, it is also not clear how goals are being set. What is clear is that patients and providers must collaborate and negotiate on which goals are important. Nevertheless, this can still cause conflicts between the patients' goals and providers' goals [31, 66]. To overcome these conflicts, it is suggested to first set the patients' goals and then discuss about the medical goals, because conflicts are more likely when goals are placed on the same level [32]. It should however be noticed that setting the patients' goals on top does not legitimate full patients' responsibility over the care plan [32]. Another way to overcome these conflicts is to work with a facilitator as Naik et al. did in developing their *patients priorities identification process*. These facilitators supported patients in setting goals, choosing the most important goals to eventually communicate them with the provider [4]. Yet another strategy is to use tools to assess patient treatment priorities and preferences. Unfortunately, Mangin et al. found few relevant tools to set patients' goals ~~The systematic review of tools to assess patient treatment priorities and preferences by Mangin et al. found few relevant tools to set patient's goals~~ [35]. They argue for the need to develop specific strategies to make patient priorities visible in the clinical record and medical-decision making [35].

Goal-evaluation wais pointed out as the last stage. As presented in the results, several authors described that goals should be made measurable for evaluation [28, 67]. There are some pitfalls related to goal-evaluation. Salter et al. described that not all goals lend themselves to being measured [28]. It is for example challenging to evaluate the goal ‘I want to take my grandchildren from school as long as possible’. Another pitfall is that patients’ goals would be simplified to what can be measured. Working towards goal-evaluation might increase the pressure on patients and providers to work in the same way as disease-specific guidelines do [77]. Especially from the perspective of patients with multimorbidity it can be questioned whether disease-specific guidelines that are good for the disease are also good for the patient [77]. Furthermore, evidence shows that older multimorbid patients place quantitative health outcomes, such as longer survival, on a lower level of importance [77]. The focus must be on the patients’ values and make healthcare more humane [45].

As mentioned for the antecedents it is important that patients and providers are prepared to work towards a goal-oriented process of care. The collaboration and co-creation between the two partners and in an interprofessional team is an important but insufficient prerequisite to succeed in providing goal-oriented care. Currently patients are not always stimulated to think about their care. They have to be stimulated to actively engage their narrative and to share their priorities. Also providers have to develop complementaty skills in which they learn to let go their own assumptions and solutions. They have to learn to integrate patients’ narrative in their care plan and improve their communication skills to strengthen the mutual understanding between them [78]. Voigt et al. observed that GPs are often unaware of patients’ priorities in daily life, which were in contrast with their perceived importance of patient’s medical goals [78]. Training and tools could provide the guidance needed to improve the communication[1, 4, 17, 28, 33, 38, 44]. It could support providers in structuring the conversation, to set goals in collaboration with patients, and to align their care to those goals. Not only does goal-oriented care offers a specific approach for one-on-one interaction between patients and providers, it could also facilitate interprofessional collaboration. It gives providers from divers disciplines the opportunity to deliver care following the same principles and to focus on pursuing patients’ goals [40].

Therefore training should also include the interprofessional perspective to facilitate a uniform attitude towards the patients' goals and principles of goal-oriented care in the entire team. This will potentially support providers to learn from and with each others' expertise and enable discussion between them in case that, for example, patients set goals that our out of the remit of the provider. Besides patient and provider preparedness, it could seem logical that also the system has to be prepared, but the current literature does not point to that.

In terms of the consequences of goal-oriented care, a limited number of studies have been able to demonstrate outcomes of goal-oriented care. Nonetheless, these studies showed mostly positive outcomes ~~Mostly positive outcomes have been presented~~ towards the patients, providers, health system, and overall population well-being. In that respect, goal-oriented care shows the potential to meet the components of the quadruple aim. It can be questioned if all providers experience increased satisfaction and well-being in providing goal-oriented care. Providers have to learn to cope with another way of delivering care. For example, a changed medication scheme as described in Josephs' case in order to work towards patients' goals. This goes against their basic principles to strive for the best possible health status including a comprehensive medication scheme. Besides that the provider well-being can be questioned, Blom et al. also contradicted the positive results for the health care system. They did not fi~~ou~~nd a beneficial effect in health care use and costs when using a proactive, goal-oriented, integrated care model [33].

One of the reasons of the limited number of effectiveness studies of goal-oriented care is the lack of empirical referents. The concept must still undergo the transition towards an evaluable concept. Boyd et al. argue for measures for quality of care needed by older persons with multimorbidity as the current clinical guidelines have undesirable effects for this population [57]. Goal-oriented care is ~~however~~ identified by Etz and colleagues as one of the main constructs when developing a new comprehensive measure of high-value aspects of primary care, however they did not mention how it has to be done [79]. Further~~Also~~ Young et al. described outcome goals as a main construct when differentiating processes and outcomes for primary care and divided it further in goal-clarity for multimorbidity, goal-

clarity for unique patient priorities and goal timing [80]. It is clear that in order to gain more insight in the consequences of goal-oriented care further research must primarily focus on how goal-oriented care is provided and can be supported. In order to investigate the potential benefits of goal-oriented care, research also needs to work on developing indicators of the goal-oriented process of care.

Strengths, limitations, and recommendations

The method of Walker and Avant provides a rigorous and systematic approach to refine the concept of goal-oriented care through the existing literature. A concept analysis is an exploration of an evolving concept which will need to be enriched by new knowledge. Therefore, it is influenced by contextual factors and must undergo adjustments to new implications and new insights based on further research. Since there is no specification given by Walker and Avant on how to conduct the literature review, we followed the guidelines from a scoping review as described by Levac (2010) [26]. The iterative process of adding new articles following the snowballing method is one of the strengths compared to other types of reviews. In this concept analysis, this led to a larger number of articles than the original search. A possible explanation for this might be that goal-oriented care was covered by synonyms or similar concepts that were not covered by the original search. Despite the systematic approach, a concept analysis does not comprise a quality assessment of the literature. However, it seemed to be an appropriate method to provide the knowledge needed to understand the different components of goal-oriented care in its entirety. The literature that was included in this study were only English written and peer reviewed. It would however be interesting to add also non-English literature to be able to capture more differences (e.g. cultural differences).

The literature search identified both original research papers and position papers. Some original research papers [3, 4, 28, 43, 46] evaluated goal-oriented care in clinical practice. These papers identified and described goal-oriented care as a stepwise intervention. Position papers [1, 12, 13, 40, 42] mostly described components of goal-oriented care rather than such a stepwise approach. The combination of both types gave more insight in the broad components of goal-oriented care.

This concept analysis could also be considered as a preliminary step to facilitate further research. One of the knowledge gaps revealed in this concept analysis is the lack of knowledge on what patients' goals are set, how goal-oriented care is delivered, and how it is best put into practice in both one-on-one interactions between patients and providers and in interprofessional collaboration. Regarding patients it is important to gain more insight in how they are preferably prepared for discussing their personal goals. In addition, the list of empirical referents made clear that a golden standard to evaluate goal-oriented care is missing. Initiating the development of an evaluation method could enable future intervention studies to gain more insight in the consequences of goal-oriented care and to make results comparable. Increasing insights from effective goal-oriented care could highlight its multiple benefits towards providers and policy makers. These results might also inform the healthcare system in which resources they need to facilitate goal-oriented care. This might be required to convince providers and policy makers of the benefits of goal-oriented care. A following step will first be to discuss these theoretical insights with patients and providers and deepen this information with insights from practices. Then, when goal-oriented care is well understood, a critical review can be set up to perform in-depth comparison between other concepts and frameworks. At this moment, we have (unfortunately) insufficient information to do this.

Goal-oriented care shows the potential to be a way forward for patients with chronic conditions and multimorbidity. However, further research is needed to further translate the current knowledge on the concept of goal-oriented care into a tangible workflow process of care that entails the three stages. This workflow should consists of tools to prepare patients and providers to offer goal-oriented care. This could contribute to finding a common ground in the goals and implementing goal-oriented care in practice. This workflow should include the skills and tools patients and providers need to implement goal-oriented care in practice.

Conclusion

This concept analysis aimed to translate the concept of goal-oriented care into a common understanding so providers can better understand and use this concept in clinical practice. The various literature on goal-oriented care, based on position and original research papers, showed a stepwise approach of three stages. Overall, the underpinning attributes of patients' context and patients' values form a philosophy of care to which the process must be reflected. Furthermore, both patients and the providers need to develop new skills in order to rethink the way care is provided. Patients must therefore be enabled to open up and reflect on their own agenda. Providers instead must learn to let go their own assumptions and solutions and communicate with their patients in a more balanced context. Based on the literature goal-oriented care shows the potential to improve patients' experience by listening to their needs and preferences, improve providers' well-being by the feeling of more satisfaction and reduce health care costs. Goal-oriented care could answer the challenges patients face with multiple care processes by initiating interprofessional collaboration. However, further research must focus on what and how goals are set, the translation of these findings into a workflow and must initiate the development of an evaluation method in order to investigate the effects of goal-oriented care processes on patients, providers and the health care system.

Acknowledgments

We are grateful for the partnership with the Primary Care Academy (academie-eerstelijn.be) and want to thank the King Baudouin Foundation and Fund Daniel De Coninck for the opportunity they offer us for conducting research and have impact on the primary care of Flanders, Belgium. The consortium of the Primary Care Academy consists of: Lead author: Roy Remmen – roy.remmen@uantwerpen.be - Department of Primary Care and Interdisciplinary Care, Faculty of Medicine and Health Sciences. University of Antwerp. Antwerp. Belgium; Emily Verté - Department of Primary Care and Interdisciplinary Care, Faculty of Medicine and Health Sciences. University of Antwerp. Antwerp.

Belgium, Department of Family Medicine and Chronic Care, Faculty of Medicine and Pharmacy. Vrije Universiteit Brussel. Brussel. Belgium; Muhammed Mustafa Sirimsi - Centre for research and innovation in care, Faculty of Medicine and Health Sciences. University of Antwerp. Antwerp. Belgium; Peter Van Bogaert - Workforce Management and Outcomes Research in Care, Faculty of Medicine and Health Sciences. University of Antwerp. Belgium; Hans De Loof - Laboratory of Physio pharmacology, Faculty of Pharmaceutical Biomedical and Veterinary Sciences. University of Antwerp. Belgium; Kris Van den Broeck - Department of Primary Care and Interdisciplinary Care, Faculty of Medicine and Health Sciences. University of Antwerp. Antwerp. Belgium.; Sibyl Anthierens - Department of Primary Care and Interdisciplinary Care, Faculty of Medicine and Health Sciences. University of Antwerp. Antwerp. Belgium; Ine Huybrechts - Department of Primary Care and Interdisciplinary Care, Faculty of Medicine and Health Sciences. University of Antwerp. Antwerp. Belgium.; Peter Raeymaeckers - Department of Sociology, Faculty of Social Sciences, Faculty of Social Sciences. University of Antwerp. Belgium; Veerle Buffel- Department of Sociology; centre for population, family and health, Faculty of Social Sciences. University of Antwerp. Belgium. ; Dirk Devroey- Department of Family Medicine and Chronic Care, Faculty of Medicine and Pharmacy. Vrije Universiteit Brussel. Brussel.; Bert Aertgeerts - Academic Centre for General Practice, Faculty of Medicine. KU Leuven. Leuven, Department of Public Health and Primary Care, Faculty of Medicine, KU Leuven. Leuven; Birgitte Schoenmakers - Department of Public Health and Primary Care, Faculty of Medicine, KU Leuven. Leuven. Belgium; Lotte Timmermans - Department of Public Health and Primary Care, Faculty of Medicine, KU Leuven. Leuven. Belgium.; Veerle Foulon - Department of Pharmaceutical and Pharmacological Sciences, Faculty Pharmaceutical Sciences. KU Leuven. Leuven. Belgium.; Anja Declerq - LUCAS-Centre for Care Research and Consultancy, Faculty of Social Sciences. KU Leuven. Leuven. Belgium.; Nick Verhaeghe - Research Group Social and Economic Policy and Social Inclusion, Research Institute for Work and Society. KU Leuven. Belgium.; Dominique Van de Velde Department of Rehabilitation Sciences, Occupational Therapy. Faculty of Medicine and Health Sciences. University of Ghent. Belgium., Department of Occupational Therapy. Artevelde University of

Applied Sciences. Ghent. Belgium.; Pauline Boeckxstaens - Department of Public Health and Primary Care, Faculty of Medicine and Health sciences. University of Ghent. Belgium.; An De Sutter - Department of Public Health and Primary Care, Faculty of Medicine and Health sciences. University of Ghent. Belgium.; Patricia De Vriendt - Department of Rehabilitation Sciences, Occupational Therapy. Faculty of Medicine and Health Sciences. University of Ghent. Belgium., Frailty in Ageing (FRIA) Research Group, Department of Gerontology and Mental Health and Wellbeing (MENT) research group, Faculty of Medicine and Pharmacy. Vrije Universiteit. Brussels. Belgium., Department of Occupational Therapy. Artevelde University of Applied Sciences. Ghent. Belgium.; Lies Lahousse - Department of Bioanalysis, Faculty of Pharmaceutical Sciences, Ghent University. Ghent. Belgium.; Peter Pype - Department of Public Health and Primary Care, Faculty of Medicine and Health sciences. University of Ghent. Belgium., End-of-Life Care Research Group, Faculty of Medicine and Health Sciences. Vrije Universiteit Brussel and Ghent University. Ghent. Belgium.; Dagje Boeykens- Department of Rehabilitation Sciences, Occupational Therapy. Faculty of Medicine and Health Sciences. University of Ghent. Belgium., Department of Public Health and Primary Care, Faculty of Medicine and Health sciences. University of Ghent. Belgium.; Ann Van Hecke - Department of Public Health and Primary Care, Faculty of Medicine and Health sciences. University of Ghent. Belgium., University Centre of Nursing and Midwifery, Faculty of Medicine and Health Sciences. University of Ghent. Belgium.; Peter Decat - Department of Public Health and Primary Care, Faculty of Medicine and Health sciences. University of Ghent. Belgium.; Rudi Roose - Department of Social Work and Social Pedagogy, Faculty of Psychology and Educational Sciences. University Ghent. Belgium.; Sandra Martin - Expertise Centre Health Innovation. University College Leuven-Limburg. Leuven. Belgium.; Erica Rutten - Expertise Centre Health Innovation. University College Leuven-Limburg. Leuven. Belgium.; Sam Pless - Expertise Centre Health Innovation. University College Leuven-Limburg. Leuven. Belgium.; Vanessa Gauwe - Department of Occupational Therapy. Artevelde University of Applied Sciences. Ghent. Belgium.; Didier Reynaert- E-QUAL, University College of Applied Sciences Ghent. Ghent. Belgium.; Leen Van Landschoot - Department of Nursing, University of Applied Sciences Ghent. Ghent.

Belgium.; Maja Lopez Hartmann - Department of Welfare and Health, Karel de Grote University of Applied Sciences and Arts. Antwerp. Belgium.; Tony Claeys- LiveLab, VIVES University of Applied Sciences. Kortrijk. Belgium.; Hilde Vandenhoudt - LiCalab, Thomas University of Applied Sciences. Turnhout. Belgium.; Kristel De Vliegher - Department of Nursing – homecare, White-Yellow Cross. Brussels. Belgium.; Susanne Op de Beeck - Flemish Patient Platform. Heverlee. Belgium.

References

1. Tinetti ME, Esterson J, Ferris R, Posner P, Blaum CS. Patient Priority-Directed Decision Making and Care for Older Adults with Multiple Chronic Conditions. Clinics in geriatric medicine. 2016;32(2):261-75. PubMed PMID: 27113145.
2. Rijken M, Bekkema N, Boeckxstaens P, Schellevis FG, De Maeseneer JM, Groenewegen PP. Chronic Disease Management Programmes: an adequate response to patients' needs? Health Expect. 2014;17(5):608-21. Epub 2012/06/21. doi: 10.1111/j.1369-7625.2012.00786.x. PubMed PMID: 22712877; PubMed Central PMCID: PMCPMC5060914.
3. Feder SL, Kiwak E, Costello D, Dindo L, Hern, ez-Bigos K, et al. Perspectives of Patients in Identifying Their Values-Based Health Priorities. Journal of the American Geriatrics Society. 2019 67(7):1379-85. PubMed PMID: 30844080.
4. Naik AD, Dindo LN, Van Liew JR, Hundt NE, Vo L, Hern, et al. Development of a Clinically Feasible Process for Identifying Individual Health Priorities. Journal of the American Geriatrics Society. 2018;66(10):1872-9. PubMed PMID: rayyan-47069467.
5. Cassell A, Edwards D, Harshfield A, Rhodes K, Brimicombe J, Payne R, et al. The epidemiology of multimorbidity in primary care: a retrospective cohort study. Br J Gen Pract. 2018;68(669):e245-e51. Epub 2018/03/14. doi: 10.3399/bjgp18X695465. PubMed PMID: 29530918; PubMed Central PMCID: PMCPMC5863678.
6. Berntsen G, Hoyem A, Lettrem I, Ruland C, Rumpsfeld M, Gammon D. A person-centered integrated care quality framework, based on a qualitative study of patients' evaluation of care in light of chronic care ideals. BMC Health Serv Res. 2018;18(1):479. Epub 2018/06/22. doi: 10.1186/s12913-018-3246-z. PubMed PMID: 29925357; PubMed Central PMCID: PMCPMC6011266.
7. Ploeg J, Matthew-Maich N, Fraser K, Dufour S, McAiney C, Kaasalainen S, et al. Managing multiple chronic conditions in the community: a Canadian qualitative study of the experiences of older adults, family caregivers and healthcare providers. BMC Geriatr. 2017;17(1):40.
8. Kramer MH, Bauer W, Dicker D, Durusu-Tanriover M, Ferreira F, Rigby SP, et al. The changing face of internal medicine: patient centred care. European journal of internal medicine. 25(2):125-7. PubMed PMID: 24472695.
9. Kuluski K, Peckham A, Gill A, Gagnon D, Wong-Cornall C, McKillop A, et al. What is Important to Older People with Multimorbidity and Their Caregivers? Identifying Attributes of Person Centered Care from the User Perspective. Int J Integr Care. 2019;19(3):4. Epub 2019/08/02. doi: 10.5334/ijic.4655. PubMed PMID: 31367203; PubMed Central PMCID: PMCPMC6659759.
10. Reuben DB, Tinetti ME. Goal-oriented patient care--an alternative health outcomes paradigm. The New England journal of medicine. 2012;366(9):777-9. Epub 2012/03/02. doi: 10.1056/NEJMp1113631. PubMed PMID: 22375966.
11. Kuipers SJ, Cramm JM, Nieboer AP. The importance of patient-centered care and co-creation of care for satisfaction with care and physical and social well-being of patients with multi-morbidity in the primary care setting. BMC Health Serv Res. 2019;19(1):1-9.
12. Reuben DB, Jennings LA. Putting Goal-Oriented Patient Care Into Practice. Journal of the American Geriatrics Society. 2019;67(7):1342-4. PubMed PMID: 30882888.
13. Mold JW, Blake GH, Becker LA. Goal-oriented medical care. Family medicine. 1991;23(1):46-51. Epub 1991/01/01. PubMed PMID: 2001782.
14. Sackett DL, Rosenberg WM, Gray JM, Haynes RB, Richardson WS. Evidence based medicine: what it is and what it isn't. British Medical Journal Publishing Group; 1996.
15. Baker A. Crossing the quality chasm: a new health system for the 21st century: British Medical Journal Publishing Group; 2001.

Formatted: Dutch (Belgium)

Formatted: Dutch (Belgium)

16. Elwyn G, Frosch D, Thomson R, Joseph-Williams N, Lloyd A, Kinnersley P, et al. Shared decision making: a model for clinical practice. Journal of general internal medicine. 2012;27(10):1361-7.

17. Vermunt N, Harmsen M, Westert GP, Olde Rikkert MGM, Faber MJ. Collaborative goal setting with elderly patients with chronic disease or multimorbidity: a systematic review. BMC Geriatr. 2017;17(1):167. Epub 2017/08/02. doi: 10.1186/s12877-017-0534-0. PubMed PMID: 28760149; PubMed Central PMCID: PMCPMC5537926.

18. Poitras ME, Maltais ME, Bestard-Denomme L, Stewart M, Fortin M. What are the effective elements in patient-centered and multimorbidity care? A scoping review. BMC Health Serv Res. 2018;18(1):446. Epub 2018/06/15. doi: 10.1186/s12913-018-3213-8. PubMed PMID: 29898713; PubMed Central PMCID: PMCPMC6001147.

19. Hobbs FR, Bankhead C, Mukhtar T, Stevens S, Perera-Salazar R, Holt T, et al. Clinical workload in UK primary care: a retrospective analysis of 100 million consultations in England, 2007–14. The Lancet. 2016;387(10035):2323-30.

~~20. IoMDoHCSCotFoP C, Donaldson M, Yordy K, Vanselow N. Defining primary care: an interim report. National Academy Press; 1994.~~

21. Institute of Medicine Committee on the Future of Primary C. In: Donaldson M, Yordy K, Vanselow N, editors. Defining Primary Care: An Interim Report. Washington (DC): National Academies Press (US)

Copyright 1994 by the National Academy of Sciences.; 1994.

22. Ellner AL, Phillips RS. The Coming Primary Care Revolution. J Gen Intern Med. 2017;32(4):380-6. Epub 2017/03/01. doi: 10.1007/s11606-016-3944-3. PubMed PMID: 28243869; PubMed Central PMCID: PMCPMC5377886.

23. Elwyn G, Durand MA, Song J, Aarts J, Barr PJ, Berger Z, et al. A three-talk model for shared decision making: multistage consultation process. bmj. 2017;359:j4891.

24. Lusk JM, Fater K. A concept analysis of patient-centered care. Nurs Forum. 2013;48(2):89-98. Epub 2013/04/23. doi: 10.1111/nuf.12019. PubMed PMID: 23600637.

25. Walker LOA, K.C. Strategies for theory constructing in nursing. Texas2019.

26. Levac D, Colquhoun H, O'Brien KK. Scoping studies: advancing the methodology. Implement Sci. 2010;5:69. Epub 2010/09/22. doi: 10.1186/1748-5908-5-69. PubMed PMID: 20854677; PubMed Central PMCID: PMCPMC2954944.

27. Mourad Ouzzani HH, Zbys Fedorowicz, and Ahmed Elmagarmid. Rayyan - a web and mobile app for systematic reviews. . Systematic Reviews. 2016;5(210). doi: 10.1186/s13643-016-0384-4.

28. Salter C, Shiner A, Lenaghan E, Murdoch J, Ford JA, Winterburn S, et al. Setting goals with patients living with multimorbidity: qualitative analysis of general practice consultations. Br J Gen Pract. 2019;69(684):e479-e88. Epub 2019/06/05. doi: 10.3399/bjgp19X704129. PubMed PMID: 31160370; PubMed Central PMCID: PMCPMC6592350.

29. Kessler D, Walker I, Sauve-Schenk K, Egan M. Goal setting dynamics that facilitate or impede a client-centered approach. Scandinavian journal of occupational therapy. 2018;26(5):315-24. PubMed PMID: 29671662.

30. De Maeseneer J, Boeckxstaens P. Care for noncommunicable diseases (NCDs): time for a paradigm-shift. World hospitals and health services : the official journal of the International Hospital Federation. 2011;47(4):30-3. PubMed PMID: 22619872.

31. Bayliss EA, Bonds DE, Boyd CM, Davis MM, Finke B, Fox MH, et al. Understanding the context of health for persons with multiple chronic conditions: moving from what is the matter to what matters. Ann Fam Med. 2014;12(3):260-9. Epub 2014/05/14. doi: 10.1370/afm.1643. PubMed PMID: 24821898; PubMed Central PMCID: PMCPMC4018375.

32. Berntsen GK, Gammon D, Steinsbekk A, Salamonsen A, Foss N, Ruland C, et al. How do we deal with multiple goals for care within an individual patient trajectory? A document content analysis of health service research papers on goals for care. BMJ open. 2015;5(12):e009403. Epub

Formatted: Dutch (Belgium)

Formatted: Dutch (Belgium)

2015/12/15. doi: 10.1136/bmjopen-2015-009403. PubMed PMID: 26656243; PubMed Central PMCID: PMCPMC4679896.

33. Blom J, Elzen WD, Houwelingen Anne HV, Heijmans M, Stijnen T, Van Den Hout W, et al. Effectiveness and cost-effectiveness of a proactive, goal-oriented, integrated care model in general practice for older people. A cluster randomised controlled trial: Integrated systematic care for older people-the ISCOPE study. Age and Ageing. 2016;45(1):30-41. doi: 10.1093/ageing/afv174.

34. Boeckxstaens, Willems, Lanssens, Decuypere, Brusselle, Kühlein, et al. A qualitative interpretation of challenges associated with helping patients with multiple chronic diseases identify their goals. Journal of comorbidity. 2016;6(2):120-6.

35. Mangin D, Stephen G, Bismah V, Risdon C. Making patient values visible in healthcare: a systematic review of tools to assess patient treatment priorities and preferences in the context of multimorbidity. BMJ open. 2016;6(6):e010903.

36. Schmidt K, Babac A, Pauer F, Damm K, von der Schulenburg JM. Measuring patients' priorities using the Analytic Hierarchy Process in comparison with Best-Worst-Scaling and rating cards: methodological aspects and ranking tasks. Health economics review. 2016;6(1):50. PubMed PMID: 27844450.

37. Franklin M, Lewis S, Willis K, Rogers A, Venville A, Smith L. Controlled, Constrained, or Flexible? How Self-Management Goals Are Shaped By Patient-Provider Interactions. Qualitative health research. 2019:1049732318774324. PubMed PMID: 29871583.

38. Tinetti M, Dindo L, Smith CD, Blaum C, Costello D, Ouellet G, et al. Challenges and strategies in patients' health priorities-aligned decision-making for older adults with multiple chronic conditions. PloS one. 2019;14(6):e0218249. PubMed PMID: 31181117.

39. Hurn J, Kneebone I, Cropley M. Goal setting as an outcome measure: A systematic review. Clinical rehabilitation. 2006;20(9):756-72. Epub 2006/09/29. doi: 10.1177/0269215506070793. PubMed PMID: 17005500.

40. Bodenheimer T, Handley MA. Goal-setting for behavior change in primary care: an exploration and status report. Patient education and counseling. 2009;76(2):174-80. Epub 2009/06/30. doi: 10.1016/j.pec.2009.06.001. PubMed PMID: 19560895.

41. Junius-Walker U, Stolberg D, Steinke P, Theile G, Hummers-Pradier E, Dierks M-L. Health and treatment priorities of older patients and their general practitioners: a cross-sectional study. Quality in primary care. 2011;19(2).

42. Lenzen SA, Daniels R, van Bokhoven MA, van der Weijden T, Beurskens A. Setting goals in chronic care: Shared decision making as self-management support by the family physician. The European journal of general practice. 2015;21(2):138-44. Epub 2014/12/30. doi: 10.3109/13814788.2014.973844. PubMed PMID: 25541857.

43. Steele Gray C, Wodchis WP, Upshur R, Cott C, McKinstry B, Mercer S, et al. Supporting Goal-Oriented Primary Health Care for Seniors with Complex Care Needs Using Mobile Technology: Evaluation and Implementation of the Health System Performance Research Network, Bridgepoint Electronic Patient Reported Outcome Tool. JMIR research protocols. 2016;5(2):e126. PubMed PMID: 27341765.

44. Kangovi S, Mitra N, Smith RA, Kulkarni R, Turr L, Huo H, et al. Decision-making and goal-setting in chronic disease management: Baseline findings of a randomized controlled trial. Patient Educ Couns. 2017;100(3):449-55. Epub 2016/10/09. doi: 10.1016/j.pec.2016.09.019. PubMed PMID: 27717532; PubMed Central PMCID: PMCPMC5437864.

45. Mold. Goal-directed health care: redefining health and health care in the era of value-based care. Cureus 2017;9(2).

46. Schellinger SE, Anderson EW, Frazer MS, Cain CL. Patient Self-Defined Goals: Essentials of Person-Centered Care for Serious Illness. The American journal of hospice & palliative care. 2018;35(1):159-65. Epub 2017/03/24. doi: 10.1177/1049909117699600. PubMed PMID: 28330379; PubMed Central PMCID: PMCPMC5704564.

47. Vermunt NP, Harmsen M, Elwyn G, Westert GP, Burgers JS, Olde Rikkert MG, et al. A three-goal model for patients with multimorbidity: A qualitative approach. Health expectations : an

Formatted: Dutch (Belgium)

Formatted: Dutch (Belgium)

Formatted: Dutch (Belgium)

Formatted: Dutch (Belgium)

international journal of public participation in health care and health policy. 2018;21(2):528-38. PubMed PMID: 29193557.

48. de Groot E, Schonrock-Adema J, Zwart D, Damoiseaux R, Van den Bogerd K, Diemers A, et al. Learning from patients about patient-centredness: A realist review: BEME Guide No. 60. Medical teacher. 2019:1-13. Epub 2019/12/20. doi: 10.1080/0142159x.2019.1695767. PubMed PMID: 31852313.

49. Kuluski K, Guilcher SJT. Toward a Person-Centred Learning Health System: Understanding Value from the Perspectives of Patients and Caregivers. HealthcarePapers. 2019;18(4):36-46. Epub 2020/01/05. doi: 10.12927/hcpap.2019.26030. PubMed PMID: 31901067.

50. Tinetti ME, Naik AD, Dindo L, Costello DM, Esterson J, Geda M, et al. Association of Patient Priorities-Aligned Decision-Making with Patient Outcomes and Ambulatory Health Care Burden among Older Adults with Multiple Chronic Conditions: A Nonrandomized Clinical Trial. JAMA Internal Medicine. 2019. PubMed PMID: rayyan-47069570.

51. Eckhoff DO, Weiss J. Goal setting: A concept analysis. Nursing forum. 2020. Epub 2020/01/07. doi: 10.1111/nuf.12426. PubMed PMID: 31902133.

~~52. Purkaple BA, Nagykaldi ZJ, Allahyar A, Todd R, Mold JW. Physicians' Response to Patients' Quality-of-Life Goals. The Journal of the American Board of Family Medicine. 2020;33(1):71. doi: 10.3122/jabfm.2020.01.190169.~~

53. Sathanapally H, Sidhu M, Fahami R, Gillies C, Kadam U, Davies MJ, et al. Priorities of patients with multimorbidity and of clinicians regarding treatment and health outcomes: a systematic mixed studies review. BMJ open. 2020;10(2):e033445. Epub 2020/02/14. doi: 10.1136/bmjopen-2019-033445. PubMed PMID: 32051314.

54. Reuben DB, Tinetti ME. Goal-oriented patient care - An alternative health outcomes paradigm. New England Journal of Medicine. 2012;366(9):777-9. doi: 10.1056/NEJMp1113631.

55. Heisler M, Bouknight RR, Hayward RA, Smith DM, Kerr EA. The relative importance of physician communication, participatory decision making, and patient understanding in diabetes self-management. Journal of general internal medicine. 2002;17(4):243-52.

56. Purkaple BA, Nagykaldi ZJ, Allahyar A, Todd R, Mold JW. Physicians' Response to Patients' Quality-of-Life Goals. J Am Board Fam Med. 2020;33(1):71-9. Epub 2020/01/08. doi: 10.3122/jabfm.2020.01.190169. PubMed PMID: 31907248.

57. Boyd CM, Darer J, Boult C, Fried LP, Boult L, Wu AW. Clinical practice guidelines and quality of care for older patients with multiple comorbid diseases: implications for pay for performance. Jama. 2005;294(6):716-24. Epub 2005/08/11. doi: 10.1001/jama.294.6.716. PubMed PMID: 16091574.

58. Mangin D, Stephen G, Bismah V, Risdon C. Making patient values visible in healthcare: A systematic review of tools to assess patient treatment priorities and preferences in the context of multimorbidity. BMJ Open. 2016;6(6). doi: 10.1136/bmjopen-2015-010903.

59. Vermunt NP, Harmsen M, Westert GP, Rikkert MGO, Faber MJ. Collaborative goal setting with elderly patients with chronic disease or multimorbidity: a systematic review. BMC geriatrics. 2017;17(1):167.

60. Boyd CM, Wolff JL, Giovannetti E, Reider L, Weiss C, Xue Q-l, et al. Health care task difficulty among older adults with multimorbidity. Medical care. 2014;52(0 3):S118.

61. Locke EA, Latham GP. A theory of goal setting & task performance: Prentice-Hall, Inc; 1990.

62. Callahan EJ, Bertakis KD. Development and validation of the Davis Observation Code. Fam Med. 1991;23(1):19-24. Epub 1991/01/01. PubMed PMID: 2001776.

63. Åsenlöf P, Siljebäck K. The patient goal priority questionnaire is moderately reproducible in people with persistent musculoskeletal pain. Physical therapy. 2009;89(11):1226-34.

64. Melville LL, Baltic TA, Bettcher TW, Nelson DL. Patients' perspectives on the self-identified goals assessment. Am J Occup Ther. 2002;56(6):650-9. Epub 2002/12/03. doi: 10.5014/ajot.56.6.650. PubMed PMID: 12458857.

65. Dedding C, Cardol M, Eyssen IC, Dekker J, Beelen A. Validity of the Canadian Occupational Performance Measure: a client-centred outcome measurement. Clinical rehabilitation. 2004;18(6):660-7. Epub 2004/10/12. doi: 10.1191/0269215504cr746oa. PubMed PMID: 15473118.

Formatted: Dutch (Belgium)

Formatted: Dutch (Belgium)

Formatted: Dutch (Belgium)

66. Fried TR, Tinetti M, Agostini J, Iannone L, Towle V. Health outcome prioritization to elicit preferences of older persons with multiple health conditions. Patient education and counseling. 2011;83(2):278-82.
67. Steele Gray C, Gill A, Khan AI, Hans PK, Kuluski K, Cott C. The Electronic Patient Reported Outcome Tool: Testing Usability and Feasibility of a Mobile App and Portal to Support Care for Patients With Complex Chronic Disease and Disability in Primary Care Settings. JMIR Mhealth Uhealth. 2016;4(2):e58. Epub 2016/06/04. doi: 10.2196/mhealth.5331. PubMed PMID: 27256035; PubMed Central PMCID: PMCPMC4911509.
68. Toto PE, Skidmore ER, Terhorst L, Rosen J, Weiner DK. Goal Attainment Scaling (GAS) in geriatric primary care: a feasibility study. Arch Gerontol Geriatr. 2015;60(1):16-21. Epub 2014/12/04. doi: 10.1016/j.archger.2014.10.022. PubMed PMID: 25465505.
69. Glasgow RE, Wagner EH, Schaefer J, Mahoney LD, Reid RJ, Greene SM. Development and validation of the Patient Assessment of Chronic Illness Care (PACIC). Med Care. 2005;43(5):436-44. Epub 2005/04/20. doi: 10.1097/01.mlr.0000160375.47920.8c. PubMed PMID: 15838407.
70. Teal CR, Haidet P, Balasubramanyam AS, Rodriguez E, Naik AD. Measuring the quality of patients' goals and action plans: development and validation of a novel tool. BMC medical informatics and decision making. 2012;12:152. Epub 2012/12/29. doi: 10.1186/1472-6947-12-152. PubMed PMID: 23270422; PubMed Central PMCID: PMCPMC3544573.
71. Etz RS, Zyzanski SJ, Gonzalez MM, Reves SR, O'Neal JP, Stange KC. A New Comprehensive Measure of High-Value Aspects of Primary Care. Annals of family medicine. 2019;17(3):221-30. PubMed PMID: rayyan-47069321.
72. Adam P, Murphy CF, Dierich M, Hager KD. Seven Years of Teaching Communication With the Patient-Centered Observation Form. Fam Med. 2018;50(2):132-7. Epub 2018/02/13. doi: 10.22454/FamMed.2018.516713. PubMed PMID: 29432629.
73. Murdoch J, Salter C, Ford J, Lenaghan E, Shiner A, Steel N. The "unknown territory" of goal-setting: Negotiating a novel interactional activity within primary care doctor-patient consultations for patients with multiple chronic conditions. Soc Sci Med. 2020;256:113040. Epub 2020/05/31. doi: 10.1016/j.socscimed.2020.113040. PubMed PMID: 32473530; PubMed Central PMCID: PMCPMC7306159.
74. Singh Ospina N, Phillips KA, Rodriguez-Gutierrez R, Castaneda-Guarderas A, Gionfriddo MR, Branda ME, et al. Eliciting the Patient's Agenda- Secondary Analysis of Recorded Clinical Encounters. J Gen Intern Med. 2019;34(1):36-40. Epub 2018/07/04. doi: 10.1007/s11606-018-4540-5. PubMed PMID: 29968051; PubMed Central PMCID: PMCPMC6318197.
75. Hersh L, Salzman B, Snyderman D. Health Literacy in Primary Care Practice. Am Fam Physician. 2015;92(2):118-24. Epub 2015/07/16. PubMed PMID: 26176370.
76. Sine DM, Sharpe VA. Ethics, risk, and patient-centered care: how collaboration between clinical ethicists and risk management leads to respectful patient care. J Healthc Risk Manag. 2011;31(1):32-7. Epub 2011/07/28. doi: 10.1002/jhrm.20077. PubMed PMID: 21793115.
77. Tinetti ME, Bogardus Jr ST, Agostini JV. Potential pitfalls of disease-specific guidelines for patients with multiple conditions. N Engl j Med. 2004;351(27):2870-4.
78. Voigt I, Wrede J, Diederichs-Egidi H, Dierks ML, Junius-Walker U. Priority setting in general practice: health priorities of older patients differ from treatment priorities of their physicians. Croatian medical journal. 2010;51(6):483-92. Epub 2010/12/17. doi: 10.3325/cmj.2010.51.483. PubMed PMID: 21162160; PubMed Central PMCID: PMCPMC3012393.
79. Etz R, Stange KC. Measuring what matters in primary care. Global Advances in Health and Medicine. 2018;7:263. doi: 10.1177/2164956118773837.
80. Young RA, Roberts RG, Holden RJ. The Challenges of Measuring, Improving, and Reporting Quality in Primary Care. Annals of family medicine. 2017;15(2):175-82. Epub 2017/03/16. doi: 10.1370/afm.2014. PubMed PMID: 28289120; PubMed Central PMCID: PMCPMC5348238.

Supporting information

S1 Table 1. Overview preliminary version attributes.

S2 Table 2. Overview training.

S1 File. Prisma scr checklist.

~~S1 Table 1. Overview preliminary version attributes.~~

~~S2 Table 2. Overview of training.~~

~~S1 Fig. Flow Chart.~~

~~S3 Table. Overview of the included articles.~~

Dear Editor
Dear reviewers

We appreciate your extensive and constructive feedback on our manuscript.
We aimed to respond all of your remarks by point to point answers and changes in the manuscript.
Hopefully they will meet your expectations, otherwise we will make further adjustments.

Sincerely
Dagje Boeykens, on behalf of the co-authors

	**Comments from the editor**	**Response**	**Changes in the manuscript**
1	Please ensure that your manuscript meets PLOS ONE's style requirements, including those for file naming.	Adjustments have been made to meet the PLOS ONE’s style requirements.	Changes in the titles and references to figures and tables.
2	During the internal evaluation of the manuscript, we feel that this study fits within the scope of a Scoping Review. As such we please consider modifying your title to specify this. For instance "Goal-oriented care in primary care: a scoping review and concept analysis.	Thank you for this comment and we do agree with the fact that no specifications are given regarding the way how the literature search should be performed in a concept analysis according to Walker and Avant. From this perspective we can agree with the comment to think about adding ‘scoping review’ to the title because there are indeed some similarities. However, a scoping review and a concept-analysis are two different methodologies and we did not follow the guidelines from a scoping review (e.g. guidelines Joan Briggs institute or Arksey and O’Malley), but the guidelines from Walker and Avant. So we are a little bit puzzled by this request, in the one hand we would like to meet your comment, but on the other hand we feel that we cannot change the title and method because we did not perform a scoping review as it should have been done. From this	Our primary option would be not to change the title and keep the original title.<br><br>In case we should change, we would suggest the following title,<br><br>Goal-oriented care ***for patients with chronic conditions or multimorbidity: a scoping review*** and concept analysis<br><br>In the method section we added the following lines:<br><br>***(L173 – method: step3) As the method of a concept analysis does not specify how the literature search has to be performed, this search was based on the method of a scoping review described by Levac (2010)*** [1]

		perspective, we would like to suggest no changes to the title (but perhaps add it as a key word).<br><br>However, if this comment is a breaking point for being accepted as publication, and from the aspect to be identifiable by possible readers as a review article we can perfectly follow this comment. The way we have performed our literature search is similar to the way Levac describes how a literature study should be performed in scoping reviews. So, from this perspective we can follow to add 'scoping review' to the title, but then we suggest to add a few words to the method section and to the discussion section. In that case, please let us know.<br><br>Levac D, Colquhoun H, O'Brien KK. Scoping studies: advancing the methodology. Implement Sci. 2010;5:69.	***Related concepts such as patient-centered care, value-based care, etc. were not included as the method of concept analysis prescribes to deepen all the attributes of one concept.***<br><br>And add in the discussion section the following lines in the limitation section:<br><br>***(L666 – limitations) Since there is no specification given by Walker and Avant on how to conduct the literature review, we followed the guideline from a scoping review as described by Levac (2010). This could have influenced the results.***
3	In addition please provide a PRIMSA flow chart as Figure 1, list of studies as Table 1, and a completed PRISMA-Scr checklist as Supporting File	A PRISMA-Scr has been completed and also added to the supporting files. For us it was not that clear if the flow chart and tables have to be replaced to the supporting files, so if we need to do that, please let us know.	A completed Prisma-Scr checklist has been added and presented at the supporting files.
4	Finally, please include in your Methods section the date ranges over which you conducted the literature search.	The date range has been added in the method section.	***(L 173 – select the literature): The literature search was conducted between January 2020 and April 2020***. A preliminary combination of…
5	One of the noted authors is a group or consortium Primary Care Academy. In addition to naming the author group, please list the	A list of the individual authors and affiliations has been made and added to the acknowledgments. Prof. dr. Roy Remmen	/

	individual authors and affiliations within this group in the acknowledgments section of your manuscript. Please also indicate clearly a lead author for this group along with a contact email address.	(roy.remmen@uantwerpen.be) has been indicated as the lead author of the consortium.	
6	Please review your reference list to ensure that it is complete and correct. If you have cited papers that have been retracted, please include the rationale for doing so in the manuscript text, or remove these references and replace them with relevant current references. If you need to cite a retracted article, indicate the article's retracted status in the References list and also include a citation and full reference for the retraction notice.	The references list is checked and adjustments have been made.	21. Institute of Medicine Committee on the Future of Primary C. In: Donaldson M, Yordy K, Vanselow N, editors. Defining Primary Care: An Interim Report. Washington (DC): National Academies Press (US)<br>Copyright 1994 by the National Academy of Sciences.; 1994.<br><br>36. Purkaple BA, Nagykaldi ZJ, Allahyar A, Todd R, Mold JW. Physicians' Response to Patients' Quality-of-Life Goals. J Am Board Fam Med. 2020;33(1):71-9.

Comments from reviewer 1		Response	Changes in the manuscript
Title			
7	No mention is made of multimorbidity, a key concept within the paper. It should be added in.	Indeed, the concept analysis also focusses on chronic conditions/ multimorbidity, so this is added to the title.	Goal-oriented care **for patients with chronic conditions and multimorbidity** in primary care: a concept analysis.
Introduction			
8	An assumption is made throughout that goal oriented care is likely to be better - can the authors provide any effectiveness data related to this topic? There are a number of trials in multimorbidity in which a goal setting approach is used. Likewise in the abstract intro.	Goal-oriented care has indeed some potential benefits. As the outcomes of the effectiveness studies were considered as results of the analyzing process under 'consequences' of the result sections they were not specifically mentioned in the introduction. The outcomes of effectiveness studies have also been discussed in the 'discussion section' under the paragraph of 'consequences'. To meet the comment, we have given more attention to the potential outcomes of meeting the patients' needs in the introduction. Though, it should be noticed that more research is needed to elaborate on the effectiveness data on this topic.	(L86 – introduction) A possible way to overcome many of the challenges is to shift care back from 'what's the matter with the patient' to 'what matters to the patient'. It creates health care processes in which patients' needs are actively sought and met [2]. ***Meeting those patients' needs and tailoring care more to what patients want in a co-creation process is assumed to result in better social well-being, physical well-being, and satisfaction for patients and healthcare providers [3].***
9	Disease-specific care is positioned as opposite to goal oriented care. However, within a number of chronic disease management programs, goal setting plays a large part (although the patient centeredness of this may be debatable). Perhaps the authors need to more clearly distinguish between goal setting in care and goal-oriented care, which seems to be broader in their analysis.	Indeed, there is a need to distinguish between goal setting and goal-oriented care and we recognize that goal-setting plays a large part in the entire organization of (chronic) care.<br><br>This comment has been addressed in the introduction and in the discussion section.	***(L114 – introduction) Some healthcare providers might already assume that they practice goal-oriented care spontaneously, but there still is a lack of underpinning knowledge and guidance on how to provide goal-oriented care to patients The main pitfall in most of these goal-setting activities is that the goals are not necessarily related to the patients' needs and preferences while in goal-oriented care these patients' needs and preferences are put on the forefront and not necessarily health-related [4, 5]. From this perspective, goal-setting and goal-oriented care should be***

			taken together and focusing on the patient's needs and preferences.<br><br>***(L580 – discussion) In goal-oriented care it seems important to set goals based on the patients' needs and preferences (e.g; I want to take my grandchildren to school), while in other chronic disease management programs the emphasis is mainly still on health-related goals [6] (e.g. I want the patient to be able to walk without pain).***
10	Likewise, other related concepts such as shared decision making and patient centered care are only briefly touched upon and in either the intro or discussion or both need to be discussed as to how they relate to goal oriented care.	This is an important remark as we did not elaborate on the related concepts. We consider this concept analysis as a first and main step in learning more about goal-oriented care. The method of a concept analysis did not allow us to make an overview of the differences and similarities of the related concepts.<br>Although, to meet this valuable comment we have added more information in the introduction.	(L93 – introduction) The concept of goal-oriented care has been launched and mentioned for the first time in 1991 by Mold who proposed the concept as an alternative way of providing care [7]. Later on, in 2012, Reuben and Tinetti took the concept of goal-oriented care a step forward by stating that care "must above all consider patients' preferred outcomes" [8]. The focus on setting goals based on the patients' needs and preferences rather than on health-related outcomes became one of the main novelties in chronic disease management [6]. ***Not only could goal-oriented care be proposed as an important paradigm to overcome some of the new challenges for chronical patients [2], it might also corresponded to the original concept of evidence based medicine (EBM) [9]. EBM was first published by Sackett in 1996 who described three key components: 1. best external evidence, 2. individual clinical expertise, and 3. patients' values and expectations [9]. Since the first description of EBM, multiple approaches***

			and paradigms has been developed to compromise between those three components [10]. For example, patient-centered care (PCC), which is already a well-known and widely used concept, is defined as "providing care that is respectful of, and responsive to individual patient preferences, needs, and values and ensuring that patients values guides all clinical decisions" [10]. Shared-decision making, on the other hand, also strives to share evidence and engage patients in care as it is "an approach where clinicians and patients share the best available evidence when faced with the task of making decisions, and where patients are supported to consider options, and to achieve informed preferences" [11]. Goal-oriented care is proposed as a promising healthcare paradigm and approach to operationalize EBM and return to where it all started [8]. However, in contrast to the other approaches and paradigms, goal-oriented care is ill defined. Developing a common understanding on the concept could potentially contribute to the clarification and in-depth comparison between the related concepts and eventually lead to better use in clinical practice. However, some healthcare providers might already assume that they practice goal-oriented care spontaneously, but there still is a lack of underpinning knowledge and guidance on how to provide goal-oriented care to patients [4, 5]. As a first step in exploring the potential of goal-oriented care in chronic care, it is important to gain in-depth knowledge

			on what goal-oriented care is about and how it can be generally described.<br>As goal-oriented care could be well-suited in primary care, as this context is often the linchpin for patients with chronic conditions, this will be the focus of this study [12]. This study aimed to describe a structured approach to deepen the concept of goal-oriented care for patients with chronic conditions or multimorbidity in the primary care context.
11	Primary care is seen as the main focus of this paper, which makes sense, but there is little detail on collaboration and how goal oriented care would fit with collaborative approaches and whose responsibility in the primary care team it could/should be.	This is an important remark that we questioned ourselves already several times. Overall, the current literature pays little attention on (interprofessional) collaboration and how goal-oriented care could potentially facilitate this collaboration. Only one study (Vermunt et al.) mentioned who should take the lead during the process, suggesting different professionals including to be GP, nurse, practice nurse, psychological wellbeing practitioner. This information is now more specified. In a study of Mold it was suggested that occupational therapists as probably the most 'goal-oriented' profession without specifying that this profession should take the responsibility [13]	***(L397 – results: care plan is based on patients' needs and preferences) To guide this interprofessional review, no specification was given about which profile would be the best fit for having the lead. Vermunt et al. (2017) illustrated this as they found variation in who (e.g. GP, nurse, practice nurse, psychological wellbeing practitioner) should contribute to goal-setting [4].***
Method			
12	I appreciate the literature searches are iterative but it would be useful to know the range of dates searched or at the very least the date of the most recent search, in order to contextualize the point at which this was done.	The date range is added to step 3 of the method section.	***(L173 – method: step 3) The literature search was conducted between January 2020 and April 2020***. A preliminary combination of…

13	'confirmed the first results' unclear what this means - no new studies found? or no new concepts identified?	Indeed, this was unclear. We meant that no new studies were identified. Further details on this method part has been added.	(L183 – method: step3) In a second phase, CINAHL, OTSeeker, PsycINFO and Web of Science were consulted and confirmed the first results ***as no new studies were identified.***
14	In the inclusion criteria, it is unclear how much of a focus was needed on goal oriented care to be included - was there a minimum level of discussion or characterization required? How many authors determined it had sufficient focus? To me this would seem to be difficult to judge.	It was indeed difficult to judge if a study should be included or not as goal-oriented care is a poorly defined concept (this was the reason why we conducted this concept analysis in the first place). However, by predefining the inclusion criteria we tried to make this choice more objective. Further, the studies were discussed with the co-authors to evaluate if they could contribute to the theoretical building of an understanding on goal-oriented care. Each co-author had an open and reflexive view to the articles so the risk of bias was reduced. We also strived for consensus with the authors in several discussion round that were held to increase reliability of the inclusion process.<br>We added this in the method section.	(L 217 – method: step 4) The determination of the attributes started with a discussion of four key articles [14-17] selected by the first author based on the divers approaches of goal-oriented care ***and presented to the research group.***<br><br>***(L223 – method: step 4) In a second phase, new articles were added and analyzed based on the same method as the key articles until all relevant literature (based on the inclusion criteria) was included.***
15	Clarify that all paper types were included.	A sentence is added to make this more clear.	***(L214 – method: step 3) There was no restriction by study design to gain as much insight in goal-oriented from different perspectives.***
16	"a chronic condition"? This paper is focused on multimorbidity so surely it should be >1?	The paper is focused on both, just one chronic condition or multiple conditions (multimorbidity). Changes have been made.	(L206 – method: step 3) and (c) focusing on patients with ***one or more chronic conditions.***
17	Papers included were English only. It would be interesting to know how many papers were in another language as it seems like that there may be cultural differences that could not be picked up?	This will indeed be interesting to identify potential cultural differences. During the literature search, no specific filters were used. This allowed us to, indeed, have knowledge about papers in other languages and eventually potential cultural differences.	In the limitation section we have added the following:<br><br>***(L674 – limitations) The literature that was included in this study were only English written and peer reviewed. It would however be***

		However, no studies in other languages could be found by means of our search strategy. Though, English written papers could also provide insight in differences relating to context and culture, but the included articles related to Western countries so at this moment no differences could be identified. We added this in the limitation section.	***interesting to add also non-English papers to be able to capture more differences (e.g. cultural differences)***
Results			
18	Results: flow chart too low res to view.	A new flow chart has been made with better resolution. Following the editor's suggestion this flowchart has now been placed in the supplementary files.	
19	Table 2 is clear but would appreciate adding further details on methods, plus perhaps contribution it made to the concept analysis (could add in numbers from table 3 to indicate where contributed to?). Some study methods are blank? I don't see the relevance of journal.	Table 2 is supplemented with more information about the method.<br>Further, references are added in Table 3 so the link to the corresponding article is more clear.	Table 2 is completed and references are added in Table 3.
20	p18 patient's needs and preferences... within this section I wondered if any of the papers had picked up on expectation management, which would seem to be an important part of the process which is not really considered here.	It is an interesting remark to link the paragraph of the patients' needs and preferences to expectation management. However, after rereading the included articles none of them relate to that. What we see in the literature that has been published after the literature search was finalized, is that it is important to find the underlying values of goals patients set [18]. We agree that this is an important issue, but based on the literature we have reviewed we cannot provide an answer and therefore we did not make any changes in the manuscript.	No changes have been made in the manuscript.

21	Additionally on page 19 I wondered what the implications were when people's goals went beyond the remit of healthcare professionals in terms of their aims or their barriers and facilitators (e.g. finances, caring responsibilities) and whether the responsibility should be on the HCP to address these kind of issues as part of holistic care or the patient as part of their own self-management? This could also be picked up on the discussion and implications.	Thank you for this comment. Some authors, however limited, touched upon this issue. We have addressed this comment in the discussion section.	***(L394 – results: care plan is based on patients' needs and preferences) In case that providers are confronted with patients' goals that are out of their own scope, they could benefit from an interprofessional review as they are enabled to discuss with and hand over to others with the needed expertise. This could improve the coordination of the care plans between the different providers and facilitate integrated care delivery [6, 17, 19]. To guide this interprofessional review, no specification was given about which profile would be the best fit for having the lead. Vermunt et al. (2017) illustrated this as they found variation in who (e.g. GP, nurse, practice nurse, psychological wellbeing practitioner) should contribute to goal-setting [4].***<br><br>(L 631 – discussion) Therefore training should also include the interprofessional perspective to facilitate a uniform attitude towards the patients' goals and principles of goal-oriented care in the entire team. ***This will potential support providers to learn from and with each other's' expertise and enable discussion between them in case that, for example, patients set goals that our out of the remit of the provider.***
22	It is somewhat unclear who should be leading/doing the care plan. Whilst an interprofessional approach is emphasized, there is little detail on whether a specific role (e.g. care coordinator or similar) would be required	We do agree with this comment and agree that interprofessional collaboration is an important topic to relate with goal-oriented care. Besides Vermunt (2010) little attention is given in the contemporary literature describing the concept	***(L394 – results: care plan is based on patients' needs and preferences) In case that providers are confronted with patients' goals that are out of their own scope, they could benefit from an interprofessional review as they are enabled to***

	for this process to take place.	of goal oriented care who should take the lead or responsibility in the process. We have added a few lines to address this issue.	***discuss with and hand over to other providers with the needed expertise. This could improve the coordination of the care plans between the different providers and facilitate integrated care delivery [6, 17, 19]. To guide this interprofessional review, no specification was given about which profile would be the best fit for having the lead. Vermunt et al. illustrated this as they found variation in who (e.g. GP, nurse, practice nurse, psychological wellbeing practitioner) should contribute to goal-setting [4].***
23	There's also little differentiation throughout as to whether the goals set are meant to be actioned by the patient or providers, which is an important consideration when considering measurement and review and goal level.	This is an important remark as this will be one of the main concerns of providers and patients to apply goal-oriented care. However, in the current (included) literature no specification was given regarding the person who should action the goals. What we saw in the literature is that the provider should support patients in undertaking action to achieve their goals, but that they should let the patients in their own responsibility in whether or not striving for their goals. It is however important to notice that in goal-oriented care it is most of all important to identify the patients' needs and preferences to guide the conversations and interaction rather than focusing on achieving the patients' goals.	(L: 699 – strengths, limitations, and recommendations) Goal-oriented care shows the potential to be a way forward for patients with chronic conditions and multimorbidity. However, further research is needed to translate the current knowledge on the concept of goal-oriented care into a tangible workflow process of care that entails the three stages. This workflow should consists of tools to prepare patients and providers to offer goal-oriented care. This could contribute to finding a common ground in the goals and implementing goal-oriented care in practice.
24	Throughout it would be good to know when a paper is quoted, the type of data/expertise it is coming from and whether these characterizations are consistent across methods (e.g. whether characterized in the same way from a detailed conversational analysis	Thank you for this suggestion. It indeed is interesting to get an overview of the included articles and the method they have used. Therefore we have integrated the previous comment (18) to complete Table 2 and make the link with Table 3. Together with a	***(L257 – method: step 3) These articles represented a broad range of study types: 4 systematic reviews, 4 experimental studies (e.g. randomized controlled trial), 13 qualitative studies, 3 survey studies, 1 concept analysis, 1 methodology paper, 4 reviews, 2 position***

	perspective vs an overview paper)	descriptive overview of the diverse study types we tried to provide this overview.	***papers, 1 background paper, 1 status report, 1 commentary, 1 opinion paper, and 1 perspective.***<br><br>***+ Table 2 and 3***
25	The case boxes are cut off so I can't comment on them. Box 3 states 'her physician does not allow' - does this mean he is preventing Mary from travelling, which does not seem like something he would be able to, or is this perhaps a slight miswording?	Our excuses for the missing parts of the cases. Also reviewer 2,3, and 4 comment (32 & 37) that the case of Mary could seem slightly unrealistic. To meet these comments, the case has been rewritten.	Mary is a 40-year old mother of two young children and dealing with obesity since her childhood. ***Due to her weight, she has a lot of joints pain and is short of breath which limits her exercising capacity. Her children would love nothing more than their mother play with them. Unfortunately, she is not able to play soccer or jump on the trampoline because of the pain. The pain becomes too much for her and after long hesitation she discusses this with her physician. The only thing she wants is to play and interact with her children as painless as possible and therefore asks her physician to prescribe some medication. Her physician does not support medication, but instructs her to first strive for a healthy weight as a solution to relieve the pain. This is not aligned with the wishes of Mary who only wanted a short-term solution to be able to play with her children.*** In the end, she leaves the consultation room with a referral to a dietitian and sport coach.
26	In the patient preparedness part, could any conclusions be drawn about the best ways to prepare patients?	Thank you for this interesting remark, but unfortunately no in-depth information was given in the literature regarding the way how patients should be prepared. It is something we also questioned ourselves. We added specifically in the discussion section that this will be subject to further research.	(L683 – strengths, limitations, and recommendations) One of the knowledge gaps revealed in this concept analysis is the lack of knowledge on what patients' goals are set, how goal-oriented care is delivered, and how it is best put into practice in both one-on-one interactions between patients and providers

			and in interprofessional collaboration.<br>***Regarding patients it is important to gain more insight in how they are preferably prepared for discussing their personal goals.***
Discussion			
27	Should be a bit wider not just in terms of the concept but how it fits in more widely. There is overall a lack of discussion around the issues of provider-provider collaboration (for example frequently care plans however holistically developed are not shared across providers, particularly those with different IT systems) and healthcare professional time (which would seem to be the major barrier)	This is an important remark as the provider-provider collaboration and the aspect of time of healthcare professionals seem to be important aspects that relate to goal-oriented care. However, no included articles mentioned something about collaboration nor time. It seems logic that providers need sufficient time to have a goal-oriented care conversation, etc. but for this discussion we have chosen to focus on what is described in the literature.	(L683 – strengths, limitations, and recommendations) One of the knowledge gaps revealed in this concept analysis is the lack of knowledge on what patients' goals are set, how goal-oriented care is delivered, ***and how it is best put into practice in both one-on-one interactions between patients and providers and in interprofessional collaboration.*** Regarding patients it is important to gain more insight in how they are preferably prepared for discussing their personal goals.<br><br>(L689 – strengths, limitations, and recommendations) Initiating the development of an evaluation method could enable future intervention studies to gain more insight in the consequences of goal-oriented care and to make results comparable. ***This future research could provide insight in how effective goal-oriented care could potentially be which will be important to convince providers and policy makers of the benefits. These results might also inform the healthcare system in which resources they need to facilitate goal-oriented care***.
28	Couple of English corrections needed throughout: L109 chronical should be	The corrections has been made.	

	chronically, L211 independency should be independence		

Comments from reviewer 2		Response	Changes in the manuscript
Results			
29	As the authors mentioned, there are similar concepts with Goal-oriented care. For example, Patient-Centered Clinical Method, Value-Based Practice or Expert Generalist Practice have proposed similar frameworks in primary care settings. I think the authors need to mention the difference between Goal-oriented care and the other concepts and why the authors chose Goal-oriented care in the Background and Discussion section. This is important for readers who are not familiar with Goal-oriented care.	This is an important remark as we did not elaborate on the related concepts. We consider this concept analysis as a first and main step in learning more about goal-oriented care. Based on this methodology and the results we were not able to make an overview of the differences and similarities of other concepts. Therefore, a critical review can be set up to perform in-depth comparison between the related concepts.<br><br>Also reviewer 1 (comment 9,10) and 3 (comment 34,35) have commented on the absence of the link with the related concepts. To address these comments, we have added more clarification on why we have chosen for the concept of goal-oriented care and where the other concepts could be placed. In addition, we addressed this gap in the recommendations.	(L95 – introduction) The concept of goal-oriented care has been launched and mentioned for the first time in 1991 by Mold who proposed the concept as an alternative way of providing care [7]. Later on, in 2012, Reuben and Tinetti took the concept of goal-oriented care a step forward by stating that care "must above all consider patients' preferred outcomes" [8]. The focus on setting goals based on the patients' needs and preferences rather than on health-related outcomes became one of the main novelties in chronic disease management [6]. ***Not only could goal-oriented care be proposed as an important paradigm to overcome some of the new challenges for chronical patients [2], it might also corresponded to the original concept of evidence based medicine (EBM) [9]. EBM was first published by Sackett in 1996 who described three key components: 1. best external evidence, 2. individual clinical expertise, and 3. patients' values and expectations [9]. Since the first description of EBM, multiple approaches and paradigms has been developed to compromise between those three components [10]. For example, patient-centered care (PCC), which is already a well-known and widely used concept, is defined as "providing care that is***

			respectful of, and responsive to individual patient preferences, needs, and values and ensuring that patients values guides all clinical decisions" [10]. Shared-decision making, on the other hand, also strives to share evidence and engage patients in care as it is "an approach where clinicians and patients share the best available evidence when faced with the task of making decisions, and where patients are supported to consider options, and to achieve informed preferences" [11]. Goal-oriented care is proposed as a promising healthcare paradigm and approach to operationalize EBM and return to where it all started [8]. However, in contrast to the other approaches and paradigms, goal-oriented care is ill defined. Developing a common understanding on the concept could potentially contribute to the clarification and in-depth comparison between the related concepts and eventually lead to better use in clinical practice. However, some healthcare providers might already assume that they practice goal-oriented care spontaneously, but there still is a lack of underpinning knowledge and guidance on how to provide goal-oriented care to patients [4, 5]. As a first step in exploring the potential of goal-oriented care in chronic care, it is important to gain in-depth knowledge on what goal-oriented care is about and how it can be generally described.<br>As goal-oriented care could be well-suited in primary care, as this context is often the linchpin for patients with chronic conditions, this will be

			the focus of this study [12]. This study aimed to describe a structured approach to deepen the concept of goal-oriented care for patients with chronic conditions or multimorbidity in the primary care context.<br><br>(702 – strengths, limitations, and recommendations) This workflow should include the skills and tools so patients and providers can find a common ground in the goals and are supported in implementing goal-oriented care in practice. ***Then, when goal-oriented care is a well understood concept it is possible to perform an in-depth comparison between related concepts (e.g. patient-centered care).***
30	Please clarify how to "analyze" the included articles to define attributes in step 4. This can be helpful to understand the process of emerging codes for the readers who are not familiar with the concept analysis.	More clarification on the analyzing process is written down and an extra Table with an example of data extraction is given as illustration. This will provide more insight into the analyses.	***(L219 – method: step 4) Similar to a qualitative, thematic analysis, the key articles were analyzed based on an open coding and then grouped into codes. (Table 3 – example of data analysis). These codes were then presented to and discussed with the co-authors. Based on this discussion codes were translated into attributes.***

	Comments from reviewer 3	Response	Changes in the manuscript
Method			
31	Regarding the methods I assume there is a good reason why related concepts including patient-centered care, shared decision making and value based medicine were not included in the search string, but could this be argued more clearly?	Indeed, these concepts were not included in the literature search. The method of a concept analysis prescribed to focus on deepening just one concept. This is made clearer in the manuscript.<br>However, since reviewer 1 (9,10) and 2 (28) also comment on the absence of elaborating on relating concepts (more or less) we have added more information on that in the introduction and discussion.	***(L181 – method: select the literature) Related concepts such as patient-centered care, value-based care, etc. were not included as the method of concept analysis prescribes to deepen all the attributes of one concept.***<br><br>(L207 – method: step 3) Exclusion criteria: (a) focusing on single-disease management, (b) goals regarding disease-specific outcomes (e.g. cancer or diabetes), (c) focusing on goal-oriented care in a specific context (e.g. rehabilitation center), ***and (d) specifically mentioning patient-centered care, shared-decision making, etc. as they will hamper the understanding of specifically goal-oriented care.***<br><br>(L702 – discussion) This workflow should include the skills and tools so patients and providers can find a common ground in the goals and are supported in implementing goal-oriented care in practice. ***Then, when goal-oriented care is a well understood concept it is possible to perform an in-depth comparison between related concepts (e.g. patient-centered care).***

Results			
32	On page 18: what is the confusion in the paragraph above exactly? Different aspects are mentioned, but what is the tension?	This was indeed unclear, to clarify more on this tension, the sentences have been rewritten.	***(L357 – results: patients' needs and preferences) Other authors recommended that goals should be a combination of both the patients' goals and the providers' goals which in turn is related to goal-negotiation [16, 20]. In conclusion, no overall understanding on the goals could be formulated.***<br>***Besides this lack in understanding, there also seems to be ambiguity about the categorization of goals.*** Some authors emphasized that goals should contain core values of patients (e.g. the broader aspects that matter most to the patient) [6, 17] .
33	Regarding the results: Isn't there literature that defines goal oriented by stating what it is 'not'?	It is an interesting remark, but for this study we had to start from scratch to deepen the understanding of goal-oriented care. By using the method of a concept analysis we searched for what goal-oriented care 'is'. The approach of learning about a concept trough what it is not is in the method of a concept analysis defined as contrary case. From this perspective, we have given information about what it is not, unfortunately we did not found any information in the literature about what it is not and we have therefore made no changes in the manuscript.	No changes have been made in the manuscript.
34	Cases: The cases are not fully readable in the version of the paper that I had access to, but I think you need to work a bit on them to make them more believable or better: find actual cases. E.g. case 1: for most CVD / DM patients cycling is actually recommended and case 3:	Our excuses for the missing parts in the cases. Some changes have been made to be more realistic. We hope that this suits more now.	Joseph, 68- year old suffers from diabetes, hypertension and ***chronic obstructive pulmonary disease.***<br>Mary is a 40-year old mother of two young children and dealing with obesity since her childhood. ***Due to her weight, she has a lot of***

	obesity is hardly a barrier to travel. In reality I guess that most often patient's goals are not dismissed but are simply not discussed (for many reasons).		***joints pain and is short of breath which limits her exercising capacity. Her children would love nothing more than their mother play with them. Unfortunately, she is not able to play soccer or jump on the trampoline because of the pain. The pain becomes too much for her and after long hesitation she discusses this with her physician. The only thing she wants is to play and interact with her children as painless as possible and therefore asks her physician to prescribe some medication. Her physician does not support medication, but instructs her to first strive for a healthy weight as a solution to relieve the pain. This is not aligned with the wishes of Mary who only wanted a short-term solution to be able to play with her children.*** In the end, she leaves the consultation room with a referral to a dietitian and sport coach.
35	Page 24: was there nothing about systems preparedness? If so, please that this was not found.	It is an interesting remark to also include system preparedness, but the current literature did not point to that. We have reread the articles and went back to the raw data, but no information could be found on system preparedness. We have described this shortcoming in the discussion.	**(L635 – discussion)** ***Besides patient and provider preparedness, it could seem logical that also the system has to be prepared, but the current literature do not point to that.***
Discussion			
36	In the discussion I was expecting a juxtaposition of the goal oriented concept with related concepts in primary care, including generalism, holism, patient-centeredness, value based healthcare, shared decision making, patient participation, EBM (Sacket!) etc. How is it different? You could be more critical: is goal	This is an important remark as we did not elaborate on the related concepts. We also considered ourselves to juxtapose goal-oriented care to related concepts, but the method of concept analysis does not allow that.	(L95 – introduction) The concept of goal-oriented care has been launched and mentioned for the first time in 1991 by Mold who proposed the concept as an alternative way of providing care [7]. Later on, in 2012, Reuben and Tinetti took the concept of goal-oriented care a step forward by stating that care "must above all

	oriented care actually a better concept and if so why?	Though, we value your comment and especially concerning EBM and Sacket is a very interesting point, thank you for this. We have been thinking about it and we have added more information about how goal-oriented care link with EBM in the introduction.	consider patients' preferred outcomes" [8]. The focus on setting goals based on the patients' needs and preferences rather than on health-related outcomes became one of the main novelties in chronic disease management [6]. ***Not only could goal-oriented care be proposed as an important paradigm to overcome some of the new challenges for chronical patients [2], it might also corresponded to the original concept of evidence based medicine (EBM) [9]. EBM was first published by Sackett in 1996 who described three key components: 1. best external evidence, 2. individual clinical expertise, and 3. patients' values and expectations [9]. Since the first description of EBM, multiple approaches and paradigms has been developed to compromise between those three components [10]. For example, patient-centered care (PCC), which is already a well-known and widely used concept, is defined as "providing care that is respectful of, and responsive to individual patient preferences, needs, and values and ensuring that patients values guides all clinical decisions" [10]. Shared-decision making, on the other hand, also strives to share evidence and engage patients in care as it is "an approach where clinicians and patients share the best available evidence when faced with the task of making decisions, and where patients are supported to consider options, and to achieve informed preferences" [11]. Goal-oriented care is proposed as a promising healthcare paradigm and approach to operationalize EBM and return***

			to where it all started [8]. However, in contrast to the other approaches and paradigms, goal-oriented care is ill defined. Developing a common understanding on the concept could potentially contribute to the clarification and in-depth comparison between the related concepts and eventually lead to better use in clinical practice. However, some healthcare providers might already assume that they practice goal-oriented care spontaneously, but there still is a lack of underpinning knowledge and guidance on how to provide goal-oriented care to patients [4, 5]. As a first step in exploring the potential of goal-oriented care in chronic care, it is important to gain in-depth knowledge on what goal-oriented care is about and how it can be generally described.<br>As goal-oriented care could be well-suited in primary care, as this context is often the linchpin for patients with chronic conditions, this will be the focus of this study [12]. This study aimed to describe a structured approach to deepen the concept of goal-oriented care for patients with chronic conditions or multimorbidity in the primary care context.<br><br>(702 – strengths, limitations, and recommendations) This workflow should include the skills and tools so patients and providers can find a common ground in the goals and are supported in implementing goal-oriented care in practice. ***Then, when goal-oriented care is a well understood concept it is possible to perform an***

			in-depth comparison between related concepts (e.g. patient-centered care).
37	Perhaps also provide a discussion on tensions with contrasting (1) frameworks and (3) systems such as transnationalism, (4) care standards, P4P, neoliberal economics and budgeting (tension of providing efficient care for many).	It is a very interesting take to discuss (1) the tension with contrasting frameworks, but in this study we solely focused on an objective description of the information that was provided in the current literature. Little more attention on the link with the (2) care standards is provided in the introduction and discussion.<br><br>The absence of this juxtaposition with other frameworks is added in the discussion section as recommendation for further research.<br>To discuss a bit more on (3) contrasting systems, a cross-comparison study has been published after submitting this study. In this study, three international cases has adopted goal-oriented care and were linked it to integrated care. They found that goal-oriented could enable integrated care. It can be suggested that goal-oriented care has the potential to transit over specific health system and integrate them to each other [21].<br><br>We also value your comment to elaborate more on the (4) economic aspects of goal-oriented care. However, to investigate potential economic effects for goal-oriented care, further research is necessary. At this moment there is a discussion in the field about how to evaluate goal-oriented care. We have addressed this in the discussion section.	***(L181 – method: select the literature) Related concepts such as patient-centered care, value-based care, etc. were not included as the method of concept analysis prescribes to deepen all the attributes of one concept.***<br><br>(L207 – method: step 3) Exclusion criteria: (a) focusing on single-disease management, (b) goals regarding disease-specific outcomes (e.g. cancer or diabetes), (c) focusing on goal-oriented care in a specific context (e.g. rehabilitation center), ***and (d) specifically mentioning patient-centered care, shared-decision making, etc. as they will hamper the understanding of specifically goal-oriented care.***<br><br>(L95 – introduction) The concept of goal-oriented care has been launched and mentioned for the first time in 1991 by Mold who proposed the concept as an alternative way of providing care [7]. Later on, in 2012, Reuben and Tinetti took the concept of goal-oriented care a step forward by stating that care “must above all consider patients’ preferred outcomes” [8]. The focus on setting goals based on the patients’ needs and preferences rather than on health-related outcomes became one of the main novelties in chronic disease management [6]. ***Not only could goal-oriented care be proposed as an important paradigm to overcome some of***

			the new challenges for chronical patients [2], it might also corresponded to the original concept of evidence based medicine (EBM) [9]. EBM was first published by Sackett in 1996 who described three key components: 1. best external evidence, 2. individual clinical expertise, and 3. patients' values and expectations [9]. Since the first description of EBM, multiple approaches and paradigms has been developed to compromise between those three components [10]. For example, patient-centered care (PCC), which is already a well-known and widely used concept, is defined as "providing care that is respectful of, and responsive to individual patient preferences, needs, and values and ensuring that patients values guides all clinical decisions" [10]. Shared-decision making, on the other hand, also strives to share evidence and engage patients in care as it is "an approach where clinicians and patients share the best available evidence when faced with the task of making decisions, and where patients are supported to consider options, and to achieve informed preferences" [11]. Goal-oriented care is proposed as a promising healthcare paradigm and approach to operationalize EBM and return to where it all started [8]. However, in contrast to the other approaches and paradigms, goal-oriented care is ill defined. Developing a common understanding on the concept could potentially contribute to the clarification and in-depth comparison between the related concepts and eventually lead to better use in

			clinical practice. However, some healthcare providers might already assume that they practice goal-oriented care spontaneously, but there still is a lack of underpinning knowledge and guidance on how to provide goal-oriented care to patients [4, 5]. As a first step in exploring the potential of goal-oriented care in chronic care, it is important to gain in-depth knowledge on what goal-oriented care is about and how it can be generally described.<br>As goal-oriented care could be well-suited in primary care, as this context is often the linchpin for patients with chronic conditions, this will be the focus of this study [12]. This study aimed to describe a structured approach to deepen the concept of goal-oriented care for patients with chronic conditions or multimorbidity in the primary care context.<br><br>(L702 – discussion) This workflow should include the skills and tools so patients and providers can find a common ground in the goals and are supported in implementing goal-oriented care in practice. ***Then, when goal-oriented care is a well understood concept it is possible to perform an in-depth comparison between related concepts (e.g. patient-centered care).***
38	I would also like to see what was missing in the literature / what was not mentioned? E.g. as I mentioned above: system preparedness (as opposed to patient and provider preparedness).	Indeed, their remain some knowledge gaps after analyzing the current literature. The literature learned us that goal-oriented is a stepwise approach, but it is still unclear how this theory should be translated into a practical	***(L 635 – discussion) Besides patient and provider preparedness, it could seem logical that also the system has to be prepared, but the current literature do not point to that.***

	Anything else?	approach including the organization of patient preparedness and interprofessional collaboration. Besides the lack of a practical workflow, there is also a lack on how to develop process indicators to eventually evaluate a goal-oriented care practice. Also, to meet the previous comment of juxtaposing goal-oriented care with other frameworks and systems, more research will be needed as the current literature could not provide this answer. To conclude that this concept analysis is first main step to facilitate further research on divers topics related to goal-oriented care.	***(L683 – strengths, limitations, and recommendations) One of the knowledge gaps revealed in this concept analysis is the lack of knowledge on what patients' goals are set, how goal-oriented care is delivered, and how it is best put into practice in both one-on-one interactions between patients and providers and in interprofessional collaboration.*** Regarding patients it is important ***to gain more insight in how they are preferably prepared for discussing their personal goals.***<br><br>***(L700 – strengths, limitations, and recommendations) However, further research is needed to translate the current knowledge on the concept of goal-oriented care into a tangible workflow process of care that entails the three stages.***

Comments of reviewer 4		Response	Changes in the manuscript
Results			
39	This case (Mary) feels a bit tenuous - it feels unlikely a family doctor would not 'allow' travel plans on reasonable grounds. I understand what you are trying to illustrate - but a better example is needed.	Thank you for this remark. Also reviewer 1, 2, and 3 (24,32) made this comment. A more realistic example (checked with GP) is described.	Mary is a 40-year old mother of two young children and dealing with obesity since her childhood. ***Due to her weight, she has a lot of joints pain and is short of breath which limits her exercising capacity. Her children would love nothing more than their mother play with them. Unfortunately, she is not able to play soccer or jump on the trampoline because of the pain. The pain becomes too much for her and after long hesitation she discusses this with her physician. The only thing she wants is to play and interact with her children as painless as possible and therefore asks her physician to prescribe some medication. Her physician does not support medication, but instructs her to first strive for a healthy weight as a solution to relieve the pain. This is not aligned with the wishes of Mary who only wanted a short-term solution to be able to play with her children.*** In the end, she leaves the consultation room with a referral to a dietitian and sport coach.
40	(L220) This is a really important point, and the likely reason for resistance by providers.<br>to goal-oriented care. It feels like it would be of value to expand on this further if possible. Did the literature explore this in further depth - what role does the provider have in shifting patients' goals and how should they do this, what are the ethics behind trying to do so, how is this best done in a way that does not alienate	We are aware that we have not further discussed this point. It is indeed really important to explore this more in depth, because we believe that we first have to answer these questions before we can fully implement goal-oriented care in practice. The current literature could not provide us an answer on this. This comment is also about how we can translate the patients' (life) goals to the providers' (medical) goals and eventually	(L699 – strengths, limitations, and recommendations) Goal-oriented care shows the potential to be a way forward for patients with chronic conditions and multimorbidity. However, further research is needed to translate the current knowledge on the concept of goal-oriented care into a tangible workflow process of care that entails the three stages. ***This workflow should include the skills and tools so patients and providers can find a common***

	the patient?	develop a workflow for goal-oriented care. Unfortunately, this concept analysis could not provide us with the needed information to answer these questions and provided an objective description of what we found in literature.<br><br>We thank you for this comment, you really touched very important points regarding goal-oriented care and these topics will be subject to further research. We have added this ideas in the discussion section as recommendations for further research.	***ground in the goals and are supported in implementing goal-oriented care in practice.***
41	The term 'empirical referent' is hard to understand, although I can see you have described it - in order to bring it to life I wonder if the table with the examples could provide more details.	The term empirical referent is indeed complex to understand, but we have chosen to stick to the prescribed steps of Walker and Avant and their naming. To clarify this a bit more, the purpose of each tool or measurement and for who it can be used has been added in the overview.	Adjustments have been made to Table 4 with more details about the purpose of each tool, measurement.
Discussion			
42	I wondered if a bit more discussion is needed about the risks of when patient and providers goals do not align, and approaches needed to align these.	It is interesting to deepen potential risks in case that patient and provider goals do not align. At this moment, the literature does not address this risk and could therefore be further explored. What we do know is that, in case that those goals do not align, it could cause conflicts between patients and providers. We described those potential conflicts and strategies to overcome them in the discussion part.	***(L591 – discussion) Not only is there ambiguity on what goals patients set, it is also not clear how goals are being set. What is clear is that patients and providers must collaborate and negotiate on which goals are important. Nevertheless, this can still cause conflicts between the patients' goals and providers' goals [22, 23]. To overcome these conflicts, it is suggested to first set the patients' goals and then discuss about the medical goals, because conflicts are more likely when goals are placed on the same level [24]. It should however be***

			noted that setting the patients' goals on top does not legitimate full patients' responsibility over the care plan [24]. Another way to overcome these conflicts is to work with a facilitator as Naik et al. did in developing their patients priorities identification process. These facilitators supported patients in setting goals, choosing the most important goals to eventually communicate them with the provider [6]. Yet another strategy is to use tools to assess patient treatment priorities and preferences. Unfortunately, Mangin et al. found few relevant tools to set patients' goals [19]. They argue for the need to develop specific strategies to make patient priorities visible in the clinical record and medical-decision making [19].
43	(L253) What if any, guidance is there for providers to work out how to align patient needs and preference with those of their health and the health system.	It is an interesting question as this will be one of the main challenges to eventually implement goal-oriented care in practice. In literature that is not clearly described, but some strategies have been proposed. These strategies have been added in the discussion (cfr. answer previous comment)	***(L591 – discussion) Not only is there ambiguity on what goals patients set, it is also not clear how goals are being set. What is clear is that patients and providers must collaborate and negotiate on which goals are important. Nevertheless, this can still cause conflicts between the patients' goals and providers' goals [22, 23]. To overcome these conflicts, it is suggested to first set the patients' goals and then discuss about the medical goals, because conflicts are more likely when goals are placed on the same level [24]. It should however be noted that setting the patients' goals on top does not legitimate full patients' responsibility over the care plan [24]. Another way to overcome these conflicts is to work with a facilitator as Naik et al. did in developing their***

			patients priorities identification process. These facilitators supported patients in setting goals, choosing the most important goals to eventually communicate them with the provider [6]. Yet another strategy is to use tools to assess patient treatment priorities and preferences. Unfortunately, Mangin et al. found few relevant tools to set patients' goals [19]. They argue for the need to develop specific strategies to make patient priorities visible in the clinical record and medical-decision making [19].<br><br>Goal-oriented care shows the potential to be a way forward for patients with chronic conditions and multimorbidity. However, further research is needed to translate the current knowledge on the concept of goal-oriented care into a tangible workflow process of care that entails the three stages. ***This workflow should include the skills and tools so patients and providers can find a common ground in the goals and are supported in implementing goal-oriented care in practice.***
44	(L323) This sentence is a broad generalization. Could it be that sometime or often this happens... not always.	The sentence is written more 'carefully'.	Currently patients ***are not always stimulated*** to think about their care.
45	(L327) This is a broad generalization, I think there are GPs who are aware of these. Again could the word often or sometimes be used to moderate the sentence?	As the previous comment, the word 'often' is added.	Voigt et al. observed that GPs ***are often unaware*** of patients' priorities in daily life, which were in contrast with their perceived importance of patient's medical goals
46	(337) This sentence seems to contradict the next one (limited studies, and then is says mostly positive outcomes).	The positive outcomes were related to the results of the founded studies. This has been made more clear.	(L638 – discussion) In terms of the consequences of goal-oriented care, ***a limited number of studies have been able to demonstrate outcomes of goal-oriented care.***

			Nonetheless, these studies showed mostly positive outcomes towards the patients, providers, health system, and overall population well-being.
47	Supporting files: These supplementary documents are helpful and interesting- can you make reference to them in the main text so the reader knows when to refer to them.	We have checked the references to the supporting files and added if we needed to.	(L262 – results) goal-oriented care ***(S1 Table 1)*** (L 439 – antecedents) in each training ***(S2 Table 2).***

1. Levac D, Colquhoun H, O'Brien KK. Scoping studies: advancing the methodology. Implement Sci. 2010;5:69. Epub 2010/09/22. doi: 10.1186/1748-5908-5-69. PubMed PMID: 20854677; PubMed Central PMCID: PMCPMC2954944.
2. Kuluski K, Peckham A, Gill A, Gagnon D, Wong-Cornall C, McKillop A, et al. What is Important to Older People with Multimorbidity and Their Caregivers? Identifying Attributes of Person Centered Care from the User Perspective. Int J Integr Care. 2019;19(3):4. Epub 2019/08/02. doi: 10.5334/ijic.4655. PubMed PMID: 31367203; PubMed Central PMCID: PMCPMC6659759.
3. Kuipers SJ, Cramm JM, Nieboer AP. The importance of patient-centered care and co-creation of care for satisfaction with care and physical and social well-being of patients with multi-morbidity in the primary care setting. BMC Health Serv Res. 2019;19(1):1-9.
4. Vermunt N, Harmsen M, Westert GP, Olde Rikkert MGM, Faber MJ. Collaborative goal setting with elderly patients with chronic disease or multimorbidity: a systematic review. BMC Geriatr. 2017;17(1):167. Epub 2017/08/02. doi: 10.1186/s12877-017-0534-0. PubMed PMID: 28760149; PubMed Central PMCID: PMCPMC5537926.
5. Poitras ME, Maltais ME, Bestard-Denomme L, Stewart M, Fortin M. What are the effective elements in patient-centered and multimorbidity care? A scoping review. BMC Health Serv Res. 2018;18(1):446. Epub 2018/06/15. doi: 10.1186/s12913-018-3213-8. PubMed PMID: 29898713; PubMed Central PMCID: PMCPMC6001147.
6. Naik AD, Dindo LN, Van Liew JR, Hundt NE, Vo L, Hern, et al. Development of a Clinically Feasible Process for Identifying Individual Health Priorities. Journal of the American Geriatrics Society. 2018;66(10):1872-9. PubMed PMID: rayyan-47069467.
7. Mold JW, Blake GH, Becker LA. Goal-oriented medical care. Family medicine. 1991;23(1):46-51. Epub 1991/01/01. PubMed PMID: 2001782.
8. Reuben DB, Tinetti ME. Goal-oriented patient care--an alternative health outcomes paradigm. The New England journal of medicine. 2012;366(9):777-9. Epub 2012/03/02. doi: 10.1056/NEJMp1113631. PubMed PMID: 22375966.
9. Sackett DL, Rosenberg WM, Gray JM, Haynes RB, Richardson WS. Evidence based medicine: what it is and what it isn't. British Medical Journal Publishing Group; 1996.
10. Baker A. Crossing the quality chasm: a new health system for the 21st century: British Medical Journal Publishing Group; 2001.
11. Elwyn G, Frosch D, Thomson R, Joseph-Williams N, Lloyd A, Kinnersley P, et al. Shared decision making: a model for clinical practice. Journal of general internal medicine. 2012;27(10):1361-7.

12. Hobbs FR, Bankhead C, Mukhtar T, Stevens S, Perera-Salazar R, Holt T, et al. Clinical workload in UK primary care: a retrospective analysis of 100 million consultations in England, 2007–14. The Lancet. 2016;387(10035):2323-30.
13. Mold. Goal-directed health care: redefining health and health care in the era of value-based care. Cureus 2017;9(2).
14. Berntsen G, Hoyem A, Lettrem I, Ruland C, Rumpsfeld M, Gammon D. A person-centered integrated care quality framework, based on a qualitative study of patients' evaluation of care in light of chronic care ideals. BMC Health Serv Res. 2018;18(1):479. Epub 2018/06/22. doi: 10.1186/s12913-018-3246-z. PubMed PMID: 29925357; PubMed Central PMCID: PMCPMC6011266.
15. Salter C, Shiner A, Lenaghan E, Murdoch J, Ford JA, Winterburn S, et al. Setting goals with patients living with multimorbidity: qualitative analysis of general practice consultations. Br J Gen Pract. 2019;69(684):e479-e88. Epub 2019/06/05. doi: 10.3399/bjgp19X704129. PubMed PMID: 31160370; PubMed Central PMCID: PMCPMC6592350.
16. Kessler D, Walker I, Sauve-Schenk K, Egan M. Goal setting dynamics that facilitate or impede a client-centered approach. Scandinavian journal of occupational therapy. 2018;26(5):315-24. PubMed PMID: 29671662.
17. Tinetti ME, Esterson J, Ferris R, Posner P, Blaum CS. Patient Priority-Directed Decision Making and Care for Older Adults with Multiple Chronic Conditions. Clinics in geriatric medicine. 2016;32(2):261-75. PubMed PMID: 27113145.
18. Murdoch J, Salter C, Ford J, Lenaghan E, Shiner A, Steel N. The "unknown territory" of goal-setting: Negotiating a novel interactional activity within primary care doctor-patient consultations for patients with multiple chronic conditions. Soc Sci Med. 2020;256:113040. Epub 2020/05/31. doi: 10.1016/j.socscimed.2020.113040. PubMed PMID: 32473530; PubMed Central PMCID: PMCPMC7306159.
19. Mangin D, Stephen G, Bismah V, Risdon C. Making patient values visible in healthcare: a systematic review of tools to assess patient treatment priorities and preferences in the context of multimorbidity. BMJ open. 2016;6(6):e010903.
20. Kuluski K, Guilcher SJT. Toward a Person-Centred Learning Health System: Understanding Value from the Perspectives of Patients and Caregivers. HealthcarePapers. 2019;18(4):36-46. Epub 2020/01/05. doi: 10.12927/hcpap.2019.26030. PubMed PMID: 31901067.
21. Steele Gray C, Grudniewicz A, Armas A, Mold J, Im J, Boeckxstaens P. Goal-Oriented Care: A Catalyst for Person-Centred System Integration. Int J Integr Care. 2020;20(4):8. Epub 2020/11/18. doi: 10.5334/ijic.5520. PubMed PMID: 33199976; PubMed Central PMCID: PMCPMC7646288.
22. Bayliss EA, Bonds DE, Boyd CM, Davis MM, Finke B, Fox MH, et al. Understanding the context of health for persons with multiple chronic conditions: moving from what is the matter to what matters. Annals of family medicine. 2014;12(3):260-9. Epub 2014/05/14. doi: 10.1370/afm.1643. PubMed PMID: 24821898; PubMed Central PMCID: PMCPMC4018375.
23. Fried TR, Tinetti M, Agostini J, Iannone L, Towle V. Health outcome prioritization to elicit preferences of older persons with multiple health conditions. Patient education and counseling. 2011;83(2):278-82.
24. Berntsen GK, Gammon D, Steinsbekk A, Salamonsen A, Foss N, Ruland C, et al. How do we deal with multiple goals for care within an individual patient trajectory? A document content analysis of health service research papers on goals for care. BMJ open. 2015;5(12):e009403. Epub 2015/12/15. doi: 10.1136/bmjopen-2015-009403. PubMed PMID: 26656243; PubMed Central PMCID: PMCPMC4679896.
