# Supplementary material for: Goal-oriented care for patients with chronic conditions or multimorbidity in primary care: A scoping review and concept analysis
Source: PLoS One. 2022 Feb 4;17(2):e0262843. doi: 10.1371/journal.pone.0262843 (PMC8815876; doi:10.1371/journal.pone.0262843)
Supplement: Attachment — Submitted filename: Response to the reviewers.docx [file pone.0262843.s005.docx]

Dear Editor
Dear reviewers

We appreciate your extensive and constructive feedback on our manuscript.
We aimed to respond all of your remarks by point to point answers and changes in the manuscript.
Hopefully they will meet your expectations, otherwise we will make further adjustments.

Sincerely
Dagje Boeykens, on behalf of the co-authors

|  | **Comments from the editor** | **Response** | **Changes in the manuscript** |
| --- | --- | --- | --- |
| 1 | Please ensure that your manuscript meets PLOS ONE's style requirements, including those for file naming. | Adjustments have been made to meet the PLOS ONE’s style requirements. | Changes in the titles and references to figures and tables. |
| 2 | During the internal evaluation of the manuscript, we feel that this study fits within the scope of a Scoping Review. As such we please consider modifying your title to specify this. For instance "Goal-oriented care in primary care: a scoping review and concept analysis. | Thank you for this comment and we do agree with the fact that no specifications are given regarding the way how the literature search should be performed in a concept analysis according to Walker and Avant. From this perspective we can agree with the comment to think about adding ‘scoping review’ to the title because there are indeed some similarities. However, a scoping review and a concept-analysis are two different methodologies and we did not follow the guidelines from a scoping review (e.g. guidelines Joan Briggs institute or Arksey and O’Malley), but the guidelines from Walker and Avant. So we are a little bit puzzled by this request, in the one hand we would like to meet your comment, but on the other hand we feel that we cannot change the title and method because we did not perform a scoping review as it should have been done. From this perspective, we would like to suggest no changes to the title (but perhaps add it as a key word).  However, if this comment is a breaking point for being accepted as publication, and from the aspect to be identifiable by possible readers as a review article we can perfectly follow this comment. The way we have performed our literature search is similar to the way Levac describes how a literature study should be performed in scoping reviews. So, from this perspective we can follow to add ‘scoping review’ to the title, but then we suggest to add a few words to the method section and to the discussion section. In that case, please let us know.  Levac D, Colquhoun H, O'Brien KK. Scoping studies: advancing the methodology. Implement Sci. 2010;5:69. | Our primary option would be not to change the title and keep the original title.  In case we should change, we would suggest the following title,  Goal-oriented care ***for patients with chronic conditions or multimorbidity: a scoping review*** and concept analysis  In the method section we added the following lines:  ***(L173 – method: step3) As the method of a concept analysis does not specify how the literature search has to be performed, this search was based on the method of a scoping review described by Levac (2010)*** [1]  ***Related concepts such as patient-centered care, value-based care, etc. were not included as the method of concept analysis prescribes to deepen all the attributes of one concept.***  And add in the discussion section the following lines in the limitation section:  ***(L666 – limitations) Since there is no specification given by Walker and Avant on how to conduct the literature review, we followed the guideline from a scoping review as described by Levac (2010). This could have influenced the results.*** |
| 3 | In addition please provide a PRIMSA flow chart as Figure 1, list of studies as Table 1, and a completed PRISMA-Scr checklist as Supporting File | A PRISMA-Scr has been completed and also added to the supporting files. For us it was not that clear if the flow chart and tables have to be replaced to the supporting files, so if we need to do that, please let us know. | A completed Prisma-Scr checklist has been added and presented at the supporting files. |
| 4 | Finally, please include in your Methods section the date ranges over which you conducted the literature search. | The date range has been added in the method section. | ***(L 173 – select the literature): The literature search was conducted between January 2020 and April 2020***. A preliminary combination of… |
| 5 | One of the noted authors is a group or consortium Primary Care Academy. In addition to naming the author group, please list the individual authors and affiliations within this group in the acknowledgments section of your manuscript. Please also indicate clearly a lead author for this group along with a contact email address. | A list of the individual authors and affiliations has been made and added to the acknowledgments. Prof. dr. Roy Remmen ([roy.remmen@uantwerpen.be](mailto:roy.remmen@uantwerpen.be)) has been indicated as the lead author of the consortium. | / |
| 6 | Please review your reference list to ensure that it is complete and correct. If you have cited papers that have been retracted, please include the rationale for doing so in the manuscript text, or remove these references and replace them with relevant current references. If you need to cite a retracted article, indicate the article’s retracted status in the References list and also include a citation and full reference for the retraction notice. | The references list is checked and adjustments have been made. | 21. Institute of Medicine Committee on the Future of Primary C. In: Donaldson M, Yordy K, Vanselow N, editors. Defining Primary Care: An Interim Report. Washington (DC): National Academies Press (US)  Copyright 1994 by the National Academy of Sciences.; 1994.  36. Purkaple BA, Nagykaldi ZJ, Allahyar A, Todd R, Mold JW. Physicians' Response to Patients' Quality-of-Life Goals. J Am Board Fam Med. 2020;33(1):71-9. |

| **Comments from reviewer 1** | | **Response** | **Changes in the manuscript** |
| --- | --- | --- | --- |
| Title | |  |  |
| 7 | No mention is made of multimorbidity, a key concept within the paper. It should be added in. | Indeed, the concept analysis also focusses on chronic conditions/ multimorbidity, so this is added to the title. | Goal-oriented care **for patients with chronic conditions and multimorbidity** in primary care: a concept analysis. |
| Introduction | |  |  |
| 8 | An assumption is made throughout that goal oriented care is likely to be better - can the authors provide any effectiveness data related to this topic? There are a number of trials in multimorbidity in which a goal setting approach is used. Likewise in the abstract intro. | Goal-oriented care has indeed some potential benefits. As the outcomes of the effectiveness studies were considered as results of the analyzing process under ‘consequences’ of the result sections they were not specifically mentioned in the introduction. The outcomes of effectiveness studies have also been discussed in the ‘discussion section’ under the paragraph of ‘consequences’. To meet the comment, we have given more attention to the potential outcomes of meeting the patients’ needs in the introduction. Though, it should be noticed that more research is needed to elaborate on the effectiveness data on this topic. | (L86 – introduction) A possible way to overcome many of the challenges is to shift care back from ‘what’s the matter with the patient’ to ‘what matters to the patient’. It creates health care processes in which patients’ needs are actively sought and met [2]. ***Meeting those patients’ needs and tailoring care more to what patients want in a co-creation process is assumed to result in better social well-being, physical well-being, and satisfaction for patients and healthcare providers [3].*** |
| 9 | Disease-specific care is positioned as opposite to goal oriented care. However, within a number of chronic disease management programs, goal setting plays a large part (although the patient centeredness of this may be debatable). Perhaps the authors need to more clearly distinguish between goal setting in care and goal-oriented care, which seems to be broader in their analysis. | Indeed, there is a need to distinguish between goal setting and goal-oriented care and we recognize that goal-setting plays a large part in the entire organization of (chronic) care.  This comment has been addressed in the introduction and in the discussion section. | ***(L114 – introduction) Some healthcare providers might already assume that they practice goal-oriented care spontaneously, but there still is a lack of underpinning knowledge and guidance on how to provide goal-oriented care to patients The main pitfall in most of these goal-setting activities is that the goals are not necessarily related to the patients’ needs and preferences while in goal-oriented care these patients’ needs and preferences are put on the forefront and not necessarily health-related*** ***[4, 5]. From this perspective, goal-setting and goal-oriented care should be taken together and focusing on the patient’s needs and preferences.***  ***(L580 – discussion) In goal-oriented care it seems important to set goals based on the patients’ needs and preferences (e.g; I want to take my grandchildren to school), while in other chronic disease management programs the emphasis is mainly still on health-related goals [6] (e.g. I want the patient to be able to walk without pain).*** |
| 10 | Likewise, other related concepts such as shared decision making and patient centered care are only briefly touched upon and in either the intro or discussion or both need to be discussed as to how they relate to goal oriented care. | This is an important remark as we did not elaborate on the related concepts. We consider this concept analysis as a first and main step in learning more about goal-oriented care. The method of a concept analysis did not allow us to make an overview of the differences and similarities of the related concepts.  Although, to meet this valuable comment we have added more information in the introduction. | (L93 – introduction) The concept of goal-oriented care has been launched and mentioned for the first time in 1991 by Mold who proposed the concept as an alternative way of providing care [7]. Later on, in 2012, Reuben and Tinetti took the concept of goal-oriented care a step forward by stating that care “must above all consider patients’ preferred outcomes” [8]. The focus on setting goals based on the patients’ needs and preferences rather than on health-related outcomes became one of the main novelties in chronic disease management [6]. ***Not only could goal-oriented care be proposed as an important paradigm to overcome some of the new challenges for chronical patients [2], it might also corresponded to the original concept of evidence based medicine (EBM) [9]. EBM was first published by Sackett in 1996 who described three key components: 1. best external evidence, 2. individual clinical expertise, and 3. patients’ values and expectations [9]. Since the first description of EBM, multiple approaches and paradigms has been developed to compromise between those three components [10]. For example, patient-centered care (PCC), which is already a well-known and widely used concept, is defined as “providing care that is respectful of, and responsive to individual patient preferences, needs, and values and ensuring that patients values guides all clinical decisions” [10]. Shared-decision making, on the other hand, also strives to share evidence and engage patients in care as it is “an approach where clinicians and patients share the best available evidence when faced with the task of making decisions, and where patients are supported to consider options, and to achieve informed preferences” [11]. Goal-oriented care is proposed as a promising healthcare paradigm and approach to operationalize EBM and return to where it all started [8]. However, in contrast to the other approaches and paradigms, goal-oriented care is ill defined.*** ***Developing a common understanding on the concept could potentially contribute to the clarification and in-depth comparison between the related concepts and eventually lead to better use in clinical practice. However,*** some healthcare providers might already assume that they practice goal-oriented care spontaneously, but there still is a lack of underpinning knowledge and guidance on how to provide goal-oriented care to patients [4, 5]. As a first step in exploring the potential of goal-oriented care in chronic care, it is important to gain in-depth knowledge on what goal-oriented care is about and how it can be generally described.  As goal-oriented care could be well-suited in primary care, as this context is often the linchpin for patients with chronic conditions, this will be the focus of this study [12]. This study aimed to describe a structured approach to deepen the concept of goal-oriented care for patients with chronic conditions or multimorbidity in the primary care context. |
| 11 | Primary care is seen as the main focus of this paper, which makes sense, but there is little detail on collaboration and how goal oriented care would fit with collaborative approaches and whose responsibility in the primary care team it could/should be. | This is an important remark that we questioned ourselves already several times. Overall, the current literature pays little attention on (interprofessional) collaboration and how goal-oriented care could potentially facilitate this collaboration. Only one study (Vermunt et al.) mentioned who should take the lead during the process, suggesting different professionals including to be GP, nurse, practice nurse, psychological wellbeing practitioner. This information is now more specified. In a study of Mold it was suggested that occupational therapists as probably the most ‘goal-oriented’ profession without specifying that this profession should take the responsibility [13] | ***(L397 – results: care plan is based on patients’ needs and preferences) To guide this interprofessional review, no specification was given about which profile would be the best fit for having the lead. Vermunt et al. (2017) illustrated this as they found variation in who (e.g. GP, nurse, practice nurse, psychological wellbeing practitioner) should contribute to goal-setting [4].*** |
| Method | |  |  |
| 12 | I appreciate the literature searches are iterative but it would be useful to know the range of dates searched or at the very least the date of the most recent search, in order to contextualize the point at which this was done. | The date range is added to step 3 of the method section. | ***(L173 – method: step 3) The literature search was conducted between January 2020 and April 2020***. A preliminary combination of… |
| 13 | 'confirmed the first results' unclear what this means - no new studies found? or no new concepts identified? | Indeed, this was unclear. We meant that no new studies were identified. Further details on this method part has been added. | (L183 – method: step3) In a second phase, CINAHL, OTSeeker, PsycINFO and Web of Science were consulted and confirmed the first results ***as no new studies were identified.*** |
| 14 | In the inclusion criteria, it is unclear how much of a focus was needed on goal oriented care to be included - was there a minimum level of discussion or characterization required? How many authors determined it had sufficient focus? To me this would seem to be difficult to judge. | It was indeed difficult to judge if a study should be included or not as goal-oriented care is a poorly defined concept (this was the reason why we conducted this concept analysis in the first place). However, by predefining the inclusion criteria we tried to make this choice more objective. Further, the studies were discussed with the co-authors to evaluate if they could contribute to the theoretical building of an understanding on goal-oriented care. Each co-author had an open and reflexive view to the articles so the risk of bias was reduced. We also strived for consensus with the authors in several discussion round that were held to increase reliability of the inclusion process.  We added this in the method section. | (L 217 – method: step 4) The determination of the attributes started with a discussion of four key articles [14-17] selected by the first author based on the divers approaches of goal-oriented care ***and presented to the research group.***  ***(L223 – method: step 4) In a second phase, new articles were added and analyzed based on the same method as the key articles until all relevant literature (based on the inclusion criteria) was included.*** |
| 15 | Clarify that all paper types were included. | A sentence is added to make this more clear. | ***(L214 – method: step 3) There was no restriction by study design to gain as much insight in goal-oriented from different perspectives.*** |
| 16 | "a chronic condition"? This paper is focused on multimorbidity so surely it should be >1? | The paper is focused on both, just one chronic condition or multiple conditions (multimorbidity). Changes have been made. | (L206 – method: step 3) and (c) focusing on patients with ***one or more chronic conditions.*** |
| 17 | Papers included were English only. It would be interesting to know how many papers were in another language as it seems like that there may be cultural differences that could not be picked up? | This will indeed be interesting to identify potential cultural differences. During the literature search, no specific filters were used. This allowed us to, indeed, have knowledge about papers in other languages and eventually potential cultural differences.  However, no studies in other languages could be found by means of our search strategy. Though, English written papers could also provide insight in differences relating to context and culture, but the included articles related to Western countries so at this moment no differences could be identified. We added this in the limitation section. | In the limitation section we have added the following:  ***(L674 – limitations) The literature that was included in this study were only English written and peer reviewed. It would however be interesting to add also non-English papers to be able to capture more differences (e.g. cultural differences)*** |
| Results | |  |  |
| 18 | Results: flow chart too low res to view. | A new flow chart has been made with better resolution. Following the editor’s suggestion this flowchart has now been placed in the supplementary files. |  |
| 19 | Table 2 is clear but would appreciate adding further details on methods, plus perhaps contribution it made to the concept analysis (could add in numbers from table 3 to indicate where contributed to?). Some study methods are blank? I don't see the relevance of journal. | Table 2 is supplemented with more information about the method.  Further, references are added in Table 3 so the link to the corresponding article is more clear. | Table 2 is completed and references are added in Table 3. |
| 20 | p18 patient's needs and preferences... within this section I wondered if any of the papers had picked up on expectation management, which would seem to be an important part of the process which is not really considered here. | It is an interesting remark to link the paragraph of the patients’ needs and preferences to expectation management. However, after rereading the included articles none of them relate to that. What we see in the literature that has been published after the literature search was finalized, is that it is important to find the underlying values of goals patients set [18]. We agree that this is an important issue, but based on the literature we have reviewed we cannot provide an answer and therefore we did not make any changes in the manuscript. | No changes have been made in the manuscript. |
| 21 | Additionally on page 19 I wondered what the implications were when people's goals went beyond the remit of healthcare professionals in terms of their aims or their barriers and facilitators (e.g. finances, caring responsibilities) and whether the responsibility should be on the HCP to address these kind of issues as part of holistic care or the patient as part of their own self-management? This could also be picked up on the discussion and implications. | Thank you for this comment. Some authors, however limited, touched upon this issue. We have addressed this comment in the discussion section. | ***(L394 – results: care plan is based on patients’ needs and preferences) In case that providers are confronted with patients’ goals that are out of their own scope, they could benefit from an interprofessional review as they are enabled to discuss with and hand over to others with the needed expertise. This could improve the coordination of the care plans between the different providers and facilitate integrated care delivery [6, 17, 19]. To guide this interprofessional review, no specification was given about which profile would be the best fit for having the lead. Vermunt et al. (2017) illustrated this as they found variation in who (e.g. GP, nurse, practice nurse, psychological wellbeing practitioner) should contribute to goal-setting [4].***  (L 631 – discussion) Therefore training should also include the interprofessional perspective to facilitate a uniform attitude towards the patients’ goals and principles of goal-oriented care in the entire team. ***This will potential support providers to learn from and with each other’s’ expertise and enable discussion between them in case that, for example, patients set goals that our out of the remit of the provider.*** |
| 22 | It is somewhat unclear who should be leading/doing the care plan. Whilst an interprofessional approach is emphasized, there is little detail on whether a specific role (e.g. care coordinator or similar) would be required for this process to take place. | We do agree with this comment and agree that interprofessional collaboration is an important topic to relate with goal-oriented care. Besides Vermunt (2010) little attention is given in the contemporary literature describing the concept of goal oriented care who should take the lead or responsibility in the process. We have added a few lines to address this issue. | ***(L394 – results: care plan is based on patients’ needs and preferences) In case that providers are confronted with patients’ goals that are out of their own scope, they could benefit from an interprofessional review as they are enabled to discuss with and hand over to other providers with the needed expertise. This could improve the coordination of the care plans between the different providers and facilitate integrated care delivery [6, 17, 19]. To guide this interprofessional review, no specification was given about which profile would be the best fit for having the lead. Vermunt et al. illustrated this as they found variation in who (e.g. GP, nurse, practice nurse, psychological wellbeing practitioner) should contribute to goal-setting [4].*** |
| 23 | There's also little differentiation throughout as to whether the goals set are meant to be actioned by the patient or providers, which is an important consideration when considering measurement and review and goal level. | This is an important remark as this will be one of the main concerns of providers and patients to apply goal-oriented care. However, in the current (included) literature no specification was given regarding the person who should action the goals. What we saw in the literature is that the provider should support patients in undertaking action to achieve their goals, but that they should let the patients in their own responsibility in whether or not striving for their goals. It is however important to notice that in goal-oriented care it is most of all important to identify the patients’ needs and preferences to guide the conversations and interaction rather than focusing on achieving the patients’ goals. | (L: 699 – strengths, limitations, and recommendations) Goal-oriented care shows the potential to be a way forward for patients with chronic conditions and multimorbidity. However, further research is needed to translate the current knowledge on the concept of goal-oriented care into a tangible workflow process of care that entails the three stages.  This workflow should consists of tools to prepare patients and providers to offer goal-oriented care. This could contribute to finding a common ground in the goals and implementing goal-oriented care in practice. |
| 24 | Throughout it would be good to know when a paper is quoted, the type of data/expertise it is coming from and whether these characterizations are consistent across methods (e.g. whether characterized in the same way from a detailed conversational analysis perspective vs an overview paper) | Thank you for this suggestion. It indeed is interesting to get an overview of the included articles and the method they have used. Therefore we have integrated the previous comment (18) to complete Table 2 and make the link with Table 3. Together with a descriptive overview of the diverse study types we tried to provide this overview. | ***(L257 – method: step 3) These articles represented a broad range of study types: 4 systematic reviews, 4 experimental studies (e.g. randomized controlled trial), 13 qualitative studies, 3 survey studies, 1 concept analysis, 1 methodology paper, 4 reviews, 2 position papers, 1 background paper, 1 status report, 1 commentary, 1 opinion paper, and 1 perspective.***  ***+ Table 2 and 3*** |
| 25 | The case boxes are cut off so I can't comment on them. Box 3 states 'her physician does not allow' - does this mean he is preventing Mary from travelling, which does not seem like something he would be able to, or is this perhaps a slight miswording? | Our excuses for the missing parts of the cases. Also reviewer 2,3, and 4 comment (32 & 37) that the case of Mary could seem slightly unrealistic. To meet these comments, the case has been rewritten. | Mary is a 40-year old mother of two young children and dealing with obesity since her childhood. ***Due to her weight, she has a lot of joints pain and is short of breath which limits her exercising capacity. Her children would love nothing more than their mother play with them. Unfortunately, she is not able to play soccer or jump on the trampoline because of the pain. The pain becomes too much for her and after long hesitation she discusses this with her physician. The only thing she wants is to play and interact with her children as painless as possible and therefore asks her physician to prescribe some medication. Her physician does not support medication, but instructs her to first strive for a healthy weight as a solution to relieve the pain. This is not aligned with the wishes of Mary who only wanted a short-term solution to be able to play with her children.*** In the end, she leaves the consultation room with a referral to a dietitian and sport coach. |
| 26 | In the patient preparedness part, could any conclusions be drawn about the best ways to prepare patients? | Thank you for this interesting remark, but unfortunately no in-depth information was given in the literature regarding the way how patients should be prepared. It is something we also questioned ourselves. We added specifically in the discussion section that this will be subject to further research. | (L683 – strengths, limitations, and recommendations) One of the knowledge gaps revealed in this concept analysis is the lack of knowledge on what patients’ goals are set, how goal-oriented care is delivered, and how it is best put into practice in both one-on-one interactions between patients and providers and in interprofessional collaboration. ***Regarding patients it is important to gain more insight in how they are preferably prepared for discussing their personal goals.*** |
| Discussion | |  |  |
| 27 | Should be a bit wider not just in terms of the concept but how it fits in more widely. There is overall a lack of discussion around the issues of provider-provider collaboration (for example frequently care plans however holistically developed are not shared across providers, particularly those with different IT systems) and healthcare professional time (which would seem to be the major barrier) | This is an important remark as the provider-provider collaboration and the aspect of time of healthcare professionals seem to be important aspects that relate to goal-oriented care. However, no included articles mentioned something about collaboration nor time. It seems logic that providers need sufficient time to have a goal-oriented care conversation, etc. but for this discussion we have chosen to focus on what is described in the literature. | (L683 – strengths, limitations, and recommendations) One of the knowledge gaps revealed in this concept analysis is the lack of knowledge on what patients’ goals are set, how goal-oriented care is delivered, ***and how it is best put into practice in both one-on-one interactions between patients and providers and in interprofessional collaboration.*** Regarding patients it is important to gain more insight in how they are preferably prepared for discussing their personal goals.  (L689 – strengths, limitations, and recommendations) Initiating the development of an evaluation method could enable future intervention studies to gain more insight in the consequences of goal-oriented care and to make results comparable. ***This future research could provide insight in how effective goal-oriented care could potentially be which will be important to convince providers and policy makers of the benefits. These results might also inform the healthcare system in which resources they need to facilitate goal-oriented care***. |
| 28 | Couple of English corrections needed throughout: L109 chronical should be chronically, L211 independency should be independence | The corrections has been made. |  |

| **Comments from reviewer 2** | | **Response** | **Changes in the manuscript** |
| --- | --- | --- | --- |
| Results | |  |  |
| 29 | As the authors mentioned, there are similar concepts with Goal-oriented care. For example, Patient-Centered Clinical Method, Value-Based Practice or Expert Generalist Practice have proposed similar frameworks in primary care settings. I think the authors need to mention the difference between Goal-oriented care and the other concepts and why the authors chose Goal-oriented care in the Background and Discussion section. This is important for readers who are not familiar with Goal-oriented care. | This is an important remark as we did not elaborate on the related concepts. We consider this concept analysis as a first and main step in learning more about goal-oriented care. Based on this methodology and the results we were not able to make an overview of the differences and similarities of other concepts. Therefore, a critical review can be set up to perform in-depth comparison between the related concepts.  Also reviewer 1 (comment 9,10) and 3 (comment 34,35) have commented on the absence of the link with the related concepts. To address these comments, we have added more clarification on why we have chosen for the concept of goal-oriented care and where the other concepts could be placed. In addition, we addressed this gap in the recommendations. | (L95 – introduction) The concept of goal-oriented care has been launched and mentioned for the first time in 1991 by Mold who proposed the concept as an alternative way of providing care [7]. Later on, in 2012, Reuben and Tinetti took the concept of goal-oriented care a step forward by stating that care “must above all consider patients’ preferred outcomes” [8]. The focus on setting goals based on the patients’ needs and preferences rather than on health-related outcomes became one of the main novelties in chronic disease management [6]. ***Not only could goal-oriented care be proposed as an important paradigm to overcome some of the new challenges for chronical patients [2], it might also corresponded to the original concept of evidence based medicine (EBM) [9]. EBM was first published by Sackett in 1996 who described three key components: 1. best external evidence, 2. individual clinical expertise, and 3. patients’ values and expectations [9]. Since the first description of EBM, multiple approaches and paradigms has been developed to compromise between those three components [10]. For example, patient-centered care (PCC), which is already a well-known and widely used concept, is defined as “providing care that is respectful of, and responsive to individual patient preferences, needs, and values and ensuring that patients values guides all clinical decisions” [10]. Shared-decision making, on the other hand, also strives to share evidence and engage patients in care as it is “an approach where clinicians and patients share the best available evidence when faced with the task of making decisions, and where patients are supported to consider options, and to achieve informed preferences” [11]. Goal-oriented care is proposed as a promising healthcare paradigm and approach to operationalize EBM and return to where it all started [8]. However, in contrast to the other approaches and paradigms, goal-oriented care is ill defined.*** ***Developing a common understanding on the concept could potentially contribute to the clarification and in-depth comparison between the related concepts and eventually lead to better use in clinical practice. However,*** some healthcare providers might already assume that they practice goal-oriented care spontaneously, but there still is a lack of underpinning knowledge and guidance on how to provide goal-oriented care to patients [4, 5]. As a first step in exploring the potential of goal-oriented care in chronic care, it is important to gain in-depth knowledge on what goal-oriented care is about and how it can be generally described.  As goal-oriented care could be well-suited in primary care, as this context is often the linchpin for patients with chronic conditions, this will be the focus of this study [12]. This study aimed to describe a structured approach to deepen the concept of goal-oriented care for patients with chronic conditions or multimorbidity in the primary care context.  (702 – strengths, limitations, and recommendations) This workflow should include the skills and tools so patients and providers can find a common ground in the goals and are supported in implementing goal-oriented care in practice. ***Then, when goal-oriented care is a well understood concept it is possible to perform an in-depth comparison between related concepts (e.g. patient-centered care).*** |
| 30 | Please clarify how to “analyze” the included articles to define attributes in step 4. This can be helpful to understand the process of emerging codes for the readers who are not familiar with the concept analysis. | More clarification on the analyzing process is written down and an extra Table with an example of data extraction is given as illustration. This will provide more insight into the analyses. | ***(L219 – method: step 4) Similar to a qualitative, thematic analysis, the key articles were analyzed based on an open coding and then grouped into codes. (Table 3 – example of data analysis). These codes were then presented to and discussed with the co-authors. Based on this discussion codes were translated into attributes.*** |

|  | **Comments from reviewer 3** | **Response** | **Changes in the manuscript** |
| --- | --- | --- | --- |
| Method | |  |  |
| 31 | Regarding the methods I assume there is a good reason why related concepts including patient-centered care, shared decision making and value based medicine were not included in the search string, but could this be argued more clearly? | Indeed, these concepts were not included in the literature search. The method of a concept analysis prescribed to focus on deepening just one concept. This is made clearer in the manuscript.  However, since reviewer 1 (9,10) and 2 (28) also comment on the absence of elaborating on relating concepts (more or less) we have added more information on that in the introduction and discussion. | ***(L181 – method: select the literature) Related concepts such as patient-centered care, value-based care, etc. were not included as the method of concept analysis prescribes to deepen all the attributes of one concept.***  (L207 – method: step 3) Exclusion criteria: (a) focusing on single-disease management, (b) goals regarding disease-specific outcomes (e.g. cancer or diabetes), (c) focusing on goal-oriented care in a specific context (e.g. rehabilitation center), ***and (d) specifically mentioning patient-centered care, shared-decision making, etc. as they will hamper the understanding of specifically goal-oriented care.***  (L702 – discussion) This workflow should include the skills and tools so patients and providers can find a common ground in the goals and are supported in implementing goal-oriented care in practice. ***Then, when goal-oriented care is a well understood concept it is possible to perform an in-depth comparison between related concepts (e.g. patient-centered care).*** |

| Results | |  |  |
| --- | --- | --- | --- |
| 32 | On page 18: what is the confusion in the paragraph above exactly? Different aspects are mentioned, but what is the tension? | This was indeed unclear, to clarify more on this tension, the sentences have been rewritten. | ***(L357 – results: patients’ needs and preferences) Other authors recommended that goals should be a combination of both the patients’ goals and the providers’ goals which in turn is related to goal-negotiation [16, 20]. In conclusion, no overall understanding on the goals could be formulated.***  ***Besides this lack in understanding, there also seems to be ambiguity about the categorization of goals.*** Some authors emphasized that goals should contain core values of patients (e.g. the broader aspects that matter most to the patient) [6, 17] . |
| 33 | Regarding the results: Isn’t there literature that defines goal oriented by stating what it is ‘not’? | It is an interesting remark, but for this study we had to start from scratch to deepen the understanding of goal-oriented care. By using the method of a concept analysis we searched for what goal-oriented care ‘is’. The approach of learning about a concept trough what it is not is in the method of a concept analysis defined as contrary case. From this perspective, we have given information about what it is not, unfortunately we did not found any information in the literature about what it is not and we have therefore made no changes in the manuscript. | No changes have been made in the manuscript. |
| 34 | Cases: The cases are not fully readable in the version of the paper that I had access to, but I think you need to work a bit on them to make them more believable or better: find actual cases. E.g. case 1: for most CVD / DM patients cycling is actually recommended and case 3: obesity is hardly a barrier to travel. In reality I guess that most often patient’s goals are not dismissed but are simply not discussed (for many reasons). | Our excuses for the missing parts in the cases. Some changes have been made to be more realistic. We hope that this suits more now. | Joseph, 68- year old suffers from diabetes, hypertension and ***chronic obstructive pulmonary disease.***  Mary is a 40-year old mother of two young children and dealing with obesity since her childhood. ***Due to her weight, she has a lot of joints pain and is short of breath which limits her exercising capacity. Her children would love nothing more than their mother play with them. Unfortunately, she is not able to play soccer or jump on the trampoline because of the pain. The pain becomes too much for her and after long hesitation she discusses this with her physician. The only thing she wants is to play and interact with her children as painless as possible and therefore asks her physician to prescribe some medication. Her physician does not support medication, but instructs her to first strive for a*** ***healthy weight as a solution to relieve the pain. This is not aligned with the wishes of Mary who only wanted a short-term solution to be able to play with her children.*** In the end, she leaves the consultation room with a referral to a dietitian and sport coach. |
| 35 | Page 24: was there nothing about systems preparedness? If so, please that this was not found. | It is an interesting remark to also include system preparedness, but the current literature did not point to that. We have reread the articles and went back to the raw data, but no information could be found on system preparedness. We have described this shortcoming in the discussion. | **(L635 – discussion) *Besides patient and provider preparedness, it could seem logical that also the system has to be prepared, but the current literature do not point to that.*** |
| Discussion | |  |  |
| 36 | In the discussion I was expecting a juxtaposition of the goal oriented concept with related concepts in primary care, including generalism, holism, patient-centeredness, value based healthcare, shared decision making, patient participation, EBM (Sacket!) etc. How is it different? You could be more critical: is goal oriented care actually a better concept and if so why? | This is an important remark as we did not elaborate on the related concepts. We also considered ourselves to juxtapose goal-oriented care to related concepts, but the method of concept analysis does not allow that.  Though, we value your comment and especially concerning EBM and Sacket is a very interesting point, thank you for this. We have been thinking about it and we have added more information about how goal-oriented care link with EBM in the introduction. | (L95 – introduction) The concept of goal-oriented care has been launched and mentioned for the first time in 1991 by Mold who proposed the concept as an alternative way of providing care [7]. Later on, in 2012, Reuben and Tinetti took the concept of goal-oriented care a step forward by stating that care “must above all consider patients’ preferred outcomes” [8]. The focus on setting goals based on the patients’ needs and preferences rather than on health-related outcomes became one of the main novelties in chronic disease management [6]. ***Not only could goal-oriented care be proposed as an important paradigm to overcome some of the new challenges for chronical patients [2], it might also corresponded to the original concept of evidence based medicine (EBM) [9]. EBM was first published by Sackett in 1996 who described three key components: 1. best external evidence, 2. individual clinical expertise, and 3. patients’ values and expectations [9]. Since the first description of EBM, multiple approaches and paradigms has been developed to compromise between those three components [10]. For example, patient-centered care (PCC), which is already a well-known and widely used concept, is defined as “providing care that is respectful of, and responsive to individual patient preferences, needs, and values and ensuring that patients values guides all clinical decisions” [10]. Shared-decision making, on the other hand, also strives to share evidence and engage patients in care as it is “an approach where clinicians and patients share the best available evidence when faced with the task of making decisions, and where patients are supported to consider options, and to achieve informed preferences” [11]. Goal-oriented care is proposed as a promising healthcare paradigm and approach to operationalize EBM and return to where it all started [8]. However, in contrast to the other approaches and paradigms, goal-oriented care is ill defined.*** ***Developing a common understanding on the concept could potentially contribute to the clarification and in-depth comparison between the related concepts and eventually lead to better use in clinical practice. However,*** some healthcare providers might already assume that they practice goal-oriented care spontaneously, but there still is a lack of underpinning knowledge and guidance on how to provide goal-oriented care to patients [4, 5]. As a first step in exploring the potential of goal-oriented care in chronic care, it is important to gain in-depth knowledge on what goal-oriented care is about and how it can be generally described.  As goal-oriented care could be well-suited in primary care, as this context is often the linchpin for patients with chronic conditions, this will be the focus of this study [12]. This study aimed to describe a structured approach to deepen the concept of goal-oriented care for patients with chronic conditions or multimorbidity in the primary care context.  (702 – strengths, limitations, and recommendations) This workflow should include the skills and tools so patients and providers can find a common ground in the goals and are supported in implementing goal-oriented care in practice. ***Then, when goal-oriented care is a well understood concept it is possible to perform an in-depth comparison between related concepts (e.g. patient-centered care).*** |
| 37 | Perhaps also provide a discussion on tensions with contrasting (1) frameworks and (3) systems such as transnationalism, (4) care standards, P4P, neoliberal economics and budgeting (tension of providing efficient care for many). | It is a very interesting take to discuss (1) the tension with contrasting frameworks, but in this study we solely focused on an objective description of the information that was provided in the current literature. Little more attention on the link with the (2) care standards is provided in the introduction and discussion.  The absence of this juxtaposition with other frameworks is added in the discussion section as recommendation for further research.  To discuss a bit more on (3) contrasting systems, a cross-comparison study has been published after submitting this study. In this study, three international cases has adopted goal-oriented care and were linked it to integrated care. They found that goal-oriented could enable integrated care. It can be suggested that goal-oriented care has the potential to transit over specific health system and integrate them to each other [21].  We also value your comment to elaborate more on the (4) economic aspects of goal-oriented care. However, to investigate potential economic effects for goal-oriented care, further research is necessary. At this moment there is a discussion in the field about how to evaluate goal-oriented care. We have addressed this in the discussion section. | ***(L181 – method: select the literature) Related concepts such as patient-centered care, value-based care, etc. were not included as the method of concept analysis prescribes to deepen all the attributes of one concept.***  (L207 – method: step 3) Exclusion criteria: (a) focusing on single-disease management, (b) goals regarding disease-specific outcomes (e.g. cancer or diabetes), (c) focusing on goal-oriented care in a specific context (e.g. rehabilitation center), ***and (d) specifically mentioning patient-centered care, shared-decision making, etc. as they will hamper the understanding of specifically goal-oriented care.***  (L95 – introduction) The concept of goal-oriented care has been launched and mentioned for the first time in 1991 by Mold who proposed the concept as an alternative way of providing care [7]. Later on, in 2012, Reuben and Tinetti took the concept of goal-oriented care a step forward by stating that care “must above all consider patients’ preferred outcomes” [8]. The focus on setting goals based on the patients’ needs and preferences rather than on health-related outcomes became one of the main novelties in chronic disease management [6]. ***Not only could goal-oriented care be proposed as an important paradigm to overcome some of the new challenges for chronical patients [2], it might also corresponded to the original concept of evidence based medicine (EBM) [9]. EBM was first published by Sackett in 1996 who described three key components: 1. best external evidence, 2. individual clinical expertise, and 3. patients’ values and expectations [9]. Since the first description of EBM, multiple approaches and paradigms has been developed to compromise between those three components [10]. For example, patient-centered care (PCC), which is already a well-known and widely used concept, is defined as “providing care that is respectful of, and responsive to individual patient preferences, needs, and values and ensuring that patients values guides all clinical decisions” [10]. Shared-decision making, on the other hand, also strives to share evidence and engage patients in care as it is “an approach where clinicians and patients share the best available evidence when faced with the task of making decisions, and where patients are supported to consider options, and to achieve informed preferences” [11]. Goal-oriented care is proposed as a promising healthcare paradigm and approach to operationalize EBM and return to where it all started [8]. However, in contrast to the other approaches and paradigms, goal-oriented care is ill defined.*** ***Developing a common understanding on the concept could potentially contribute to the clarification and in-depth comparison between the related concepts and eventually lead to better use in clinical practice. However,*** some healthcare providers might already assume that they practice goal-oriented care spontaneously, but there still is a lack of underpinning knowledge and guidance on how to provide goal-oriented care to patients [4, 5]. As a first step in exploring the potential of goal-oriented care in chronic care, it is important to gain in-depth knowledge on what goal-oriented care is about and how it can be generally described.  As goal-oriented care could be well-suited in primary care, as this context is often the linchpin for patients with chronic conditions, this will be the focus of this study [12]. This study aimed to describe a structured approach to deepen the concept of goal-oriented care for patients with chronic conditions or multimorbidity in the primary care context.  (L702 – discussion) This workflow should include the skills and tools so patients and providers can find a common ground in the goals and are supported in implementing goal-oriented care in practice. ***Then, when goal-oriented care is a well understood concept it is possible to perform an in-depth comparison between related concepts (e.g. patient-centered care).*** |
| 38 | I would also like to see what was missing in the literature / what was not mentioned? E.g. as I mentioned above: system preparedness (as opposed to patient and provider preparedness). Anything else? | Indeed, their remain some knowledge gaps after analyzing the current literature. The literature learned us that goal-oriented is a stepwise approach, but it is still unclear how this theory should be translated into a practical approach including the organization of patient preparedness and interprofessional collaboration. Besides the lack of a practical workflow, there is also a lack on how to develop process indicators to eventually evaluate a goal-oriented care practice. Also, to meet the previous comment of juxtaposing goal-oriented care with other frameworks and systems, more research will be needed as the current literature could not provide this answer. To conclude that this concept analysis is first main step to facilitate further research on divers topics related to goal-oriented care. | ***(L 635 – discussion) Besides patient and provider preparedness, it could seem logical that also the system has to be prepared, but the current literature do not point to that.***  ***(L683 – strengths, limitations, and recommendations) One of the knowledge gaps revealed in this concept analysis is the lack of knowledge on what patients’ goals are set, how goal-oriented care is delivered, and how it is best put into practice in both one-on-one interactions between patients and providers and in interprofessional collaboration.*** Regarding patients it is important ***to gain more insight in how they are preferably prepared for discussing their personal goals.***  ***(L700 – strengths, limitations, and recommendations) However, further research is needed to translate the current knowledge on the concept of goal-oriented care into a tangible workflow process of care that entails the three stages.*** |

| **Comments of reviewer 4** | | **Response** | **Changes in the manuscript** |
| --- | --- | --- | --- |
| Results | |  |  |
| 39 | This case (Mary) feels a bit tenuous - it feels unlikely a family doctor would not 'allow' travel plans on reasonable grounds. I understand what you are trying to illustrate - but a better example is needed. | Thank you for this remark. Also reviewer 1, 2, and 3 (24,32) made this comment. A more realistic example (checked with GP) is described. | Mary is a 40-year old mother of two young children and dealing with obesity since her childhood. ***Due to her weight, she has a lot of joints pain and is short of breath which limits her exercising capacity. Her children would love nothing more than their mother play with them. Unfortunately, she is not able to play soccer or jump on the trampoline because of the pain. The pain becomes too much for her and after long hesitation she discusses this with her physician. The only thing she wants is to play and interact with her children as painless as possible and therefore asks her physician to prescribe some medication. Her physician does not support medication, but instructs her to first strive for a healthy weight as a solution to relieve the pain. This is not aligned with the wishes of Mary who only wanted a short-term solution to be able to play with her children.*** In the end, she leaves the consultation room with a referral to a dietitian and sport coach. |
| 40 | (L220) This is a really important point, and the likely reason for resistance by providers.   to goal-oriented care. It feels like it would be of value to expand on this further if possible. Did the literature explore this in further depth - what role does the provider have in shifting patients' goals and how should they do this, what are the ethics behind trying to do so, how is this best done in a way that does not alienate the patient? | We are aware that we have not further discussed this point. It is indeed really important to explore this more in depth, because we believe that we first have to answer these questions before we can fully implement goal-oriented care in practice. The current literature could not provide us an answer on this. This comment is also about how we can translate the patients’ (life) goals to the providers’ (medical) goals and eventually develop a workflow for goal-oriented care. Unfortunately, this concept analysis could not provide us with the needed information to answer these questions and provided an objective description of what we found in literature.  We thank you for this comment, you really touched very important points regarding goal-oriented care and these topics will be subject to further research. We have added this ideas in the discussion section as recommendations for further research. | (L699 – strengths, limitations, and recommendations) Goal-oriented care shows the potential to be a way forward for patients with chronic conditions and multimorbidity. However, further research is needed to translate the current knowledge on the concept of goal-oriented care into a tangible workflow process of care that entails the three stages. ***This workflow should include the skills and tools so patients and providers can find a common ground in the goals and are supported in implementing goal-oriented care in practice.*** |
| 41 | The term 'empirical referent' is hard to understand, although I can see you have described it - in order to bring it to life I wonder if the table with the examples could provide more details. | The term empirical referent is indeed complex to understand, but we have chosen to stick to the prescribed steps of Walker and Avant and their naming. To clarify this a bit more, the purpose of each tool or measurement and for who it can be used has been added in the overview. | Adjustments have been made to Table 4 with more details about the purpose of each tool, measurement. |
| Discussion | |  |  |
| 42 | I wondered if a bit more discussion is needed about the risks of when patient and providers goals do not align, and approaches needed to align these. | It is interesting to deepen potential risks in case that patient and provider goals do not align. At this moment, the literature does not address this risk and could therefore be further explored. What we do know is that, in case that those goals do not align, it could cause conflicts between patients and providers. We described those potential conflicts and strategies to overcome them in the discussion part. | ***(L591 – discussion) Not only is there ambiguity on what goals patients set, it is also not clear how goals are being set. What is clear is that patients and providers must collaborate and negotiate on which goals are important. Nevertheless, this can still cause conflicts between the patients’ goals and providers’ goals [22, 23]. To overcome these conflicts, it is suggested to first set the patients’ goals and then discuss about the medical goals, because conflicts are more likely when goals are placed on the same level [24]. It should however be noted that setting the patients’ goals on top does not legitimate full patients’ responsibility over the care plan [24]. Another way to overcome these conflicts is to work with a facilitator as Naik et al. did in developing their patients priorities identification process. These facilitators supported patients in setting goals, choosing the most important goals to eventually communicate*** ***them with the provider [6]. Yet another strategy is to use tools to assess patient treatment priorities and preferences.*** Unfortunately, Mangin et al. found few relevant tools to set patients’ goals [19]. They argue for the need to develop specific strategies to make patient priorities visible in the clinical record and medical-decision making [19]. |
| 43 | (L253) What if any, guidance is there for providers to work out how to align patient needs and preference with those of their health and the health system. | It is an interesting question as this will be one of the main challenges to eventually implement goal-oriented care in practice. In literature that is not clearly described, but some strategies have been proposed. These strategies have been added in the discussion (cfr. answer previous comment) | ***(L591 – discussion) Not only is there ambiguity on what goals patients set, it is also not clear how goals are being set. What is clear is that patients and providers must collaborate and negotiate on which goals are important. Nevertheless, this can still cause conflicts between the patients’ goals and providers’ goals [22, 23]. To overcome these conflicts, it is suggested to first set the patients’ goals and then discuss about the medical goals, because conflicts are more likely when goals are placed on the same level [24]. It should however be noted that setting the patients’ goals on top does not legitimate full patients’ responsibility over the care plan [24]. Another way to overcome these conflicts is to work with a facilitator as Naik et al. did in developing their patients priorities identification process. These facilitators supported patients in setting goals, choosing the most important goals to eventually communicate*** ***them with the provider [6]. Yet another strategy is to use tools to assess patient treatment priorities and preferences.*** Unfortunately, Mangin et al. found few relevant tools to set patients’ goals [19]. They argue for the need to develop specific strategies to make patient priorities visible in the clinical record and medical-decision making [19].  Goal-oriented care shows the potential to be a way forward for patients with chronic conditions and multimorbidity. However, further research is needed to translate the current knowledge on the concept of goal-oriented care into a tangible workflow process of care that entails the three stages. ***This workflow should include the skills and tools so patients and providers can find a common ground in the goals and are supported in implementing goal-oriented care in practice.*** |
| 44 | (L323) This sentence is a broad generalization. Could it be that sometime or often this happens... not always. | The sentence is written more ‘carefully’. | Currently patients ***are not always stimulated*** to think about their care. |
| 45 | (L327) This is a broad generalization, I think there are GPs who are aware of these. Again could the word often or sometimes be used to moderate the sentence? | As the previous comment, the word ‘often’ is added. | Voigt et al. observed that GPs ***are often unaware*** of patients’ priorities in daily life, which were in contrast with their perceived importance of patient’s medical goals |
| 46 | (337) This sentence seems to contradict the next one (limited studies, and then is says mostly positive outcomes). | The positive outcomes were related to the results of the founded studies. This has been made more clear. | (L638 – discussion) In terms of the consequences of goal-oriented care, ***a limited number of studies have been able to demonstrate outcomes of goal-oriented care. Nonetheless, these studies showed mostly*** positive outcomes towards the patients, providers, health system, and overall population well-being. |
| 47 | Supporting files: These supplementary documents are helpful and interesting- can you make reference to them in the main text so the reader knows when to refer to them. | We have checked the references to the supporting files and added if we needed to. | (L262 – results) goal-oriented care ***(S1 Table 1)***  (L 439 – antecedents) in each training ***(S2 Table 2).*** |

1.	Levac D, Colquhoun H, O'Brien KK. Scoping studies: advancing the methodology. Implement Sci. 2010;5:69. Epub 2010/09/22. doi: 10.1186/1748-5908-5-69. PubMed PMID: 20854677; PubMed Central PMCID: PMCPMC2954944.

2.	Kuluski K, Peckham A, Gill A, Gagnon D, Wong-Cornall C, McKillop A, et al. What is Important to Older People with Multimorbidity and Their Caregivers? Identifying Attributes of Person Centered Care from the User Perspective. Int J Integr Care. 2019;19(3):4. Epub 2019/08/02. doi: 10.5334/ijic.4655. PubMed PMID: 31367203; PubMed Central PMCID: PMCPMC6659759.

3.	Kuipers SJ, Cramm JM, Nieboer AP. The importance of patient-centered care and co-creation of care for satisfaction with care and physical and social well-being of patients with multi-morbidity in the primary care setting. BMC Health Serv Res. 2019;19(1):1-9.

4.	Vermunt N, Harmsen M, Westert GP, Olde Rikkert MGM, Faber MJ. Collaborative goal setting with elderly patients with chronic disease or multimorbidity: a systematic review. BMC Geriatr. 2017;17(1):167. Epub 2017/08/02. doi: 10.1186/s12877-017-0534-0. PubMed PMID: 28760149; PubMed Central PMCID: PMCPMC5537926.

5.	Poitras ME, Maltais ME, Bestard-Denomme L, Stewart M, Fortin M. What are the effective elements in patient-centered and multimorbidity care? A scoping review. BMC Health Serv Res. 2018;18(1):446. Epub 2018/06/15. doi: 10.1186/s12913-018-3213-8. PubMed PMID: 29898713; PubMed Central PMCID: PMCPMC6001147.

6.	Naik AD, Dindo LN, Van Liew JR, Hundt NE, Vo L, Hern, et al. Development of a Clinically Feasible Process for Identifying Individual Health Priorities. Journal of the American Geriatrics Society. 2018;66(10):1872-9. PubMed PMID: rayyan-47069467.

7.	Mold JW, Blake GH, Becker LA. Goal-oriented medical care. Family medicine. 1991;23(1):46-51. Epub 1991/01/01. PubMed PMID: 2001782.

8.	Reuben DB, Tinetti ME. Goal-oriented patient care--an alternative health outcomes paradigm. The New England journal of medicine. 2012;366(9):777-9. Epub 2012/03/02. doi: 10.1056/NEJMp1113631. PubMed PMID: 22375966.

9.	Sackett DL, Rosenberg WM, Gray JM, Haynes RB, Richardson WS. Evidence based medicine: what it is and what it isn't. British Medical Journal Publishing Group; 1996.

10.	Baker A. Crossing the quality chasm: a new health system for the 21st century: British Medical Journal Publishing Group; 2001.

11.	Elwyn G, Frosch D, Thomson R, Joseph-Williams N, Lloyd A, Kinnersley P, et al. Shared decision making: a model for clinical practice. Journal of general internal medicine. 2012;27(10):1361-7.

12.	Hobbs FR, Bankhead C, Mukhtar T, Stevens S, Perera-Salazar R, Holt T, et al. Clinical workload in UK primary care: a retrospective analysis of 100 million consultations in England, 2007–14. The Lancet. 2016;387(10035):2323-30.

13.	Mold. Goal-directed health care: redefining health and health care in the era of value-based care. Cureus 2017;9(2).

14.	Berntsen G, Hoyem A, Lettrem I, Ruland C, Rumpsfeld M, Gammon D. A person-centered integrated care quality framework, based on a qualitative study of patients' evaluation of care in light of chronic care ideals. BMC Health Serv Res. 2018;18(1):479. Epub 2018/06/22. doi: 10.1186/s12913-018-3246-z. PubMed PMID: 29925357; PubMed Central PMCID: PMCPMC6011266.

15.	Salter C, Shiner A, Lenaghan E, Murdoch J, Ford JA, Winterburn S, et al. Setting goals with patients living with multimorbidity: qualitative analysis of general practice consultations. Br J Gen Pract. 2019;69(684):e479-e88. Epub 2019/06/05. doi: 10.3399/bjgp19X704129. PubMed PMID: 31160370; PubMed Central PMCID: PMCPMC6592350.

16.	Kessler D, Walker I, Sauve-Schenk K, Egan M. Goal setting dynamics that facilitate or impede a client-centered approach. Scandinavian journal of occupational therapy. 2018;26(5):315-24. PubMed PMID: 29671662.

17.	Tinetti ME, Esterson J, Ferris R, Posner P, Blaum CS. Patient Priority-Directed Decision Making and Care for Older Adults with Multiple Chronic Conditions. Clinics in geriatric medicine. 2016;32(2):261-75. PubMed PMID: 27113145.

18.	Murdoch J, Salter C, Ford J, Lenaghan E, Shiner A, Steel N. The "unknown territory" of goal-setting: Negotiating a novel interactional activity within primary care doctor-patient consultations for patients with multiple chronic conditions. Soc Sci Med. 2020;256:113040. Epub 2020/05/31. doi: 10.1016/j.socscimed.2020.113040. PubMed PMID: 32473530; PubMed Central PMCID: PMCPMC7306159.

19.	Mangin D, Stephen G, Bismah V, Risdon C. Making patient values visible in healthcare: a systematic review of tools to assess patient treatment priorities and preferences in the context of multimorbidity. BMJ open. 2016;6(6):e010903.

20.	Kuluski K, Guilcher SJT. Toward a Person-Centred Learning Health System: Understanding Value from the Perspectives of Patients and Caregivers. HealthcarePapers. 2019;18(4):36-46. Epub 2020/01/05. doi: 10.12927/hcpap.2019.26030. PubMed PMID: 31901067.

21.	Steele Gray C, Grudniewicz A, Armas A, Mold J, Im J, Boeckxstaens P. Goal-Oriented Care: A Catalyst for Person-Centred System Integration. Int J Integr Care. 2020;20(4):8. Epub 2020/11/18. doi: 10.5334/ijic.5520. PubMed PMID: 33199976; PubMed Central PMCID: PMCPMC7646288.

22.	Bayliss EA, Bonds DE, Boyd CM, Davis MM, Finke B, Fox MH, et al. Understanding the context of health for persons with multiple chronic conditions: moving from what is the matter to what matters. Annals of family medicine. 2014;12(3):260-9. Epub 2014/05/14. doi: 10.1370/afm.1643. PubMed PMID: 24821898; PubMed Central PMCID: PMCPMC4018375.

23.	Fried TR, Tinetti M, Agostini J, Iannone L, Towle V. Health outcome prioritization to elicit preferences of older persons with multiple health conditions. Patient education and counseling. 2011;83(2):278-82.

24.	Berntsen GK, Gammon D, Steinsbekk A, Salamonsen A, Foss N, Ruland C, et al. How do we deal with multiple goals for care within an individual patient trajectory? A document content analysis of health service research papers on goals for care. BMJ open. 2015;5(12):e009403. Epub 2015/12/15. doi: 10.1136/bmjopen-2015-009403. PubMed PMID: 26656243; PubMed Central PMCID: PMCPMC4679896.
