# Supplementary material for: Goal-oriented care for patients with chronic conditions or multimorbidity in primary care: A scoping review and concept analysis
Source: PLoS One. 2022 Feb 4;17(2):e0262843. doi: 10.1371/journal.pone.0262843 (PMC8815876; doi:10.1371/journal.pone.0262843)
Supplement: Attachment — Submitted filename: Response to the reviewers.docx [file pone.0262843.s007.docx]

Dear Editor
Dear Reviewers

Thank you for the positive feedback and pointing to minor comments to improve our manuscript.
Please find our answers in the Table below and in the revised manuscript.

Sincerely
Dagje Boeykens, on behalf of the co-authors

|  | **Comments from the editor** | **Response** | **Changes in the manuscript** |
| --- | --- | --- | --- |
| **1** | Please review your reference list to ensure that it is complete and correct. If you have cited papers that have been retracted, please include the rationale for doing so in the manuscript text, or remove these references and replace them with relevant current references. Any changes to the reference list should be. | We have checked each reference by searching them in PubMed and identified 1 article that has been retracted. This article had no major contribution to the manuscript and is therefore removed as a reference; adjustment to the reference list have been made. No further issues regarding the reference list have been identified when checking in PubMed. | Reference 61: ‘Locke EA, Latham GP. A theory of goal-setting and task performance’ has been removed from the manuscript. |
|  | **Comments from reviewer 4** | **Response** | **Changes in the manuscript** |
| **1** | However, I also feel strongly that your 3 case examples need to be further strengthened with detail and reference back to the attributes (or absence of them) of goal-oriented care in table 4. It is quite a hard concept to grasp and the case studies are your opportunity to bring it to life - but I don't think they currently fully do. | We agree that it is indeed hard to grasp the concept. Because it is not possible to provide new cases, we discussed with the group how we could address this comment. We have tried to make the model case (Joseph) and additional case (Ben) more illustrative by labelling them with the corresponding attributes and sub attributes. For the additional case of Ben this illustrated the lack of the ‘goal-evaluation attribute’.  Besides the labelling, we have added some sentences to increase clarity. | L 421 The first case of Joseph (Box 1) encompasses all the attributes identified in the literature and is therefore identified as a model case. To make this case more lively, each attribute and sub attribute of goal-oriented care is labelled in the box.  **Case Joseph**  Joseph, 68- year old suffers from diabetes, hypertension and chronic obstructive pulmonary disease. Throughout his entire working life, he was a secondary school teacher. He has been retired for three years now *(patients’ context)*. Despite the fact that he is limited by his health condition, he loves spending time gardening and playing with his grandchildren *(patients’ needs and preferences)*. A few years ago he was a passionate cyclist, but his racing bike has been stored for a long time now. His friends encourage him to cycle with them on a weekly base *(patients’ context)*. His wife supports this initiative and argues that this will be beneficial for his social contact *(patients’ context)*. Every month Joseph visits his family doctor for a check-up. For each consultation, he prepares a list of things he wants to discuss. He has the chance to share his story in an open communication in which trust and mutual respect are key components *(goal-elicitation)*. In his monthly check-up with his family doctor he suggests his wishes to cycle again with his friends *(patients’ needs and preferences & goal-setting; interaction)*. His doctor doubts whether this will be possible and after discussion and negotiation *(goal-setting; interaction)*, they plan that he would join his friends in their weekly cycling trip but only for the first two hours *(goal-setting; foundation for SMART goal)*. The group will be asked to adapt their pace and Joseph will make sure that he does not need to return back home on his own *(patients’ context)*. The doctor makes adjustments to the medication scheme according to the increased efforts Joseph will make *(goal-setting; care plan)*. He will also contact the cardiologist to inform him about the changes to the medication schema *(goal-setting; care plan)*. The family doctor and the cardiologist will collaborate in order to succeed in Joseph’s goal *(goal-setting; care delivery)*. The family doctor and Joseph agree to discuss and evaluate the course after three months *(goal-evaluation; feedback & evaluation)* . It is possible to increase or decrease the intensity depending on Joseph's health state and his own preferences *(goal-evaluation; evaluation)*.  **Case Ben**  Ben, a 30-year old man, was renovating a house that he bought with his girlfriend when he was diagnosed with MS *(patients’ context)*. They made plans to marry next year and to make a world trip as honeymoon. These plans have been put aside due to the recent diagnosis. Although he was feeling down and did not have the energy to do anything he ended up with his physician. Initiated by the interaction and the conversation with his physician *(goal-elicitation)* he was enabled to set goals again and to look at the future *(goal-setting; interaction)*. The physician decided to discuss the things that Ben really likes to do as for example making travel plans and would make it possible to achieve his goals *(goal-setting; interaction)*. Although a plan has been devised towards Ben’s goals *(goal-setting; care plan)*, there has never been an discussion whether or not the goals were achieved or required adjustments to new capabilities of Ben. For this reason the consultation Ben had with his physician could not labelled as goal-oriented care |
| **2** | Language editing | Thank you for pointing to some language issues. They have been corrected. |  |
